# Complexation of 2,6-helic[6]arene and its derivatives with 1,1′-dimethyl-4,4′-bipyridinium salts and protonated 4,4'-bipyridinium salts: an acid–base controllable complexation

**DOI:** 10.3762/bjoc.15.173

**Published:** 2019-07-26

**Authors:** Jing Li, Qiang Shi, Ying Han, Chuan-Feng Chen

**Affiliations:** 1 Beijing National Laboratory for Molecular Sciences, CAS Key Laboratory of Molecular Recognition and Function, Institute of Chemistry, Chinese Academy of Sciences, Beijing 100190, China; 2 University of Chinese Academy of Sciences, Beijing 100049, China

**Keywords:** 4,4'-bipyridinium salts, complexation, helic[6]arene, hydrogen bond, macrocycles, macrocyclic arene

## Abstract

2,6-Helic[6]arene and its derivatives were synthesized, and their complexation with 1,1′-dimethyl-4,4′-bipyridinium and protonated 4,4'-bipyridinium salts were investigated in detail. It was found that the helic[6]arene and its derivatives could all form 1:1 complexes with both 1,1′-dimethyl-4,4'-bipyridinium salts and protonated 4,4'-bipyridinium salts in solution and in the solid state. Especially, the helic[6]arene and its derivatives containing 2-hydroxyethoxy or 2-methoxyethoxy groups exhibited stronger complexation with the guests than the other helic[6]arene derivatives for the additional multiple hydrogen bonding interactions between the hosts and the guests, which were evidenced by ^1^H NMR titrations, X-ray crystal structures and DFT calculations. Moreover, it was also found that the association constants (*K*_a_) of the complexes could be significantly enhanced with larger counteranions of the guests and in less polar solvents. Furthermore, the switchable complexation between the helic[6]arene and protonated 4,4'-bipyridinium salt could be efficiently controlled by acids and bases.

## Introduction

Macrocyclic host molecules [1–2] play a significant role in host–guest chemistry. Compared with noncyclic molecules, the structures of macrocyclic hosts can greatly enhance the host–guest complexation ability through preorganization. Moreover, cyclic structures are also the epitome of complex-binding pockets of enzymes [3]. Macrocyclic arenes including calixarenes [4–5], resorcinarenes [6], cyclotriveratrylenes [7–8], pillararenes [9], biphen[*n*]arenes [10] and others [11–12] are all composed of hydroxy-substituted aromatic rings bridged by methylene or methenyl groups. They have been a kind of important macrocyclic host molecules during the last decades due to their unique structures and a wide range of applications in host–guest chemistry [13–18], self-assembly [19], biomedicine [20] and materials science [21–22]. The derivatives of macrocyclic arenes with diverse functional groups are also important for the development of various new host–guest supramolecular systems [23–29].

**Figure 1:**
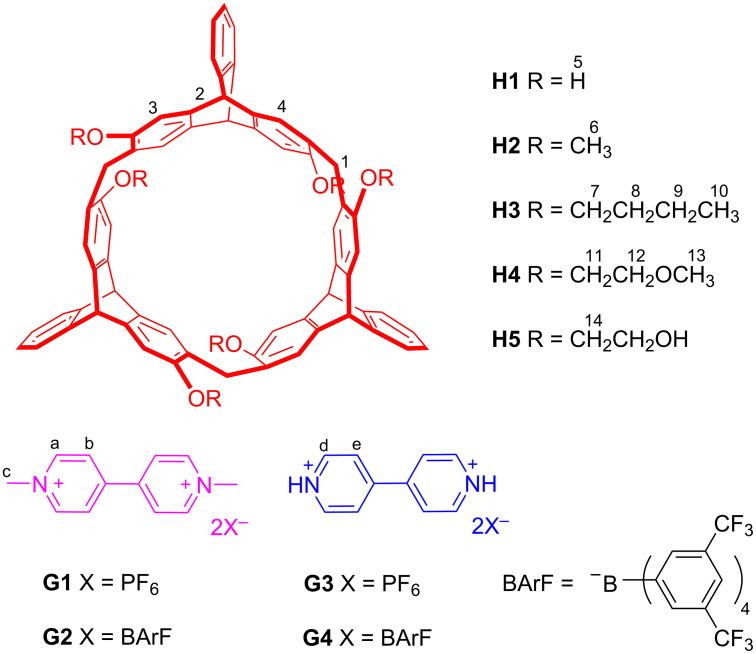
Structures and proton designations of hosts **H1**–**5** and guests **G1**–**4**.

Helic[6]arenes [30], a new kind of macrocyclic arenes, are composed of 2,6-dihydroxy-substituted triptycene subunits bridged by methylene groups. They have exhibited wide potential applications in supramolecular chemistry [31–36] for their unique structures and electron-rich cavities. In this paper, we report the complexation between 2,6-helic[6]arene and its four derivatives with 1,1′-dimethyl-4,4′-bipyridinium and protonated 4,4'-bipyridinium salts (Figure 1) in both solution and in the solid state. We found that the helic[6]arene and its derivatives containing 2-hydroxyethoxy or 2-methoxyethoxy groups showed stronger complexation with the guests than the other helic[6]arene derivatives. This result can be explained by the additional multiple hydrogen-bonding interactions between the hosts and the guests, which were evidenced by ^1^H NMR titration, X-ray crystal structures and DFT calculations. Moreover, we also found that the *K*_a_ values of the complexes could be significantly enhanced with larger counteranions of the guests and in less polar solvent. Furthermore, the controllable complexation between (*O*-methyl)_6_-2,6-helic[6]arene and protonated 4,4'-bipyridinium salt could be efficiently controlled by acids and bases.

## Results and Discussion

### Synthesis of the hosts and the guests

**Scheme 1:**
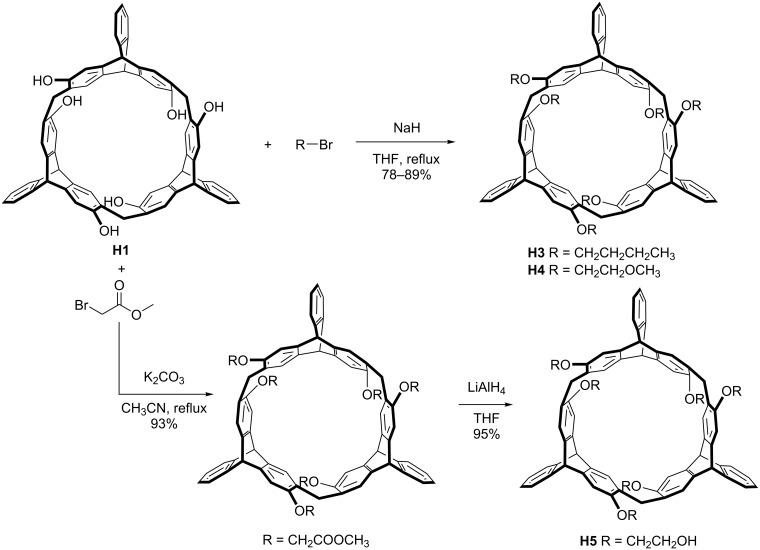
Synthesis of hosts **H3**–**5**.

2,6-Helic[6]arene **H1** and its methyl-substituted derivative **H2** were prepared according to previously reported methods [30]. Starting from helic[6]arene **H1**, helic[6]arene derivatives **H3** and **H4** were conveniently synthesized by etherification of **H1** with bromobutane or 2-bromoethyl methyl ether, respectively, in tetrahydrofuran in the presence of sodium hydride. Helic[6]arene derivative **H5** was synthesized by treatment of **H1** with methyl bromoacetate followed by reduction with lithium aluminium hydride (Scheme 1). The guests **G1**–**3** were prepared according to previously reported procedures [37–39]. Guest **G4** was synthesized through reaction of 4,4′-bipyridine with concentrated HCl in acetonitrile followed by counteranion exchange with sodium tetrakis[3,5-di(trifluoromethyl)phenyl]borate (NaBArF) in dichloromethane. The new compounds were confirmed by NMR spectroscopy and high-resolution mass spectrometry (Supporting Information File 1, Figures S1–S8).

### Host–guest complexation in solution

**Figure 2:**
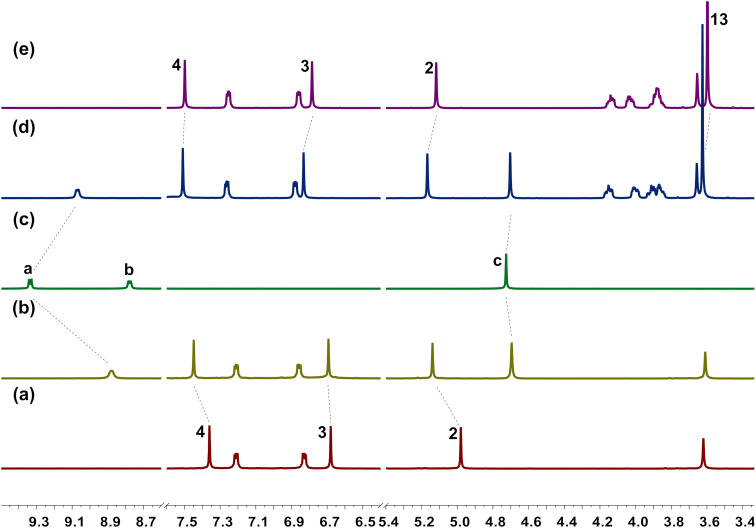
Partial ^1^H NMR spectra (400 MHz CDCl_3_/acetone-*d*_6_ 1:2 (v/v), 298 K) of (a) free **H1**, (b) **H1** with 1.0 equiv **G1**, (c) free **G1**, (d) **H4** with 1.0 equiv **G1**, (e) free **H4**. [**H1**]_0_ = [**H4**]_0_ = [**G1**]_0_ = 2.0 mM.

Firstly, we tested the complexation between hosts **H1** and **H4** with guest **G1** in solution by ^1^H NMR spectroscopy. As shown in Figure 2, when mixing equivalent amounts of host and guest in CDCl_3_/acetone-*d*_6_ 1:2 (v/v), the ^1^H NMR spectrum showed a new set of proton signals, which was different from the free host or guest, indicating the formation of new complexes **H1·G1** and **H4·G1**, respectively, and the complexation was a fast exchange process on the NMR time scale. The proton signals of a and c of the bipyridinium ring showed upfield shifts, while the signal for protons b completely disappeared due to the shielding effect of the aromatic rings in hosts **H1** or **H4**. The signals for the protons 2, 3, and 4 of **H1** and 2, 3, 4, and 13 of **H4** all showed downfield shifts, which might be attributed to the deshielding effect of guest **G1**. Other helic[6]arene derivatives (**H2**, **H3**, **H5**) with guests **G1** and **G2** showed similar complexation as described above (Supporting Information File 1, Figures S9–S14).

**Figure 3:**
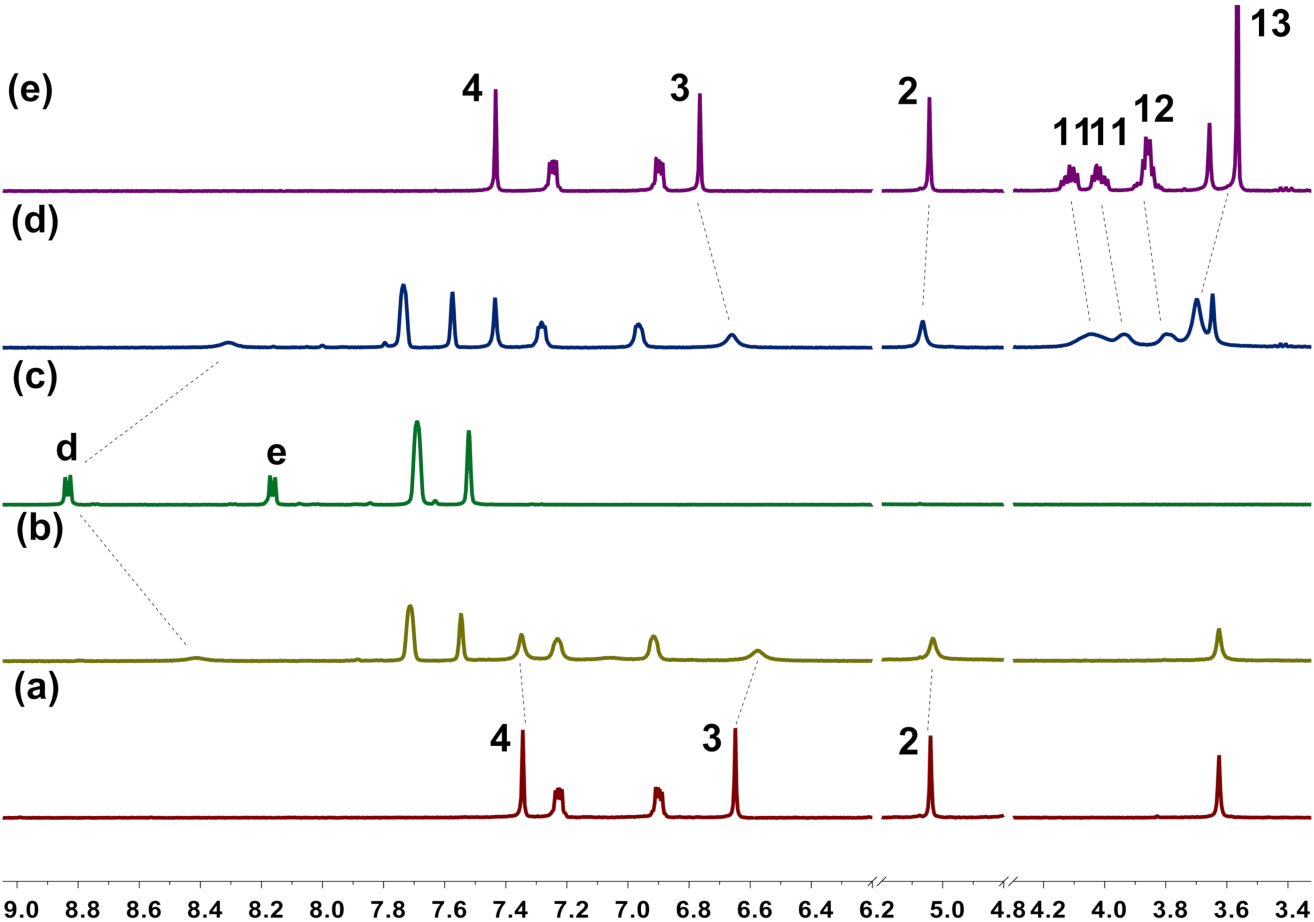
Partial ^1^H NMR spectra (400 MHz, CD_2_Cl_2_, 298 K) of (a) free **H1**, (b) **H1** with 1.0 equiv **G4**, (c) free **G4**, (d) **H4** with 1.0 equiv **G4**, (e) free **H4**. [**H1**]_0_ = [**H4**]_0_ = [**G4**]_0_ = 2.0 mM.

We also investigated the complexation between hosts **H1** and **H4** with guest **G4** in solution by ^1^H NMR spectroscopy. As shown in Figure 3, upon mixing equal equivalents of host and guest in CD_2_Cl_2_, the ^1^H NMR spectrum also showed a new set of proton signals, which was different from the free host or guest. These results indicated that the new complexes **H1·G4** and **H4·G4** were formed, and the complexation between the host and the guest was a fast exchange process on the NMR time scale as well. The signal for protons d of the 4,4'-bipyridinium ring showed an upfield shift and that for protons e completely disappeared possibly due to the shielding effect of the aromatic rings in **H1** or **H4**. The proton signals of 2 and 3 of **H1** and 3 of **H4** all showed upfield shifts with broadened peaks, which indicated that π–π stacking interactions between the bipyridinium unit of **G4** and the benzene ring of the hosts might exist. The signals for protons 2 and 13 of **H4** showed a downfield shift with broadened signals due to deshielding effect, while the signals for protons 11 and 12 showed upfield shifts, possibly due to hydrogen bonding between the hydrogen of the bipyridinium unit of **G4** and the oxygen atoms of the host. Similarly, the complexation between other helic[6]arene derivatives (**H2**, **H3**, **H5**) with guests **G3** and **G4** could also be observed (Supporting Information File 1, Figures S15–S22). Furthermore, job plots showed that throughout 1:1 host–guest complexes are formed (Supporting Information File 1, Figures S56–S88).

To gain quantitative insight into the complexation between the hosts and the guests, we carried out ^1^H NMR titrations and calculated the association constants *K*_a_ by the nonlinear curve-fitting method [40]. As shown in Table 1, compared with its derivatives, the unsubstituted host helic[6]arene **H1** showed the strongest complexation with all guests tested. The association constant (*K*_a_) of complex **H1·G1** was calculated to be (6.76 ± 1.02) × 10^3^ M^−1^, while the *K*_a_ of **H2·G1** was much lower (1.03 ± 0.15) × 10^2^ M^−1^). For (*O*-2-methoxyethoxy)_6_-2,6-helic[6]arene **H4** and (*O*-2-hydroxyethoxy)_6_-2,6-helic[6]arene **H5**, the association constants of their complexes with **G1** were found to be (1.36 ± 0.17) × 10^3^ M^−1^ and (3.10 ± 0.30) × 10^3^ M^−1^, respectively, which are only slightly smaller than that of **H1·G1**, but much higher than that of **H2·G1**. In the case of **H3** containing *n*-butoxy groups, almost no binding affinity toward **G1** was observed under these conditions.

**Table 1:** Association constants (*K*_a_) for 1:1 host–guest complexes in CDCl_3_/acetone-*d*_6_ 1:2 (v/v) at 298 K.

Complexes	*K_a_* [M^−1^]	Complexes	*K*_a_ [M^−1^]

**H1·G1**	(6.76 ± 1.02) × 10^3^	**H1·G3**	(1.28 ± 0.17) × 10^2^
**H2·G1**	(1.03 ± 0.15) × 10^2^	**H2·G3**	–^a^
**H3·G1**	–^a^	**H3·G3**	–^a^
**H4·G1**	(1.36 ± 0.17) × 10^3^	**H4·G3**	(73.33 ± 8.09)
**H5·G1**	(3.10 ± 0.30) × 10^3^	**H5·G3**	(88.72 ± 0.96)
**H1·G2**	(1.22 ± 0.17) × 10^4^	**H1·G4**	(7.26 ± 0.93) × 10^3^
**H2·G2**	(1.26 ± 0.16) × 10^2^	**H2·G4**	–^a^
**H3·G2**	–^a^	**H3·G4**	–^a^
**H4·G2**	(2.72 ± 0.39) × 10^3^	**H4·G4**	(2.27 ± 0.31) × 10^3^
**H5·G2**	(3.50 ± 0.48) × 10^3^	**H5·G4**	(3.04 ± 0.02) × 10^3^

^a^*K*_a_ values not calculated due to too small binding.

Compared with **G1**, the protonated 4,4'-bipyridinium salt **G3** showed similar complexation behavior but significantly lower binding abilities with helic[6]arene **H1** and its derivatives **H2**–**5**.

It is known that ion-pairing effects can hamper the complexation of charged species [41–43], and thus affect the host–guest complexation [10,44–45]. Consequently, we also prepared the 4,4'-bipyridinium salts **G2** and **G4** with BArF^−^ as the counteranion. As shown in Table 1, compared with guests **G1** and **G3** with PF_6_^−^ as the counteranion, **G2** and **G4** exhibited higher binding abilities with the hosts probably due to a weakened ion-pairing effect. Especially, for complex **H1·G2**, the *K*_a_ value was high (1.22 ± 0.17) × 10^4^ M^−1^.

Solvents with different polarity can also affect the complexation between the hosts and the guests. As shown in Table 2, we found that performing the ^1^H NMR titrations of the host–guest complexation in CDCl_3_/acetone-*d*_6_ 1:2 (v/v), the *K*_a_ values of the 1:1 host–guest complexes were about 10^3^ M^−1^ except for **H2** that showed very low complexation ability with **G4**. When the ^1^H NMR titrations were carried out in CD_2_Cl_2_, the *K*_a_ values of complexes **H1·G4**, **H3·G4** and **H4·G4** were all higher than 10^4^ M^−1^, while the *K*_a_ value of complex **H2·G4** was found to be (6.07 ± 0.08) × 10^2^ M^−1^. These results suggest that, compared with the non-polar solvent, acetone hampers or competes the intermolecular non-covalent interactions between the hosts and the guests, and thus resulted in a decrease of the host–guest complexation.

**Table 2:** Association constants (*K*_a_) for the 1:1 host–guest complexes in different solvents at 298 K.

Complexes	*K*_a_ [M^−1^]	

	in CDCl_3_/acetone-*d*_6_ 1:2 (v/v)	in CD_2_Cl_2_

**H1·G4**	(7.26 ± 0.93) × 10^3^	(2.11 ± 0.28) × 10^4^
**H2·G4**	–^a^	(6.07 ± 0.08) × 10^2^
**H4·G4**	(2.27 ± 0.31) × 10^3^	(1.13 ± 0.15) × 10^4^
**H5·G4**	(3.04 ± 0.02) × 10^3^	(1.92 ± 0.21) × 10^4^

^a^*K*_a_ value not calculated due to too small binding.

### ESIMS studies of the formation of host–guest complexes

The electrospray ionization (ESI) mass spectra also confirmed the formation of 1:1 complexes between the hosts and the guests. By using a solution of **H1** and **G1** in chloroform/acetone 1:2 (v/v), the strongest peak at *m*/*z* 540.2056 corresponding to [**H1·G1**−2PF_6_]^2+^ was found, which was in accordance with the 1:1 complex formed in solution. Similarly, the strongest peaks at *m*/*z* 582.2526, 714.8325, 672.2842, 540.2061, 582.2522, 714.8319, 672.2836, 526.1895, 700.3148, 658.2689, 526.1898, 568.2365, 700.3143, 658.2681 corresponding to [**H2·G1**−2PF_6_]^2+^, [**H4·G1**−2PF_6_]^2+^, [**H5·G1**−2PF_6_]^2+^, [**H1·G2**−2BArF]^2+^, [**H2·G2**−2BArF]^2+^, [**H4·G2**−2BArF]^2+^, [**H5·G2**−2BArF]^2+^, [**H1·G3**−2PF_6_]^2+^, [**H4·G3**−2PF_6_]^2+^, [**H5·G3**−2PF_6_]^2+^, [**H1·G4**−2BArF]^2+^, [**H2·G4**−2BArF]^2+^, [**H4·G4**−2BArF]^2+^, [**H5·G4**−2BArF]^2+^ were observed, which further confirmed the formation of the 1:1 host–guest complexes (Supporting Information File 1, Figures S41–S55).

### Host–guest complexation in the solid state

**Figure 4:**
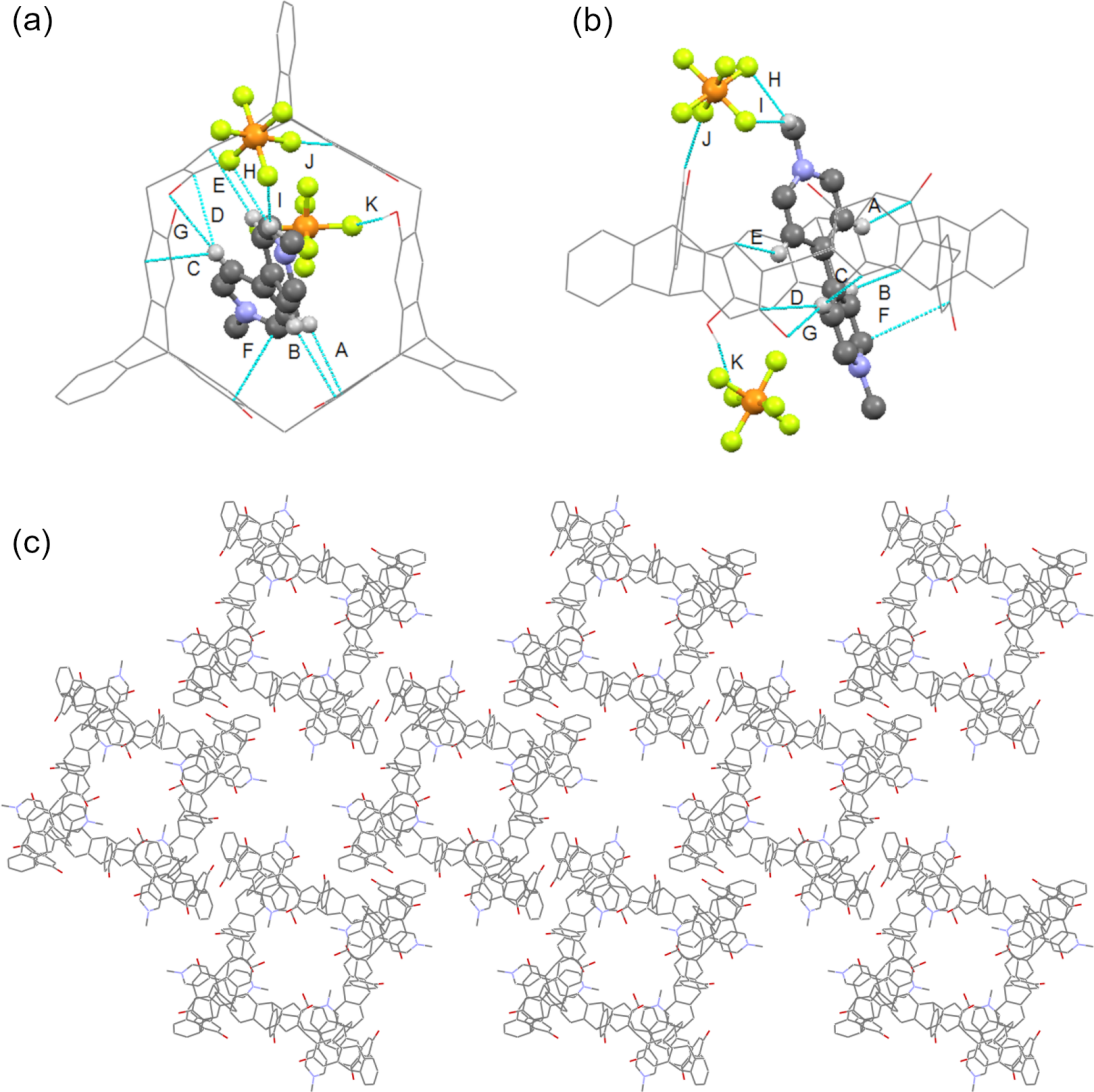
Crystal structure of complex **H1·G1**. (a) Top view, (b) side view, and (c) packing viewed along *c*-axis. Blue lines denote the non-covalent interactions between **H1** and **G1**. Solvent molecules and hydrogen atoms not involved in the non-covalent interactions were omitted for clarity.

The single crystal of complex **H1·G1** was obtained by vapor diffusion of isopropyl ether into acetone. As shown in Figure 4, **G1** was encapsulated in the cavity of **H1** to form a 1:1 complex, in which **G1** is distorted by the dihedral angle between the pyridinium rings of 33.19°. There exist multiple CH···π interactions between the protons of **G1** and the aromatic rings of **H1** with distances of 2.683 for A, 2.845 for B, 2.788 for C, 2.802 for D, and 2.868 Å for E, respectively. There also exist π–π stacking interactions between the pyridinium of **G1** and the aromatic ring of **H1** with the distance of 3.854 Å for F, a CH···O hydrogen bond between the proton of **G1** and oxygen of **H1** in the distance of 2.683 Å for G. Moreover, C-H···F hydrogen bonds between the two adjacent guests with the distances of 2.670 (H), 2.570 (I), 2.594 (J) and 1.981 Å (K), respectively, were observed. These multiple interactions play an important role in the formation of the host–guest complex. Furthermore, it was found that adjacent complexes were nearly perpendicular to each other, which self-assembled into rhombuses with hollows along the *c-*axis (Figure 4c) and curved ribbons along the *a-* and *b*-axes (Supporting Information File 1, Figure S89).

**Figure 5:**
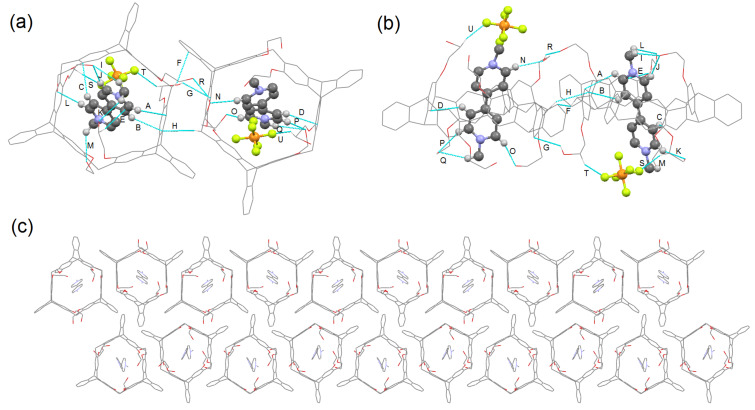
Crystal structure of complex **H5·G1**. (a) Top view, (b) side view, and (c) packing viewed along the *a-*axis. Blue lines denote the non-covalent interactions between **H5** and **G1**. Solvent molecules, PF_6_^−^ counteranions and hydrogen atoms not involved in the non-covalent interactions were omitted for clarity.

By vapor diffusion of isopropyl ether into a chloroform/acetone 1:1 (v/v) solution of the 1:1 mixture of **H3** and **G1**, we only obtained a single crystal of **H3** instead of the host–guest complex. The steric hindrance of the *n*-butoxy groups in **H3** (Supporting Information File 1, Figure S90) might lead to weak complexation of **H3** with the tested guests in solution. However, we obtained a single crystal of complex **H5·G1** by vapor diffusion of isopropyl ether into an acetone solution. As shown in Figure 5, we found that **G1** was encapsulated in the cavity of **H5** to form a 1:1 complex, and the complex molecules are stacked into infinite channels along the *a-*axis (Figure 5c), which is different from that of **H1·G1**. There exist multiple CH···π interactions between the proton of **G1** and the aromatic ring of **H5** with distances of 2.892 (A), 2.844 (B), 2.893 (C) and 2.853 Å (D), respectively. A CH···π interaction between the proton of **H5** and the aromatic ring of **G1** with a distance of 2.860 Å, and the CH···π interaction between the proton of **H5** and the aromatic ring of adjacent **H5** in the distance of 2.801 (F), 2.714 (G) and 2.887 Å (H), respectively, are also observed. Moreover, there are multiple CH···O hydrogen-bonding interactions between the protons of **G1** and the oxygen of **H5** with the distances of 2.600 (I), 2.456 (J), 2.556 (K), 2.296 (L), 2.464 (M), 2.401 (N), 2.176 (O), 2.511 (P) and 2.547 Å (Q), respectively, and OH···O hydrogen bonding between the proton of the side chain of **H5** and oxygen of the side chain of adjacent **H5** in the distance of 1.989 Å (R). In addition, C-H···F hydrogen bonds between the two adjacent guests with the distance of 2.420 (S), 2.474 (T) and 2.187 Å (U), respectively, are observed as well. These multiple intermolecular hydrogen-bonding interactions between the host and the guest might be the main reason for the formation of the stable complex **H5·G1**.

### DFT calculation of host–guest complexes

**Figure 6:**
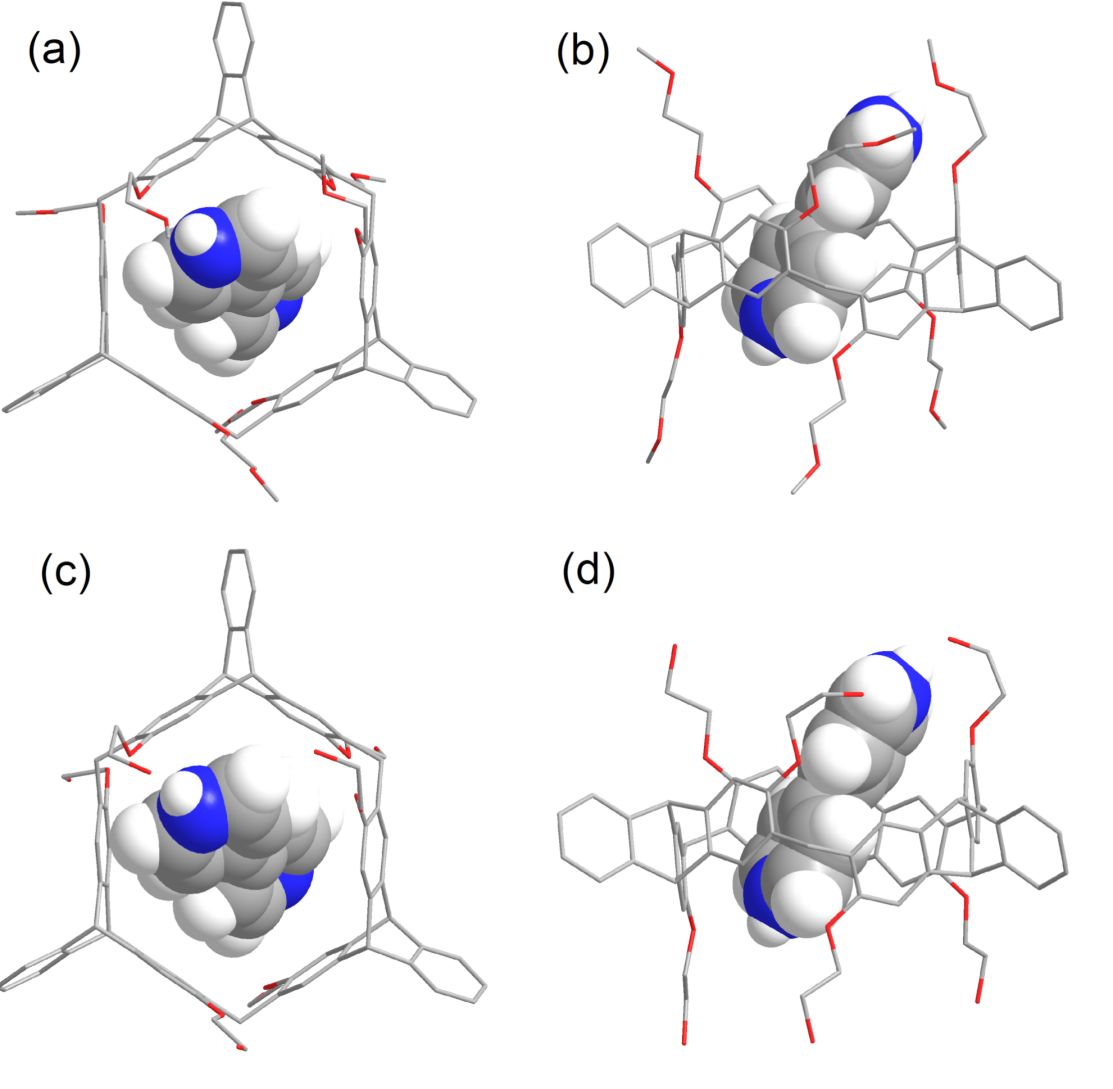
Calculated structures of the complexes at the B3LYP/6-31G level of theory. (a) Top view and (b) side view of **H4·G3**, and (c) top view and (d) side view of **H5·G3**.

To further investigate the complexation mode and structural characteristics of the host–guest complexes, DFT calculations were carried out at the B3LYP/6-31G level of theory for complex **H4·G1** (Supporting Information File 1, Figure S92). The calculation results revealed the C–H···π interactions between the protons on the pyridinium ring of **G1** and the benzene ring units of the host **H4** and C–H···O hydrogen bonds between the protons of the methyl group and pyridinium rings of **G1** and the oxygen atom of **H4** with distances ranging from 2.052 to 2.769 Å. Likewise, DFT calculations at the B3LYP/6-31G level of theory for the complexes **H4·G3** and **H5·G3** were also performed. As shown in Figure 6, in the optimized structure, the pyridinium ring of the guest is surrounded by the cavity of the host. There are C–H···π interactions between the protons on the pyridinium ring of **G3** and the benzene rings encompassing the cavity of **H4**, and C–H···O hydrogen bonding between the protons of the pyridinium ring of **G3** and the oxygen atom of **H4** with distances ranging from 2.052 to 2.769 Å. Similar to **H4·G3**, complex **H5·G3** also shows the multiple intermolecular non-covalent interactions with distances ranging from 1.651 to 2.575 Å.

Compared with hosts **H2** and **H3**, helic[6]arene **H1** and its derivatives **H4** and **H5** all show multiple hydrogen-bonding interactions with the examined guests, which were confirmed by not only X-ray crystal structures of the complexes but also by DFT calculations. These additional multiple hydrogen-bonding interactions might be responsible that **H1** and its derivatives **H4** and **H5** show stronger host–guest complexation with the tested guests than those of **H2** and **H3**. This is consistent with the results obtained in solution.

### Acid–base controlled complexation between **H2** and **G4**

**Figure 7:**
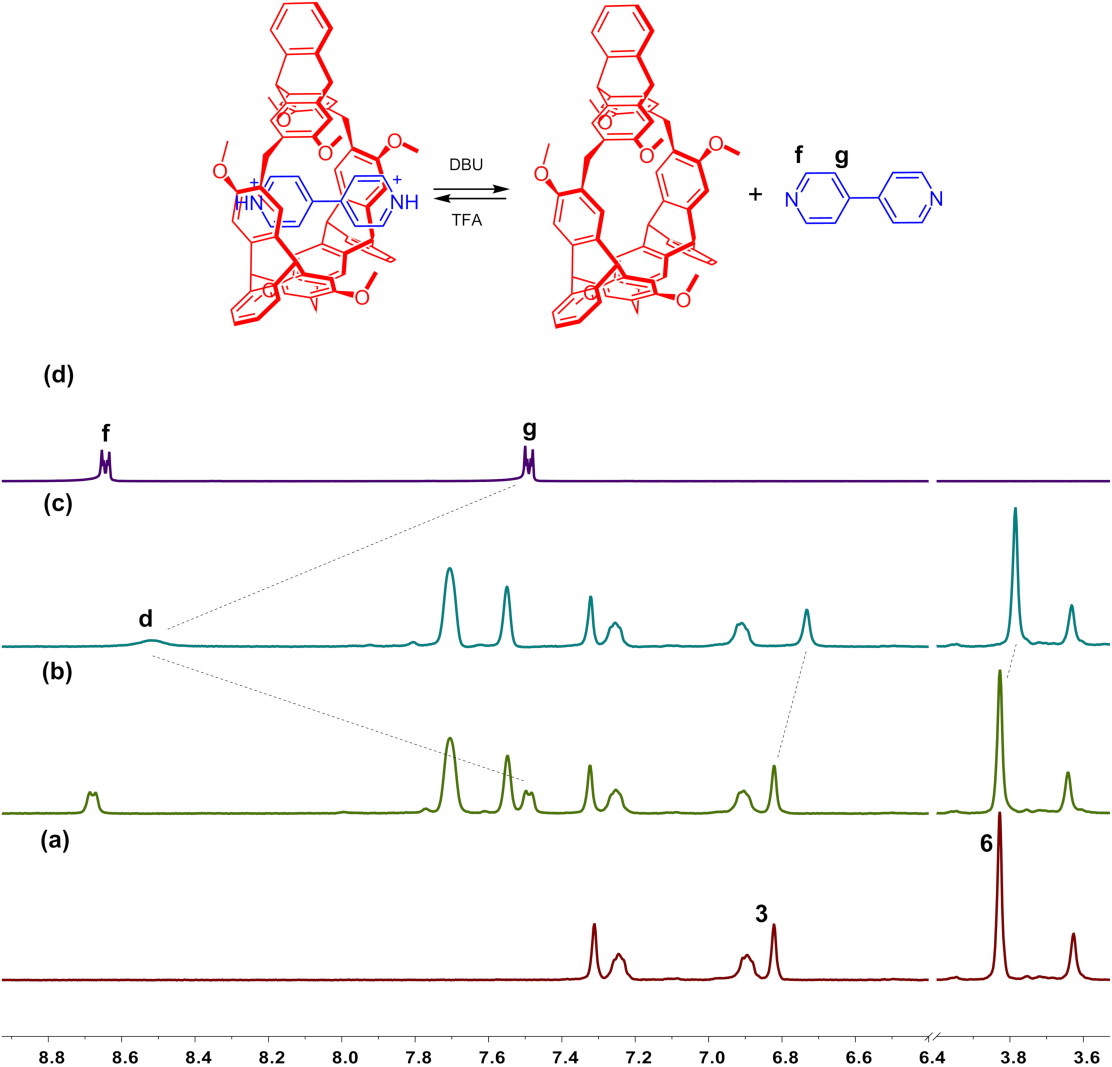
Schematic representation of the acid–base controlled complexation process and partial ^1^H NMR spectra (300 MHz, CD_2_Cl_2_, 298 K) of (a) free **H2**, (b) to the solution of complex **H2·G4** were added 2.2 equiv of DBU, (c) to the solution of b were added 2.2 equiv of TFA, and (d) free 4,4'-bipyridine. [**H2**]_0_ = 2.0 mM.

4,4′-Bipyridine easily forms protonated 4,4′-pyridinium salts and vice versa. Hence we could conveniently control the association and dissociation of the host–guest complexes based on protonated 4,4′-pyridinium guests by use of acid and base. As shown in Figure 7, when 2.2 equiv of DBU were added into the solution of complex **H2·G4** in CD_2_Cl_2_, the signals for protons 3 and 6 of complex **H2·G4** disappeared while the proton signals of free **H2** and 4,4'-bipyridine were observed, which indicated that the complex dissociated. On the other hand, when 2.2 equiv of TFA were added into the above solution, the proton signals of the free 4,4'-bipyridine and the signals for protons 3 and 6 of free **H2** disappeared, while the proton signals of complex **H2·G4** appeared again, thus indicating the regeneration of the host–guest complex. Therefore, the switchable complexation between **H2** and **G4** could be efficiently controlled by addition and removal of acid and base.

## Conclusion

In conclusion, we have demonstrated that 2,6-helic[6]arene and its derivatives could form 1:1 complexes with 1,1′-dimethyl-4,4′-bipyridinium and protonated 4,4'-bipyridinium salts in both solution and in the solid state. Compared with **H2** and **H3**, hydroxylated 2,6-helic[6]arene **H1** and its derivatives containing 2-hydroxyethoxy (**H5**) or 2-methoxyethoxy (**H4**) groups exhibited stronger complexation with the tested guests probably due to the additional multiple hydrogen-bonding interactions between the hosts and the guests, which were confirmed by X-ray single crystal structures and DFT calculations. Moreover, we also found that the association constants of the complexes could be significantly increased for the guests with a large counteranion (BArF^−^) and in non-polar solvents. Furthermore, the switchable complexation between 2,6-helic[6]arene and protonated 4,4'-bipyridinium salt could be efficiently controlled by acid and base.

## Supporting Information

File 1 Experimental, NMR spectra, mass spectra, determination of association constants, X-ray single crystal data and DFT calculation data.

File 2 CIF file for **H1**·**G1**.

File 3 CIF file for **H3**.

File 4 CIF file for **H5**·**G1**.
