# Supplementary material for: Complexation of 2,6-helic[6]arene and its derivatives with 1,1′-dimethyl-4,4′-bipyridinium salts and protonated 4,4'-bipyridinium salts: an acid–base controllable complexation
Source: Beilstein J Org Chem. 2019 Jul 26;15:1795–804. doi: 10.3762/bjoc.15.173 (PMC6664404; doi:10.3762/bjoc.15.173)
Supplement: File 1 — Experimental, NMR spectra, mass spectra, determination of association constants, X-ray single crystal data and DFT calculation data. [file Beilstein_J_Org_Chem-15-1795-s001.pdf]

BEILSTEIN JOURNAL OF ORGANIC CHEMISTRY

# Supporting Information

for

# Complexation of 2,6-helic[6]arene and its derivatives with 1,1′-dimethyl-4,4′-bipyridinium salts and protonated 4,4'-bipyridinium salts: an acid–base controllable complexation

Jing Li, Qiang Shi, Ying Han and Chuan-Feng Chen

*Beilstein J. Org. Chem.* **2019,** *15,* 1795–1804. doi:10.3762/bjoc.15.173

# Experimental, NMR spectra, mass spectra, determination of association constants, X-ray single crystal data and DFT calculation data

License and Terms: This is a supporting information file under the terms of the Creative Commons Attribution License (http://creativecommons.org/licenses/by/4.0). Please note that the reuse, redistribution and reproduction in particular requires that the authors and source are credited.
The license is subject to the *Beilstein Journal of Organic Chemistry* terms and conditions: (https://www.beilstein-journals.org/bjoc)

## Table of contents

1. General information ........................................................................................ S1

2. Synthesis of hosts and guest .................................................................................. S2

3. NMR Spectra of new compounds ......................................................................... S5

4. $^1$H NMR studies on the complexation between hosts and guests ...................... S10

5. High-resolution mass spectra for the complexes ............................................... S26

6. Nonlinear curve fitting and job plots for the complexes.................................... S33

7. Crystal structures and crystal data ..................................................................... S50

8. DFT calculations for complexation between hosts and guests .......................... S55

## 1.General information

All the reagents were commercially available and used without further purification. Reactions were carried out under inert and anhydrous conditions unless otherwise noted. Flash column chromatography was performed on 200–300 mesh silica gel. $^1$H and $^{13}$C NMR spectra were measured at 298 K. The ionization methods used in mass spectrometry were atmospheric pressure chemical ionization (APCI) and electrospray ionization (ESI). Melting points, were taken on an electrothermal melting point apparatus, and are uncorrected.

## 2. Synthesis of hosts and guest

### Synthesis of H3

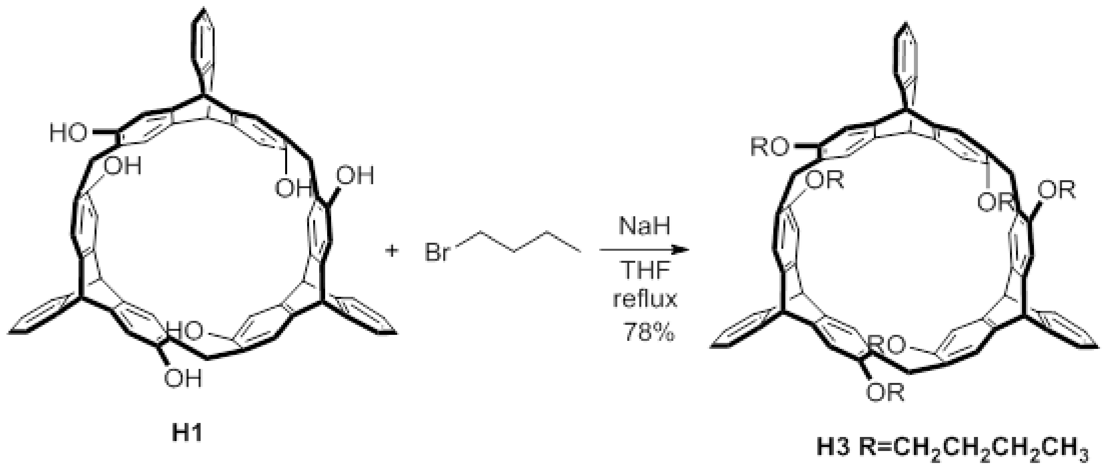


To the solution of **H1** (100 mg, 0.1 mmol) in THF (20 mL) was added sodium hydride (48 mg, 1.2 mmol) and the mixture was stirred at 66 °C for half an hour. Bromobutane (129 μL, 1.2 mmol) was added and the mixture stirred for another 10 h at 66 °C and then quenched with water. The organic layer was separated, washed with brine three times, and dried with anhydrous $MgSO_4$. The organic layer was evaporated and then purified by column chromatography on silica gel (petroleum ether/dichloromethane, 1:1, v/v) to afford **H3** (107 mg, 78% yield) as white solid. M. p.>300°C. $^1$H NMR (400 MHz, $CD_2Cl_2$) δ 7.38 (s, 6H), 7.28-7.26 (m, 6H), 6.93-6.91 (m, 6H), 6.79 (s, 6H), 5.03 (s, 6H), 4.10-3.98 (m, 6H), 3.93-3.81 (m, 6H), 3.65 (s, 6H), 1.97-1.92 (m, 12H), 1.70-1.64 (m, 12H), 1.13 (t, $J$ = 7.4 Hz, 18H). $^{13}$C NMR (100 MHz, $CD_2Cl_2$) δ 153.5, 146.4, 144.4, 136.7, 126.2, 125.6, 124.6, 122.8, 107.6, 68.1, 31.8, 19.6, 13.9. HRMS (APCI) *m/z*: $[M+H]^+$ calcd for $C_{87}H_{91}O_6$ 1231.6810; found 1231.6798.

**Synthesis of H4.**

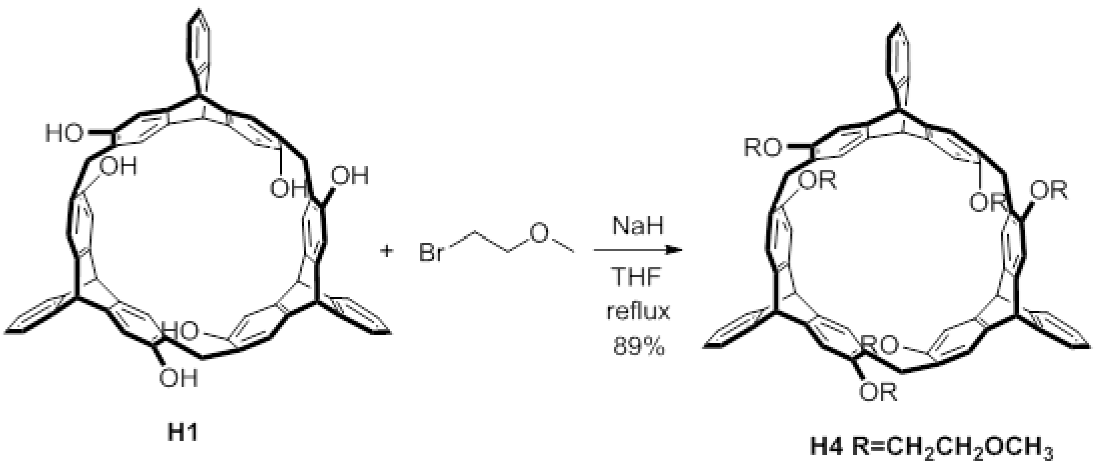


To the solution of **H1** (100 mg, 0.1 mmol) in THF (20 mL) was added sodium hydride (48 mg, 1.2 mmol) and the mixture was stirred at 66 °C for half an hour. 2-Bromoethyl methyl ether (113 μL, 1.2 mmol) was added and the mixture stirred for another 10 h at 66 °C and then quenched with water. The organic layer was separated, washed with brine three times, and dried with anhydrous $MgSO_4$. The organic layer was evaporated and then crystallized from diethyl ether to afford **H4** (124 mg, 89% yield) as white solid. M. p. 252-256°C. $^1$H NMR (400 MHz, $CD_2Cl_2$) δ 7.45 (s, 6H), 7.28-7.25 (m, 6H), 6.93-6.91 (m, 6H), 6.78 (s, 6H), 5.06 (s, 6H), 4.15-4.11 (m, 6H), 4.06-4.01 (m, 6H), 3.92–3.83 (m, 12H), 3.68 (s, 6H), 3.58 (s, 18H). $^{13}$C NMR (100 MHz, $CD_2Cl_2$) δ 153.08, 146.43, 144.35, 137.31, 126.74, 125.81, 124.63, 122.89, 107.93, 71.44, 67.61, 58.86. HRMS (APCI) *m/z*: $[M+H]^+$ calcd for $C_{81}H_{79}O_{12}$ 1243.5566; found 1243.5554.

**Synthesis of H5**

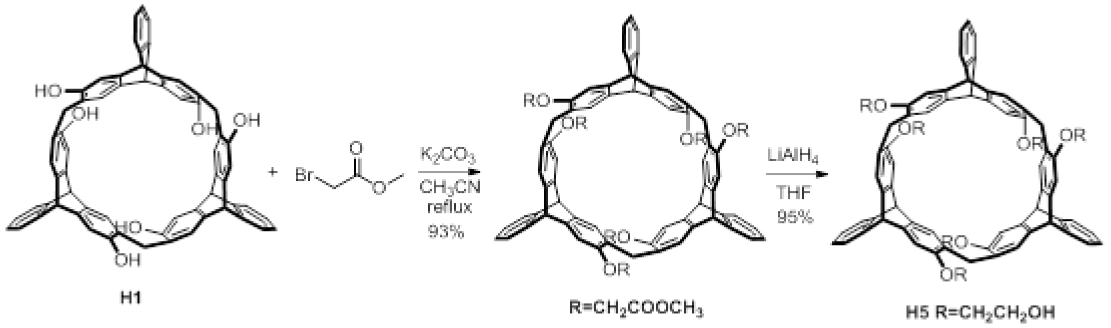


To the solution of **H1** (100 mg, 0.1 mmol) in $CH_3CN$ (20 mL) was added $K_2CO_3$ (462 mg, 3.3 mmol) and the mixture was stirred at 80 °C for half an hour. Bromoacetate (504 mg, 3.3 mmol) was added and the mixture stirred for another 10 h at 80 °C and then quenched with water. The organic layer was separated, washed with brine three times, and dried with anhydrous $MgSO_4$. The organic layer was evaporated and then crystallized from diethyl ether to afford methoxycarbonyl-substituted 2,6-helic[6]arene (138 mg, 93% yield) as white solid. Then, the obtained white solid (138 mg, 0.1 mmol) was dissolved in anhydrous THF (20 mL), $LiAlH_4$ (46 mg, 1.2 mmol) was added to the solution at 0 °C, stirred at 25 °C for 12 h, and then heated under reflux for 4 h. After cooling, 1.0 M of aqueous NaOH was added dropwise until gas evolution ceased. The resulting mixture was filtered and concentrated in vacuo to give a white solid as crude product, which was further washed with water by sonication to afford pure **H5** (115 mg, 95% yield) as white solid. M. p. >300°C. $^1$H NMR (500 MHz, acetone-$d_6$) δ 7.45 (s, 6H), 7.16-7.15 (m, 6H), 6.76-6.75 (m, 6H), 6.71 (s, 6H), 5.09 (s, 6H), 3.94-3.90 (m, 24H), 3.55 (s, 6H). $^{13}$C NMR (125 MHz, acetone-$d_6$) δ 153.3, 146.7, 144.9, 137.4, 126.2, 125.77, 124.4, 122.9, 107.8, 69.9, 60.9, 53.0. HRMS (ESI) *m/z*: $[M+Na]^+$ calcd for $C_{75}H_{66}O_{12}Na$ 1181.4447; found 1181.4434.

**Synthesis of G4**

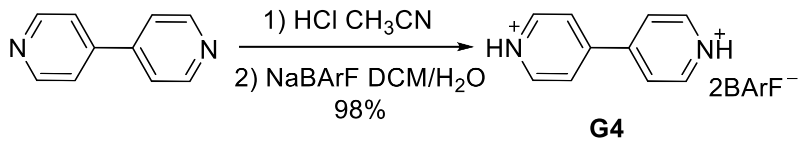


To a stirred solution of 4,4'-bipyridine (100 mg, 0.6 mmol) in $CH_3CN$ (10 mL) was added excess HCl, the reaction mixture was stirred for 12 h followed by filtration. The residue was washed with $CH_3CN$ for three times and then dissolved in DCM/$H_2O$ 1:1 (v/v, 10 mL) and treated with NaBArF (1135 mg, 1.2 mmol). The solution was stirred at ambient temperature for 6 h and transferred to a separatory funnel. The DCM solution was collected and concentrated in vacuo to afford pure **G4** (1.18 g, 98% yield) as white solid. M. p. 246-248°C. $^1$H NMR (500 MHz, acetone-$d_6$) δ 9.58 (d, J = 6.2 Hz, 4H), 9.00 (d, J = 6.2 Hz, 4H), 7.80 (s, 16H), 7.69 (s, 8H). $^{13}$C NMR (125 MHz, acetone-$d_6$) δ 162.3, 161.9, 161.5, 161.1, 152.6, 143.6, 134.6, 129.5, 129.3, 129.2, 129.2, 129.2, 129.0, 129.0, 129.0, 128.9, 128.7, 128.7, 127.7, 127.0, 125.5, 123.4, 121.2, 117.6, 117.6, 117.5, 117.5, 117.5. HRMS (ESI) *m/z*: [M-2BArF]$^{2+}$ calcd for $C_{10}H_{10}N_2$ 79.0417; found 79.0434.

## 3. NMR Spectra of new compounds

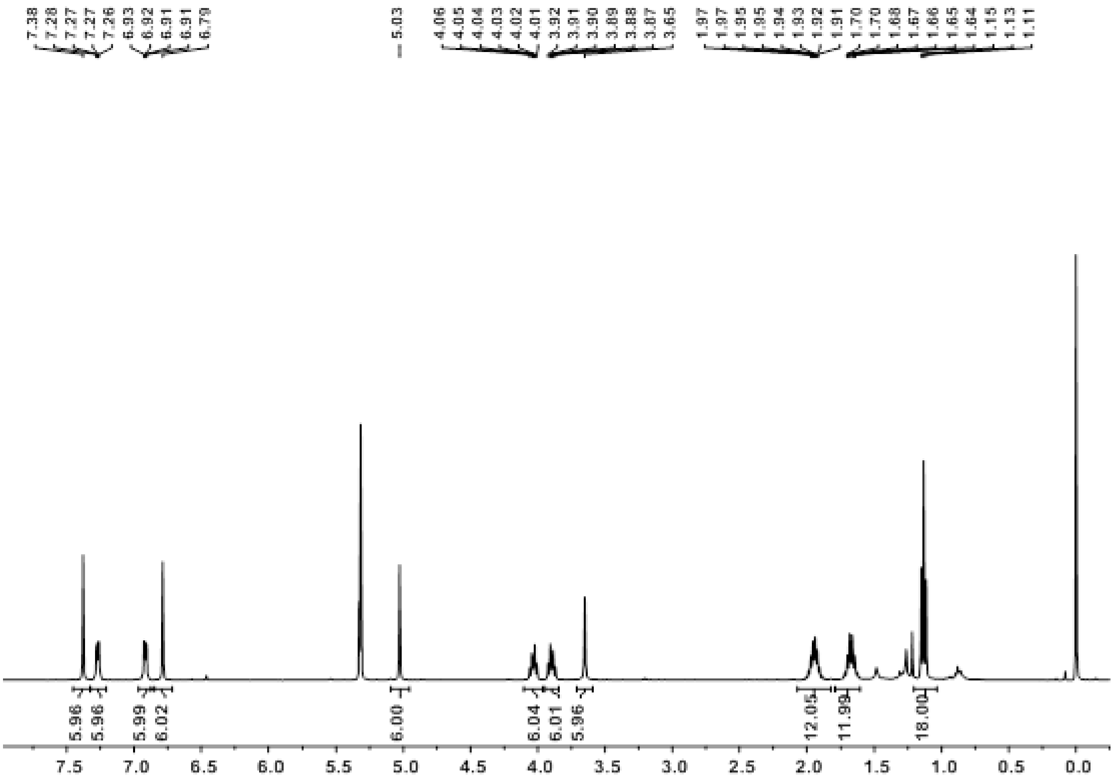


**Figure S1:** $^{1}$H NMR spectrum (400 MHz, $CD_2Cl_2$, 298 K) of **H3**.

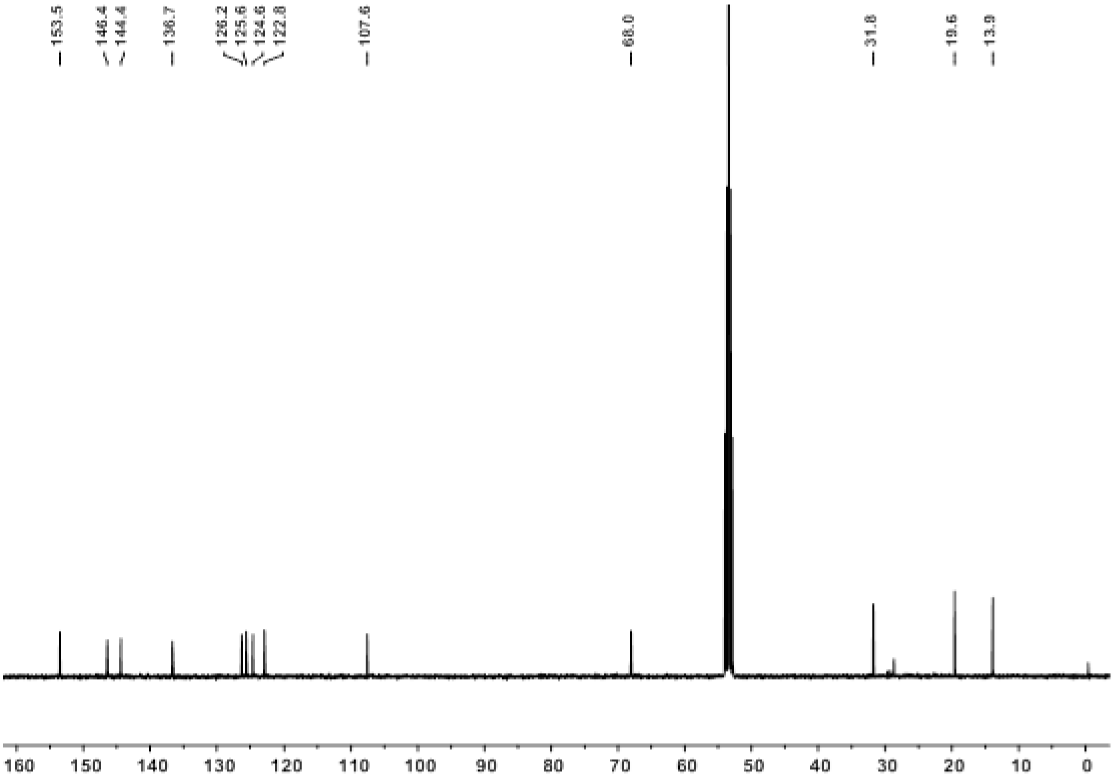


**Figure S2:** $^{13}$C NMR spectrum (100 MHz, $CD_2Cl_2$, 298 K) of **H3**.

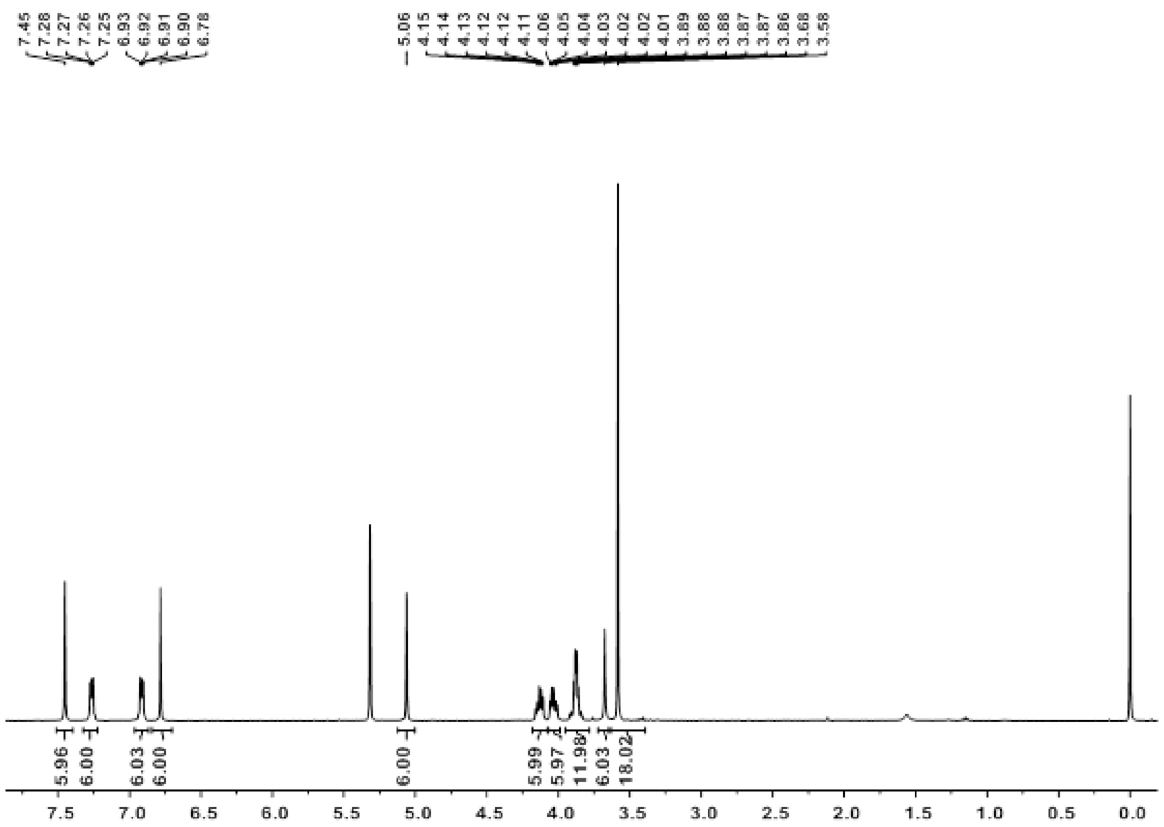


**Figure S3:** $^{1}H$ NMR spectrum (400 MHz, $CD_2Cl_2$, 298 K) of **H4**.

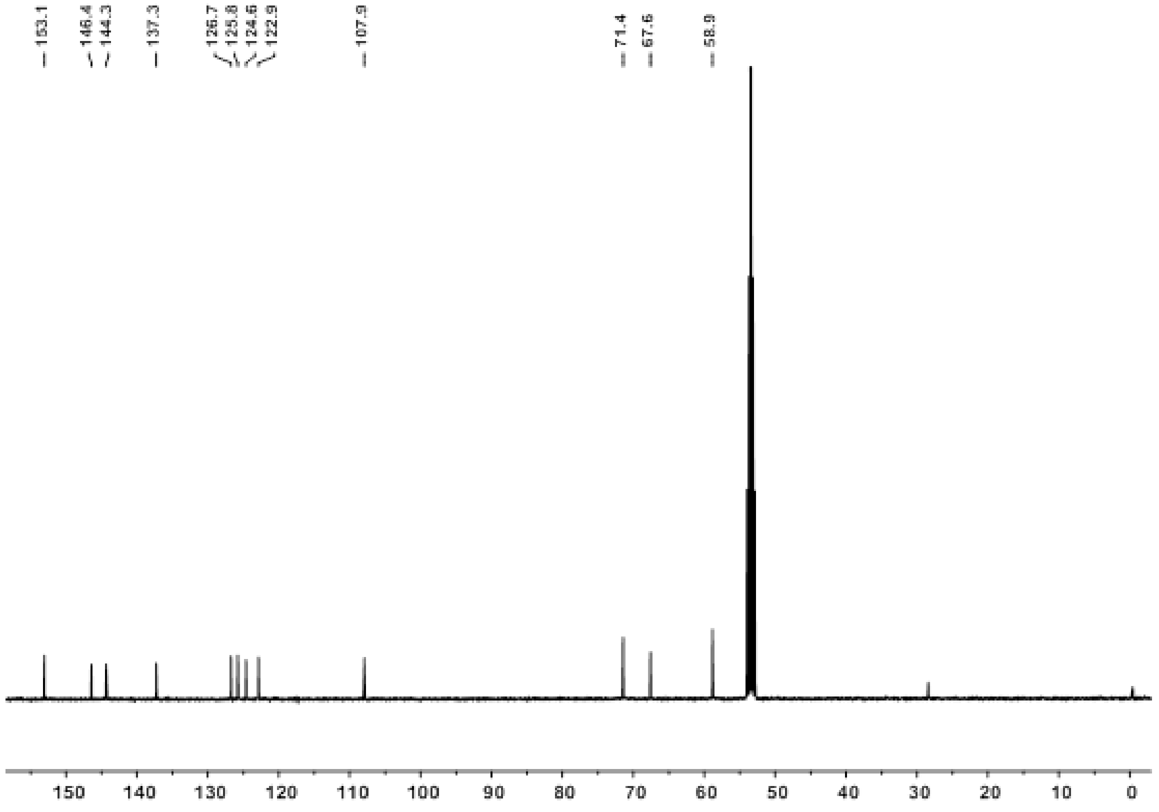


**Figure S4**: $^{13}C$ NMR spectrum (100 MHz, $CD_2Cl_2$, 298 K) of **H4**.

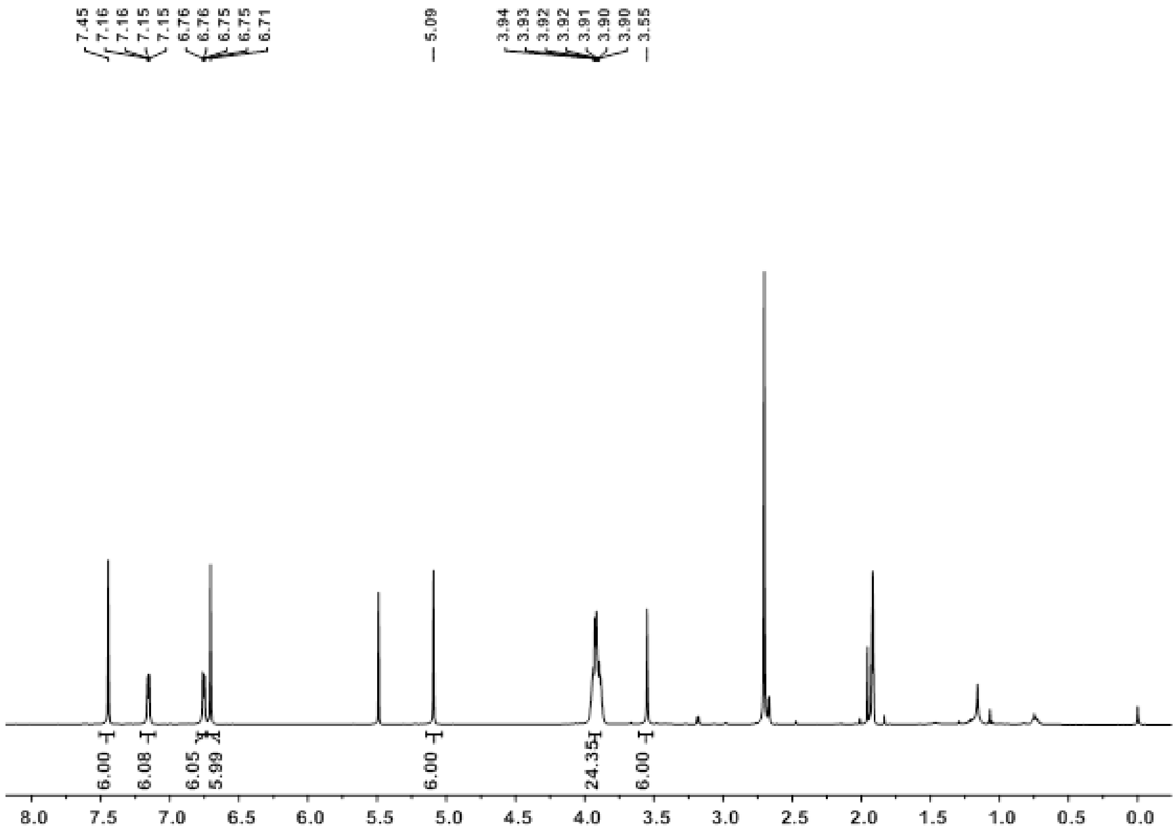


**Figure S5:** $^{1}$H NMR spectrum (500 MHz, acetone-$d_6$, 298 K) of **H5**.

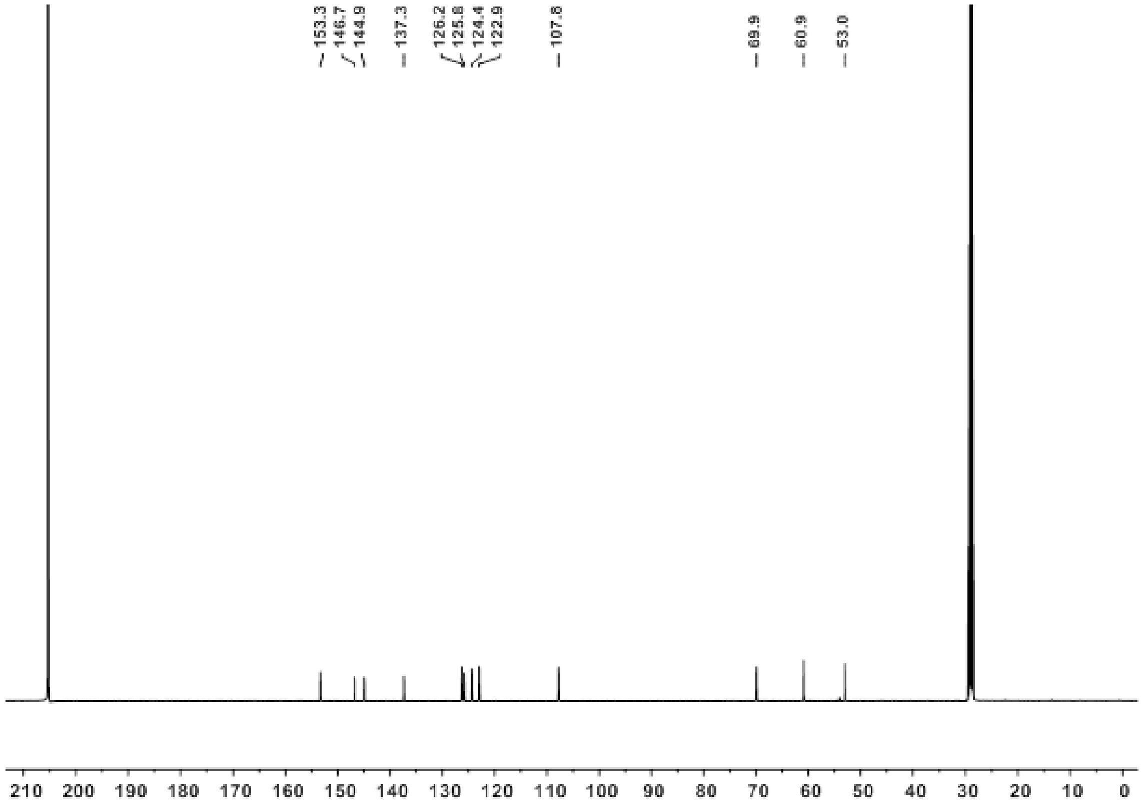


**Figure S6:** $^{13}$C NMR spectrum (125 MHz, acetone-$d_6$, 298 K) of **H5**.

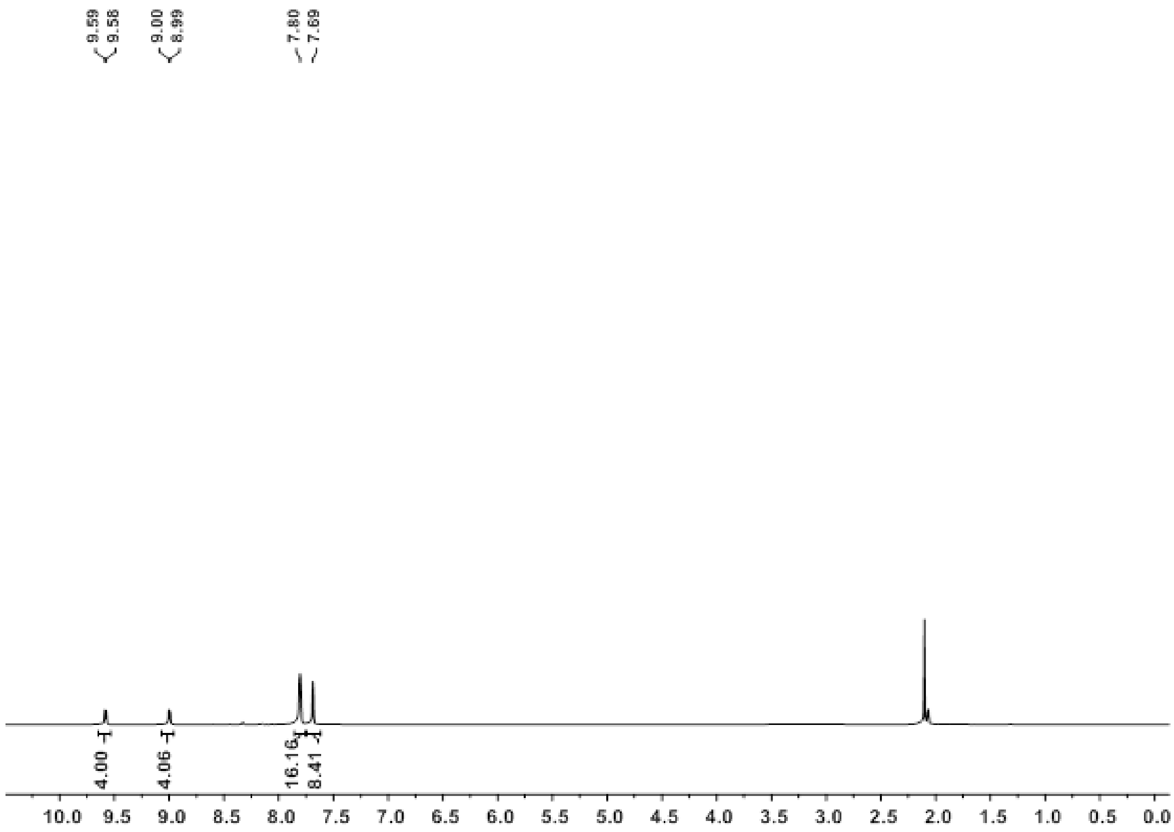


**Figure S7:** $^{1}$H NMR spectrum (500 MHz, acetone-$d_6$, 298 K) of **G4**.

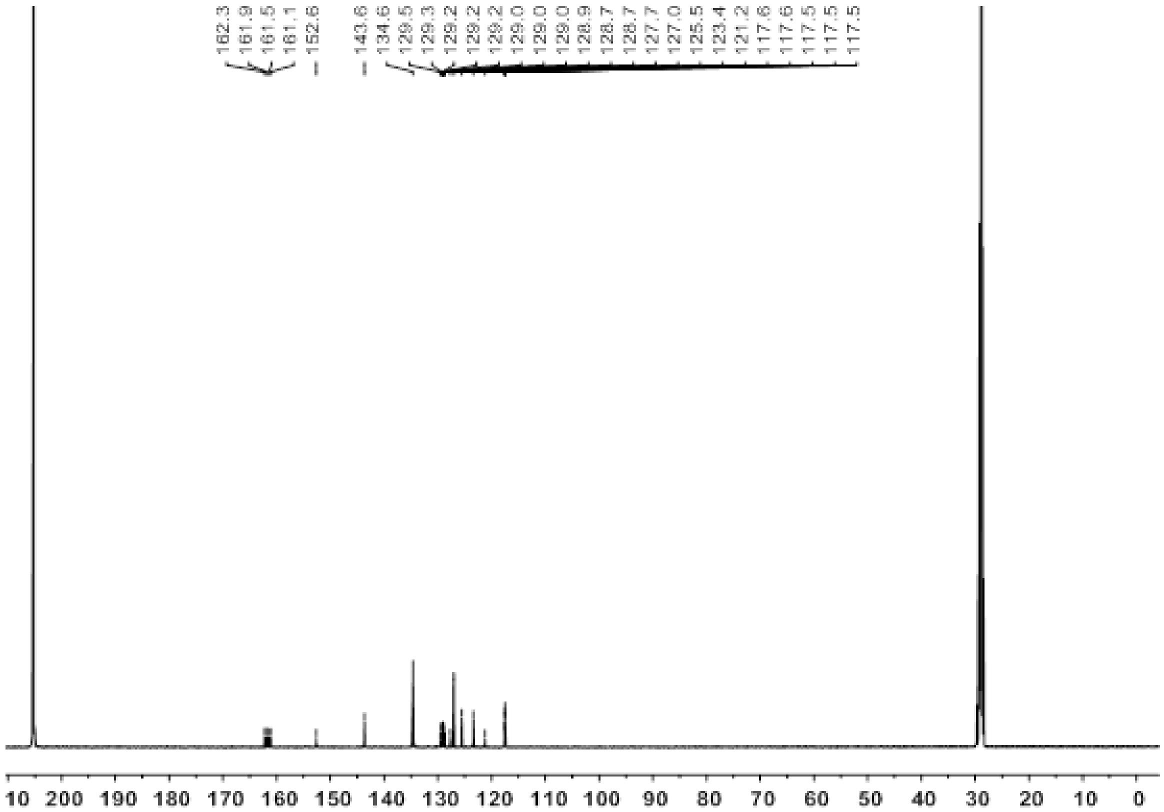


**Figure S8:** $^{13}$C NMR spectrum (125 MHz, acetone-$d_6$, 298 K) of **G4**.

## 4. $^{1}$HNMR studies on the complexation between hosts and guests

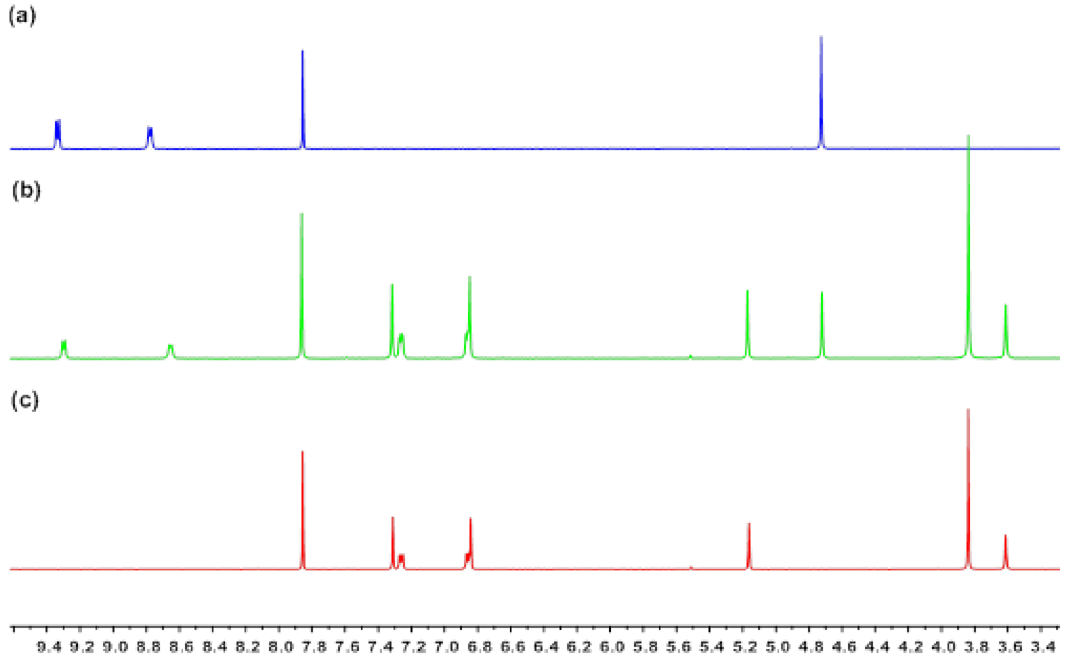


**Figure S9:** Partial $^{1}$H NMR spectra (400 MHz, 298 K, $CDCl_3$/acetone-$d_6$ 1:2 (v/v)) of (a) **G1**, (b) **H2** + 1.0 equiv **G1**, (c) **H2**. $[\textbf{H2}]_0$ = 2.00 mM

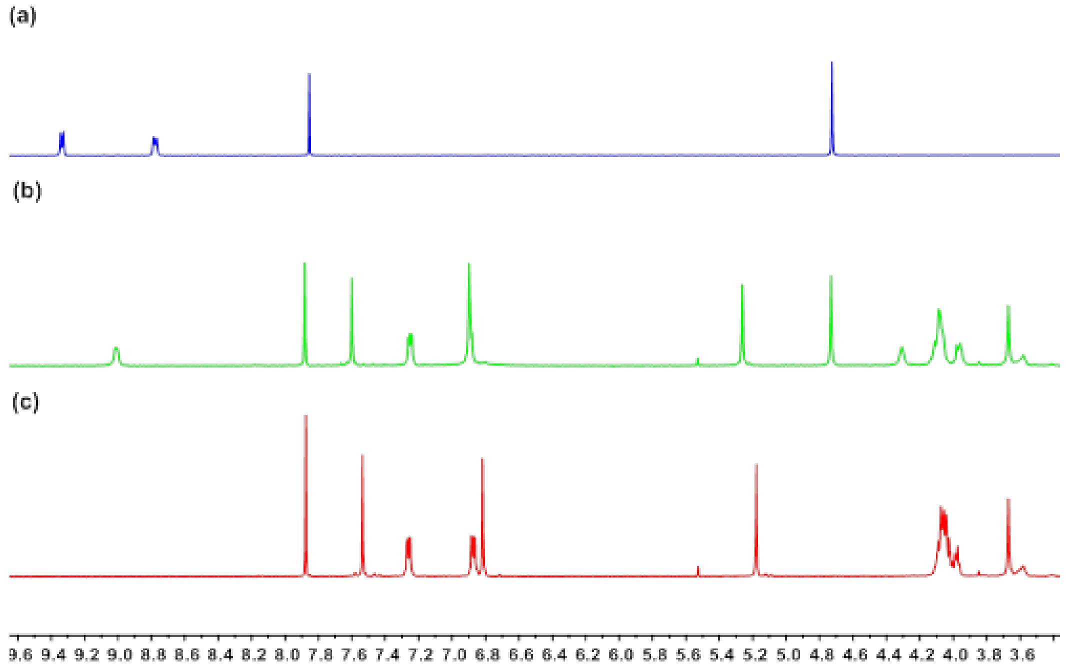


**Figure S10:** Partial $^{1}$H NMR spectra (400 MHz, 298 K, $CDCl_3$/acetone-$d_6$ 1:2 (v/v)) of (a) **G1**, (b) **H5** + 1.0 equiv **G1**, (c) **H5**. $[\textbf{H5}]_0$ = 2.00 mM.

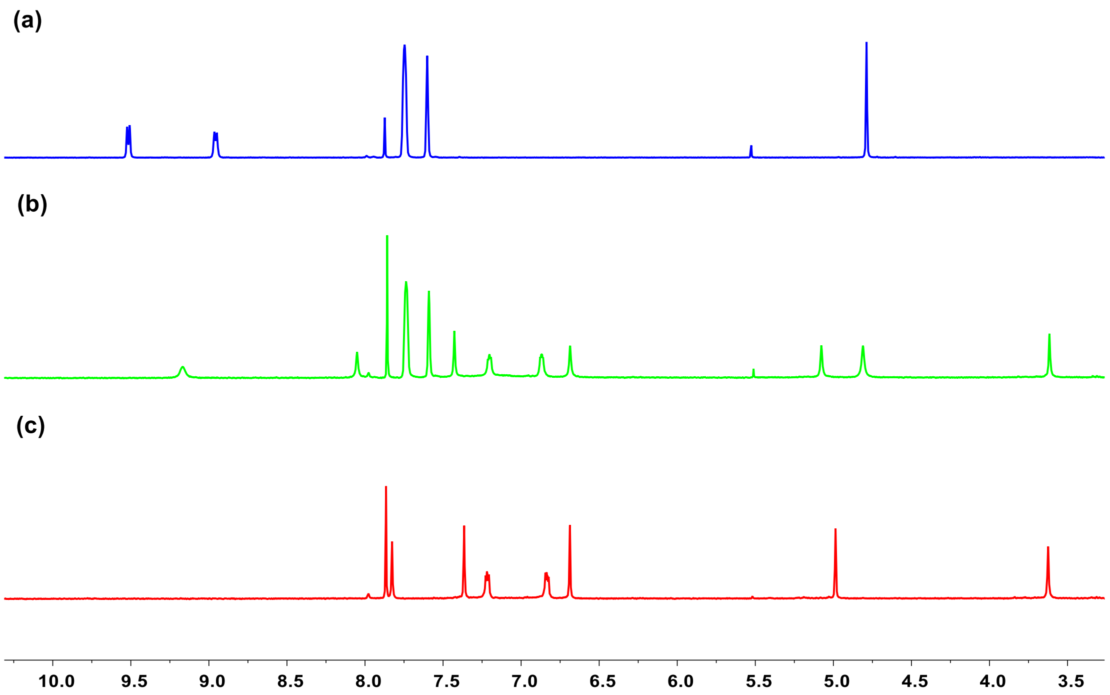


**Figure S11:** Partial $^{1}$H NMR spectra (400 MHz, 298 K, $CDCl_3$/acetone-$d_6$ 1:2 (v/v)) of (a) **G2**, (b) **H1** + 1.0 equiv **G2**, (c) **H1**. [**H1**]$_0$ = 2.00 mM.

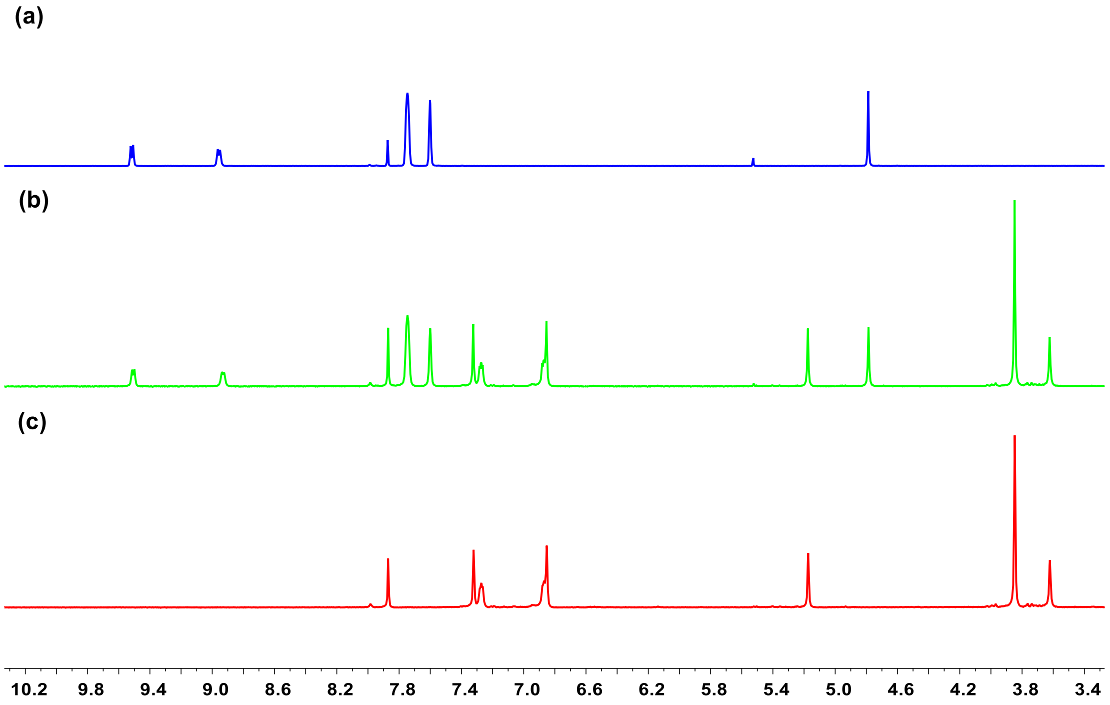


**Figure S12:** Partial $^{1}$H NMR spectra (400 MHz, 298 K, $CDCl_3$/acetone-$d_6$ 1:2 (v/v)) of (a) **G2**, (b) **H2** + 1.0 equiv **G2**, (c) **H2**. [**H2**]$_0$ = 2.00 mM.

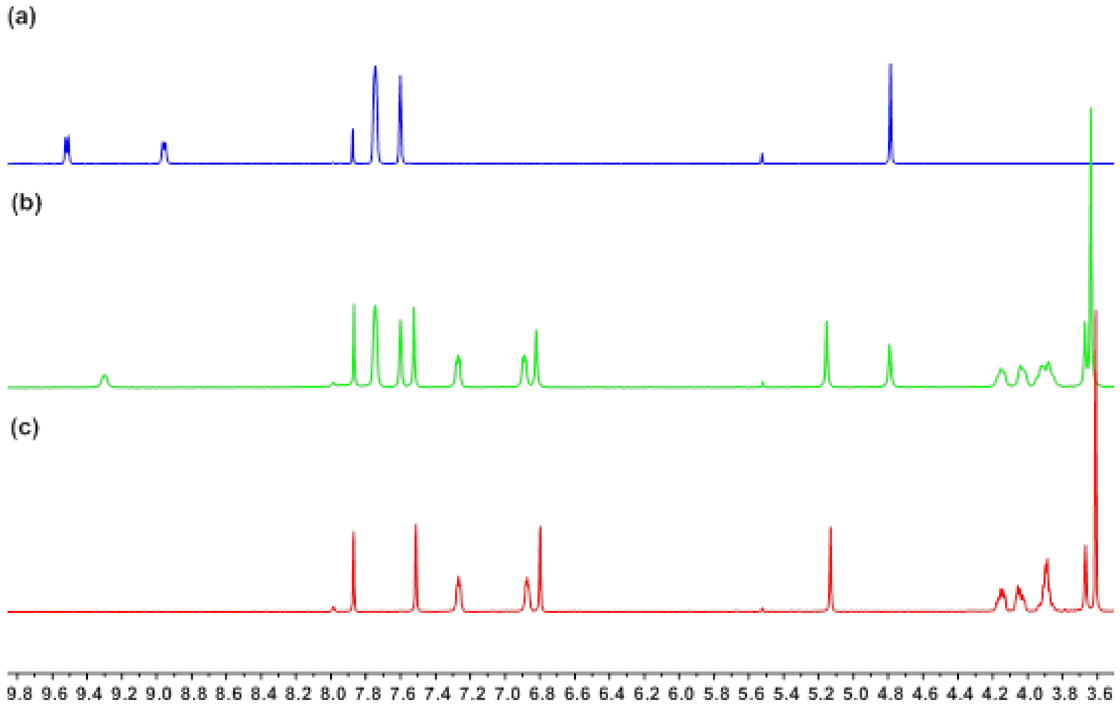


**Figure S13:** Partial $^{1}$H NMR spectra (400 MHz, 298 K, $CDCl_3$/acetone-$d_6$ 1:2 (v/v)) of (a) **G2**, (b) **H4** + 1.0 equiv **G2**, (c) **H4**. [**H4**]$_0$ = 2.00 mM.

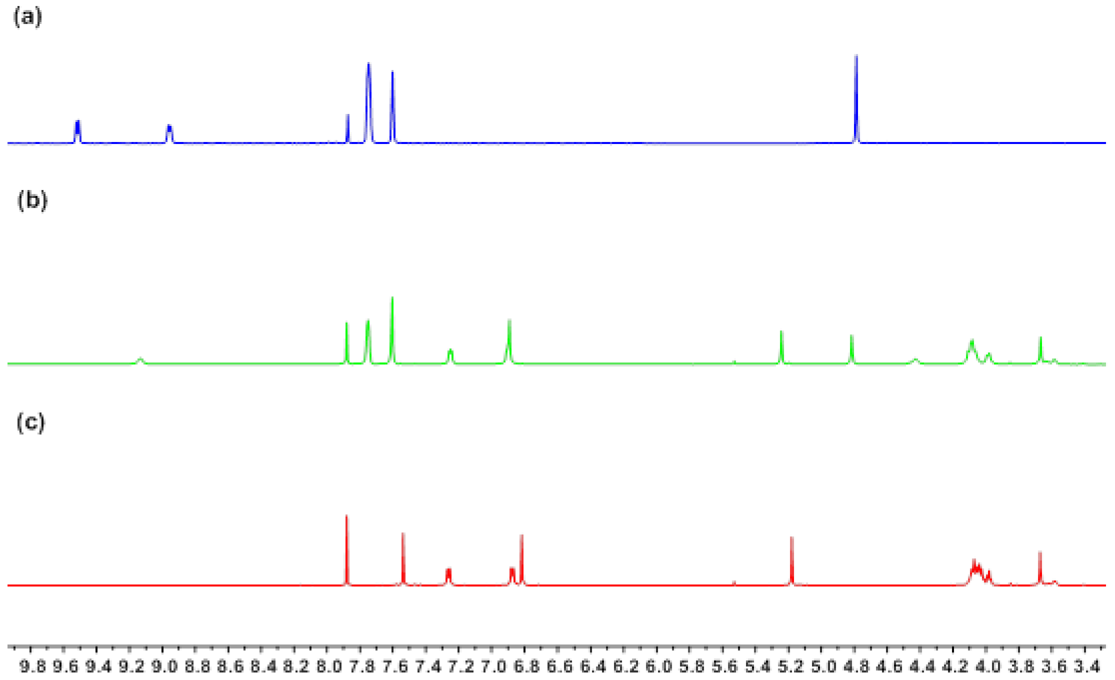


**Figure S14:** Partial $^{1}$H NMR spectra (500 MHz, 298 K, $CDCl_3$/acetone-$d_6$ 1:2 (v/v)) of (a) **G2**, (b) **H5** + 1.0 equiv **G2**, (c) **H5**. [**H5**]$_0$ = 2.00 mM.

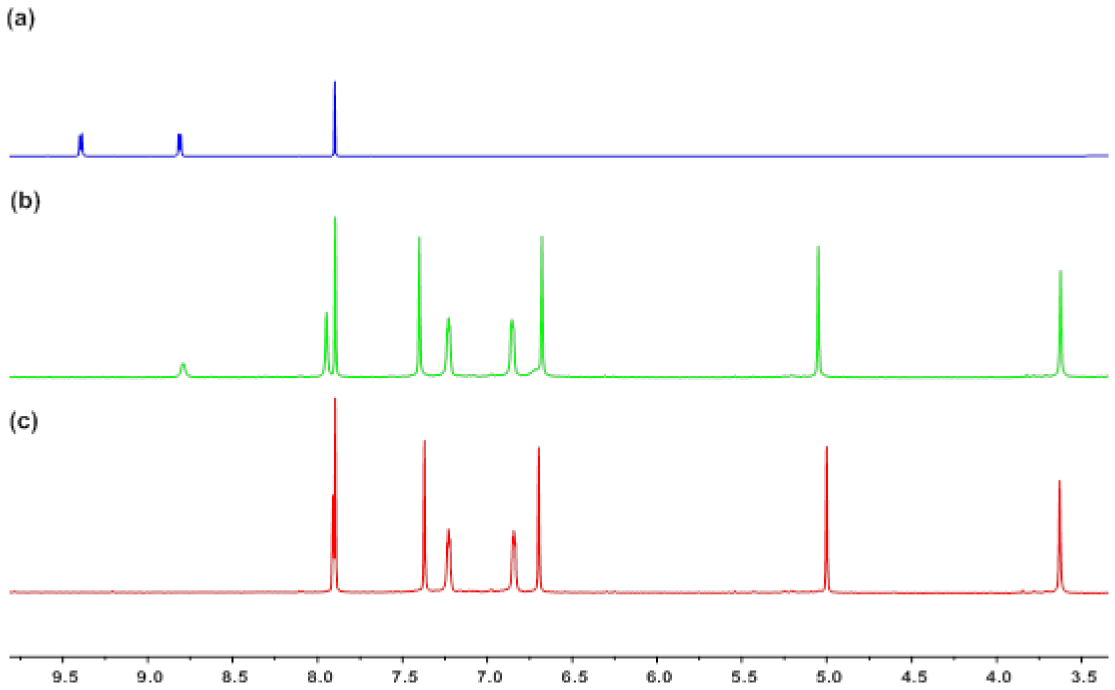


**Figure S15:** Partial $^{1}$H NMR spectra (500 MHz, 298 K, $CDCl_3$/acetone-$d_6$ 1:2 (v/v)) of (a) **G3**, (b) **H1** + 1.0 equiv **G3**, (c) **H1**. [**H1**]$_0$ = 2.00 mM.

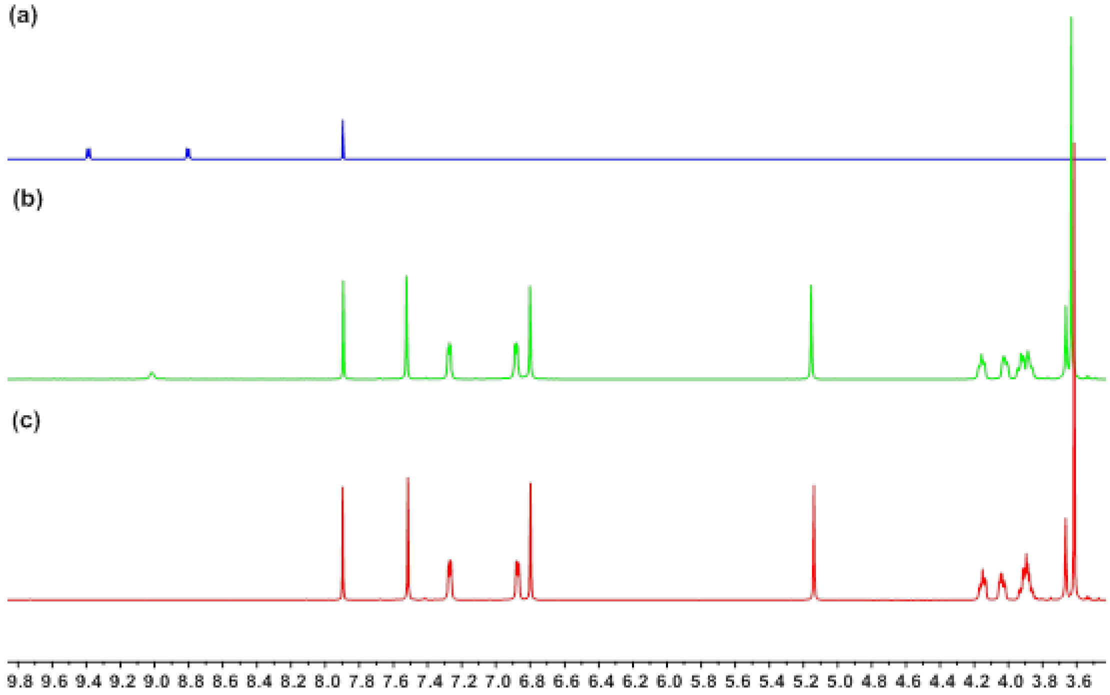


**Figure S16:** Partial $^{1}$H NMR spectra (500 MHz, 298 K, $CDCl_3$/acetone-$d_6$ 1:2 (v/v)) of (a) **G3**, (b) **H4** + 1.0 equiv **G3**, (c) **H4**. [**H4**]$_0$ = 2.00 mM.

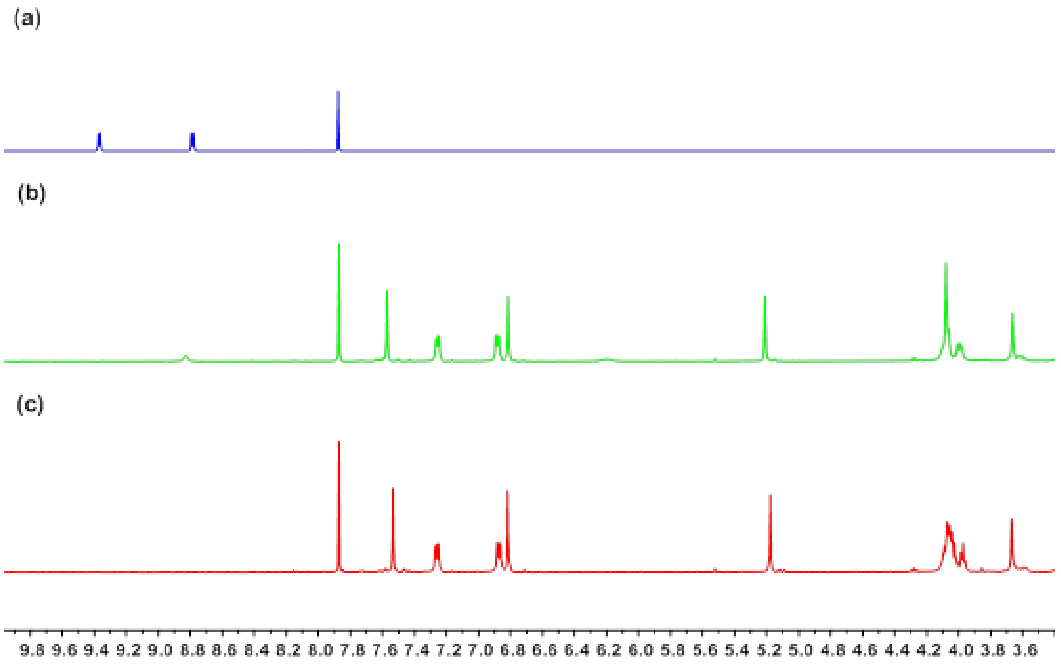


**Figure S17:** Partial $^{1}$H NMR spectra (400 MHz, 298 K, $CDCl_3$/acetone-$d_6$ 1:2 (v/v)) of (a) **G3**, (b) **H5** + 1.0 equiv **G3**, (c) **H5**. [**H5**]$_0$ = 2.00 mM.

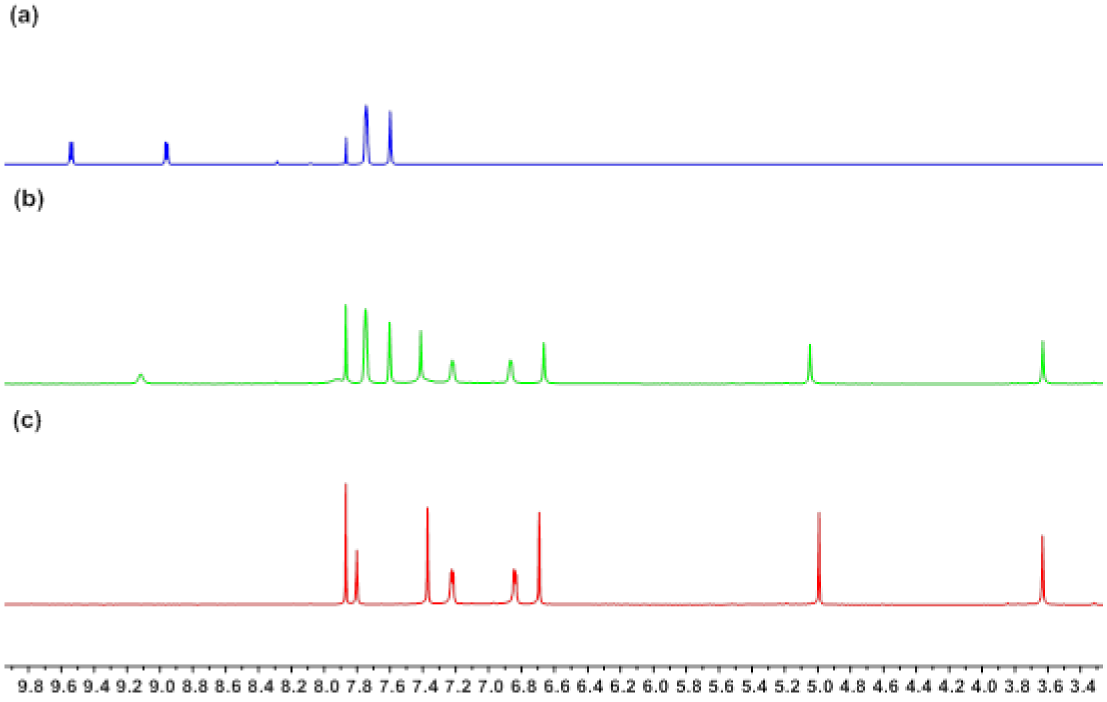


**Figure S18:** Partial $^{1}$H NMR spectra (500 MHz, 298 K, $CDCl_3$/acetone-$d_6$ 1:2 (v/v)) of (a) **G4**, (b) **H1** + 1.0 equiv **G4**, (c) **H1**. [**H1**]$_0$ = 2.00 mM.

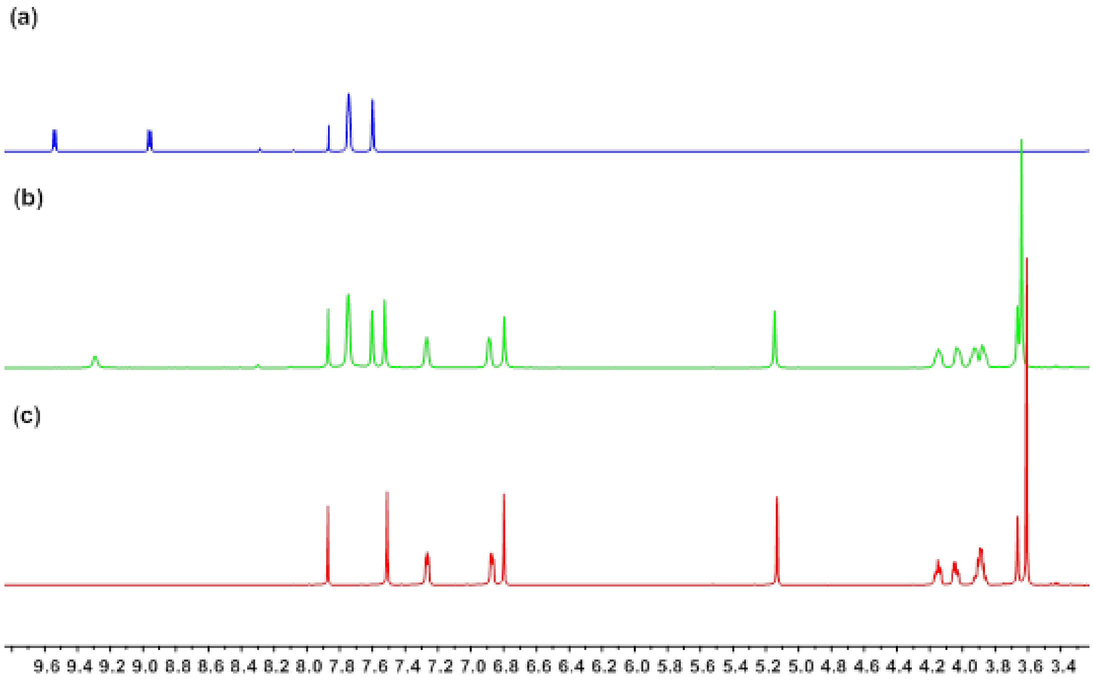


**Figure S19:** Partial $^{1}H$ NMR spectra (500 MHz, 298 K, $CDCl_3$/acetone-$d_6$ 1:2 (v/v)) of (a) **G4**, (b) **H4** + 1.0 equiv **G4**, (c) **H4**. $[\textbf{H4}]_0$ = 2.00 mM.

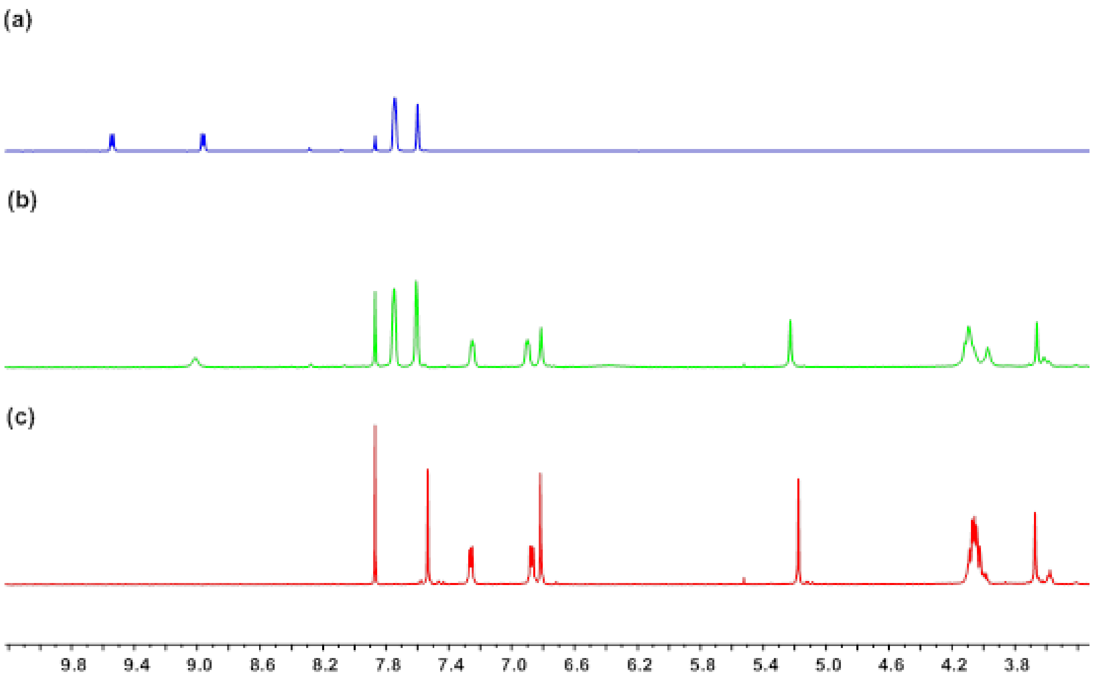


**Figure S20:** Partial $^{1}H$ NMR spectra (400 MHz, 298 K, $CDCl_3$/acetone-$d_6$ 1:2 (v/v)) of (a) **G4**, (b) **H5** + 1.0 equiv **G4**, (c) **H5**. $[\textbf{H5}]_0$ = 2.00 mM.

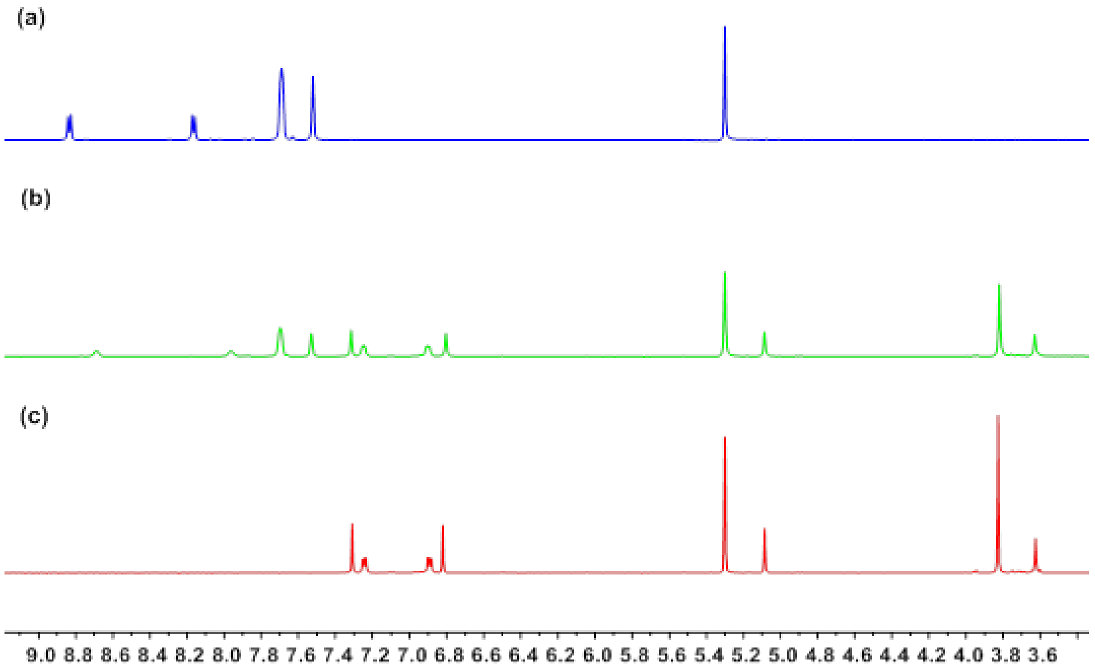


**Figure S21:** Partial $^{1}$H NMR spectra (400 MHz, 298 K, $CD_2Cl_2$) of (a) **G4**, (b) **H2** + 1.0 equiv **G4**, (c) **H2**. [**H2**]$_0$ = 2.00 mM.

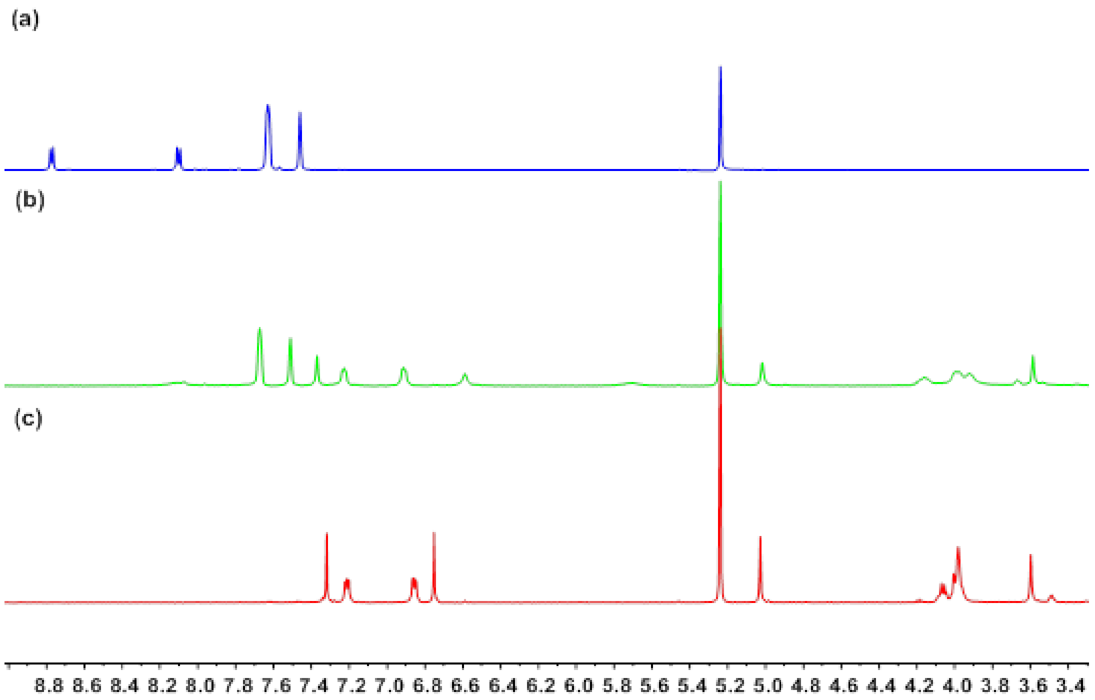


**Figure S22:** Partial $^{1}$H NMR spectra (400 MHz, 298 K, $CD_2Cl_2$) of (a) **G4**, (b) **H5** + 1.0 equiv **G4**, (c) **H5**. [**H5**]$_0$ = 2.00 mM.

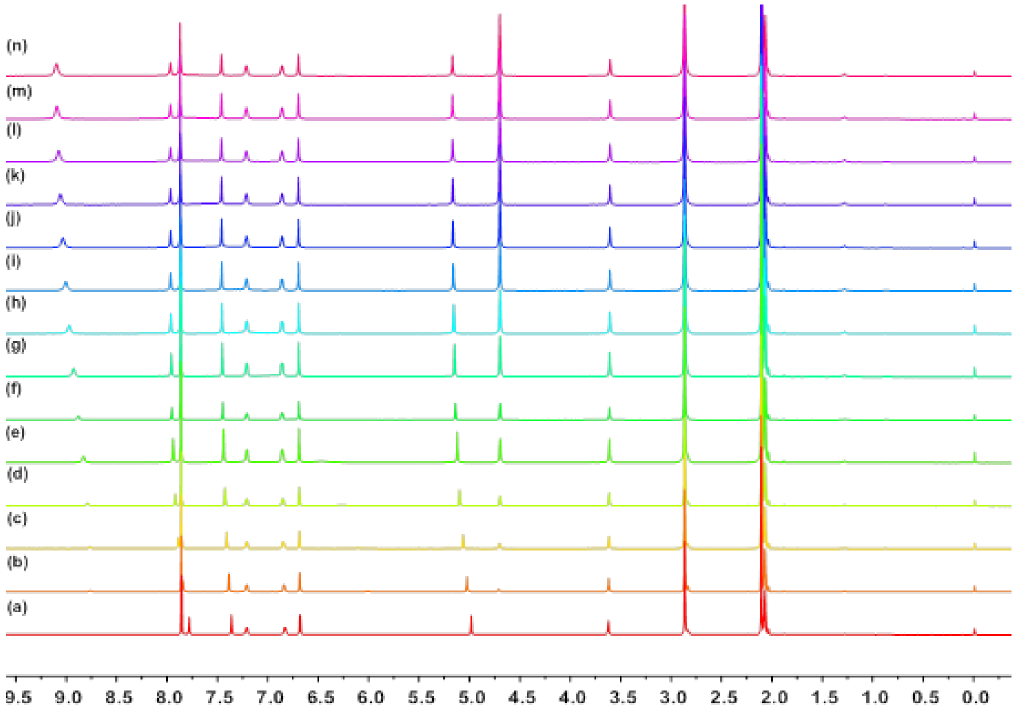


**Figure S23:** $^{1}$H NMR spectra (500 MHz, 298 K, $CDCl_3$/acetone-$d_6$ 1:2 (v/v)) of **H1** with different equivalents of **G1**: (a) 0.00, (b) 0.20, (c) 0.40, (d) 0.60, (e) 0.80, (f) 1.00, (g) 1.20, (h) 1.40, (i)1.60, (j) 1.80, (k) 2.00, (l) 2.20, (m) 2.40, (n) 2.50. [**H1**]$_0$ = 2.00 mM.

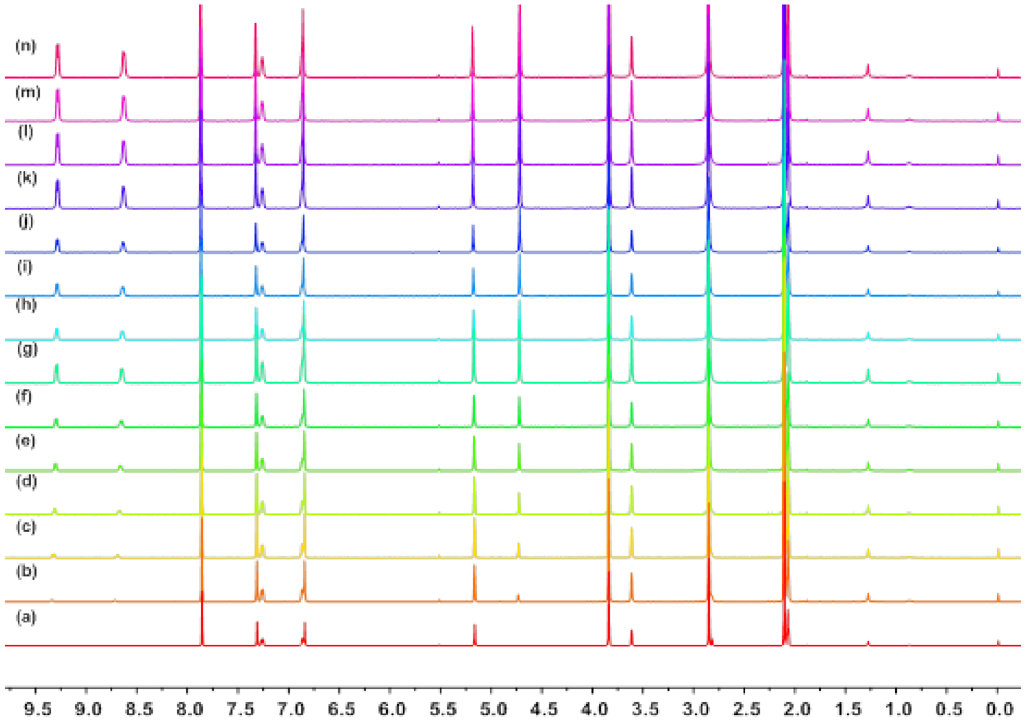


**Figure S24:** $^{1}$H NMR spectra (400 MHz, 298 K, $CDCl_3$/acetone-$d_6$ 1:2 (v/v)) of **H2** with different equivalents of **G1**: (a) 0.00, (b) 0.20, (c) 0.40, (d) 0.60, (e) 0.80, (f) 1.00, (g) 1.20, (h) 1.40, (i)1.60, (j) 1.80, (k) 2.00, (l) 2.20, (m) 2.40, (n) 2.50. [**H2**]$_0$ = 2.00 mM.

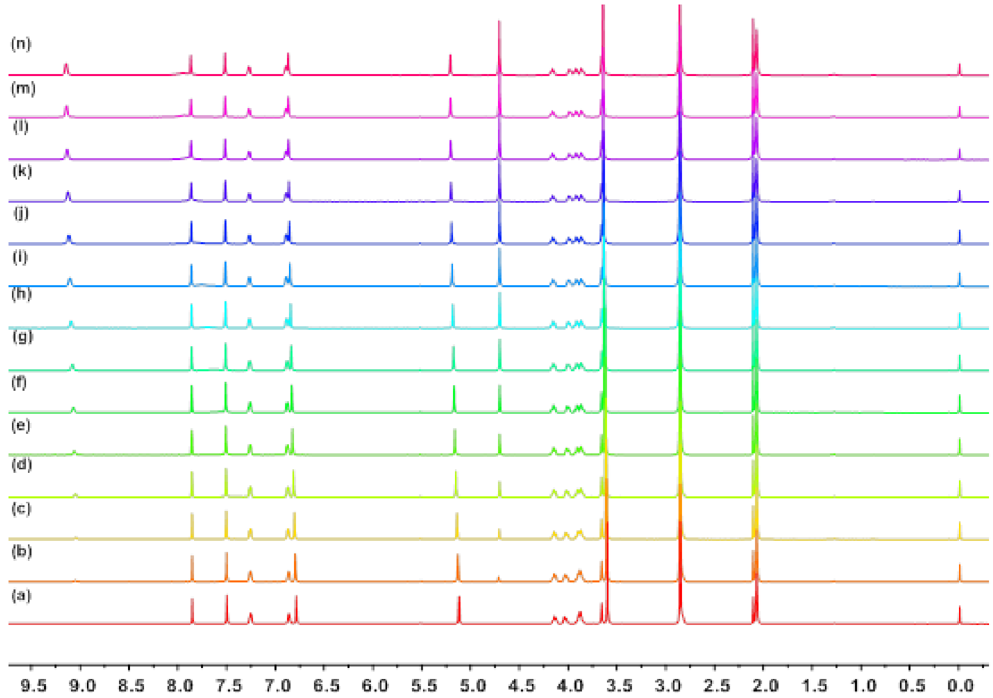


**Figure S25:** $^{1}$H NMR spectra (400 MHz, 298 K, $CDCl_3$/acetone-$d_6$ 1:2 (v/v)) of **H4** with different equivalents of **G1**: (a) 0.00, (b) 0.20, (c) 0.40, (d) 0.60, (e) 0.80, (f) 1.00, (g) 1.20, (h) 1.40, (i)1.60, (j) 1.80, (k) 2.00, (l) 2.20, (m) 2.40, (n) 2.50. $[\textbf{H4}]_0$ = 2.00 mM.

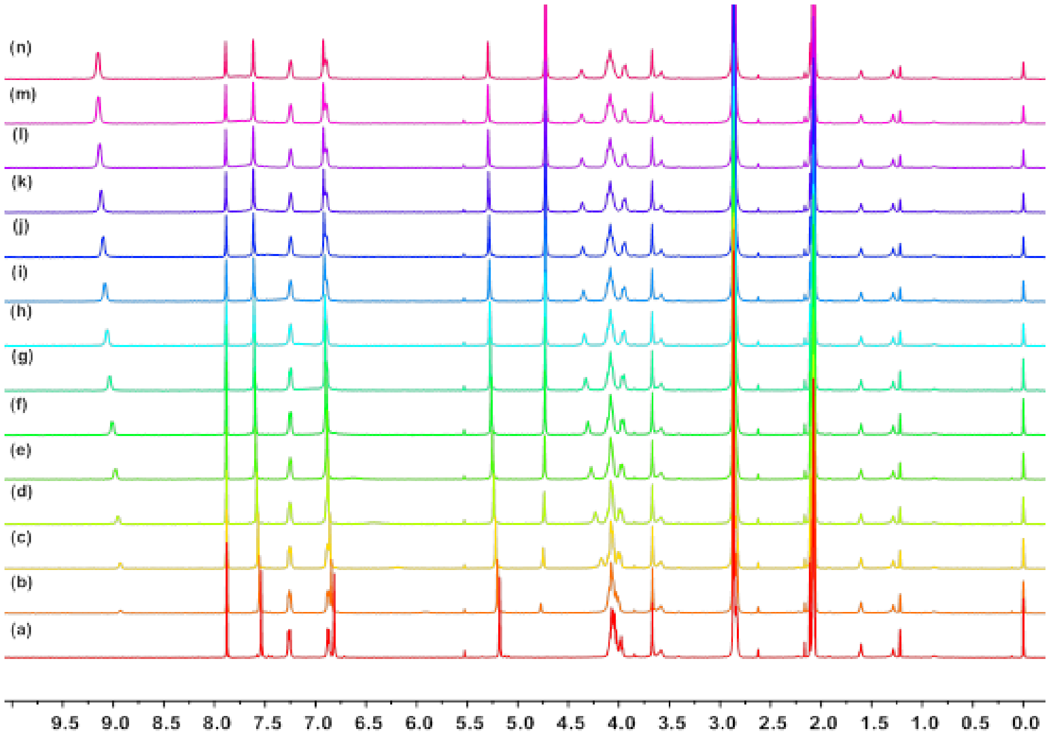


**Figure S26:** $^{1}$H NMR spectra (400 MHz, 298 K, $CDCl_3$/acetone-$d_6$ 1:2 (v/v)) of **H5** with different equivalents of **G1**: (a) 0.00, (b) 0.20, (c) 0.40, (d) 0.60, (e) 0.80, (f) 1.00, (g) 1.20, (h) 1.40, (i)1.60, (j) 1.80, (k) 2.00, (l) 2.20, (m) 2.40, (n) 2.50. $[\textbf{H5}]_0$ = 2.00 mM.

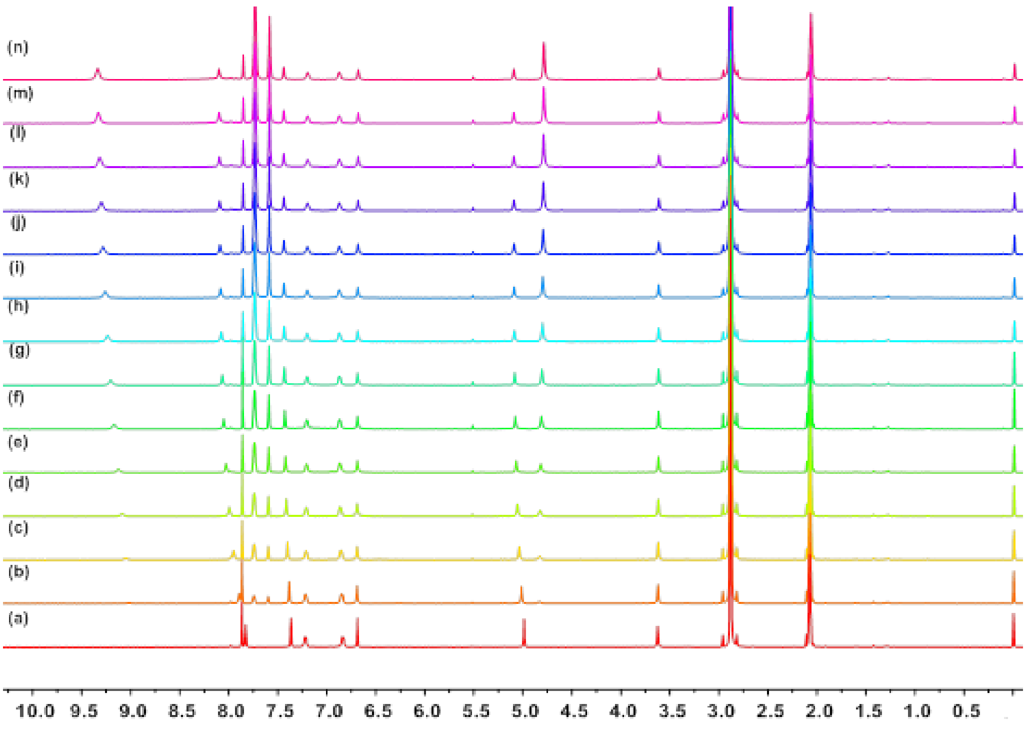


**Figure S27:** $^1$H NMR spectra (400 MHz, 298 K, $CDCl_3$/acetone-$d_6$ 1:2 (v/v)) of **H1** with different equivalents of **G2**: (a) 0.00, (b) 0.20, (c) 0.40, (d) 0.60, (e) 0.80, (f) 1.00, (g) 1.20, (h) 1.40, (i)1.60, (j) 1.80, (k) 2.00, (l) 2.20, (m) 2.40, (n) 2.50. [**H1**]$_0$ = 2.00 mM.

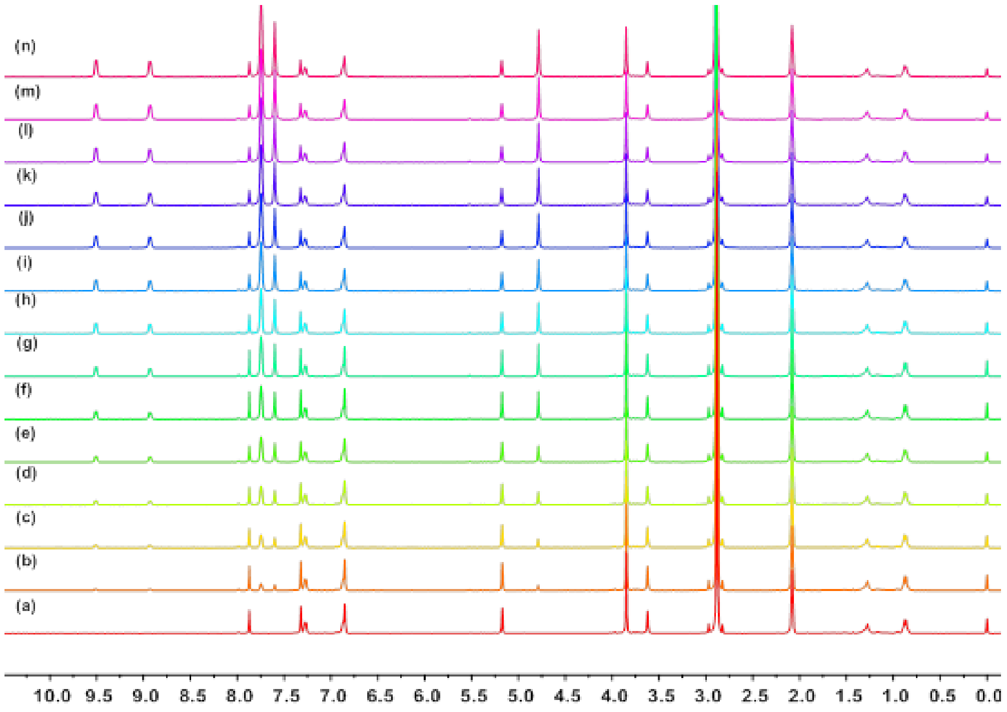


**Figure S28:** $^1$H NMR spectra (400 MHz, 298 K, $CDCl_3$/acetone-$d_6$ 1:2 (v/v)) of **H2** with different equivalents of **G2**: (a) 0.00, (b) 0.20, (c) 0.40, (d) 0.60, (e) 0.80, (f) 1.00, (g) 1.20, (h) 1.40, (i)1.60, (j) 1.80, (k) 2.00, (l) 2.20, (m) 2.40, (n) 2.50. [**H2**]$_0$ = 2.00 mM.

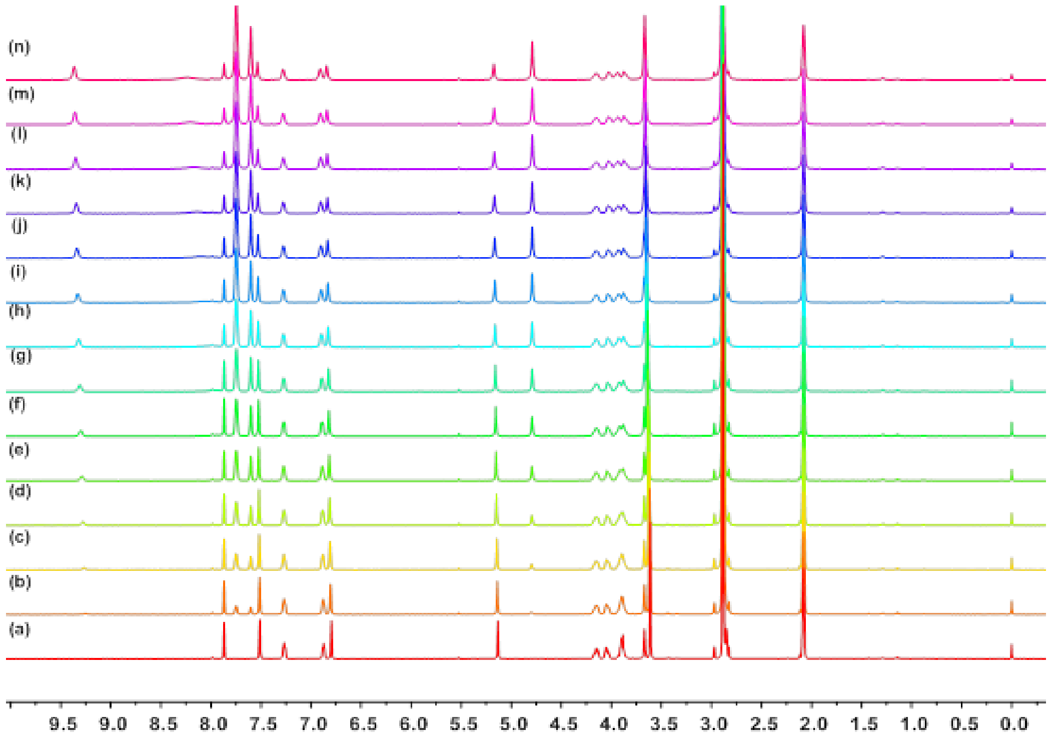


**Figure S29:** $^1$H NMR spectra (400 MHz, 298 K, $CDCl_3$/acetone-$d_6$ 1:2 (v/v)) of **H4** with different equivalents of **G2**: (a) 0.00, (b) 0.20, (c) 0.40, (d) 0.60, (e) 0.80, (f) 1.00, (g) 1.20, (h) 1.40, (i)1.60, (j) 1.80, (k) 2.00, (l) 2.20, (m) 2.40, (n) 2.50. $[\mathbf{H4}]_0$ = 2.00 mM.

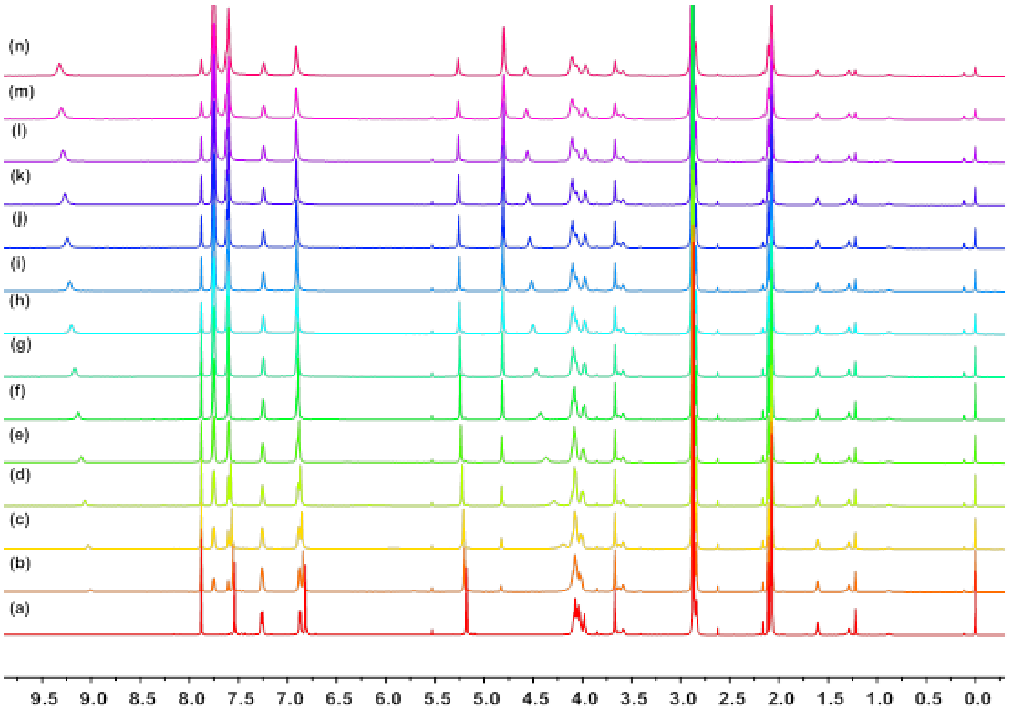


**Figure S30:** $^1$H NMR spectra (500 MHz, 298 K, $CDCl_3$/acetone-$d_6$ 1:2 (v/v)) of **H5** with different equivalents of **G2**: (a) 0.00, (b) 0.20, (c) 0.40, (d) 0.60, (e) 0.80, (f) 1.00, (g) 1.20, (h) 1.40, (i)1.60, (j) 1.80, (k) 2.00, (l) 2.20, (m) 2.40, (n) 2.50. $[\mathbf{H5}]_0$ = 2.00 mM.

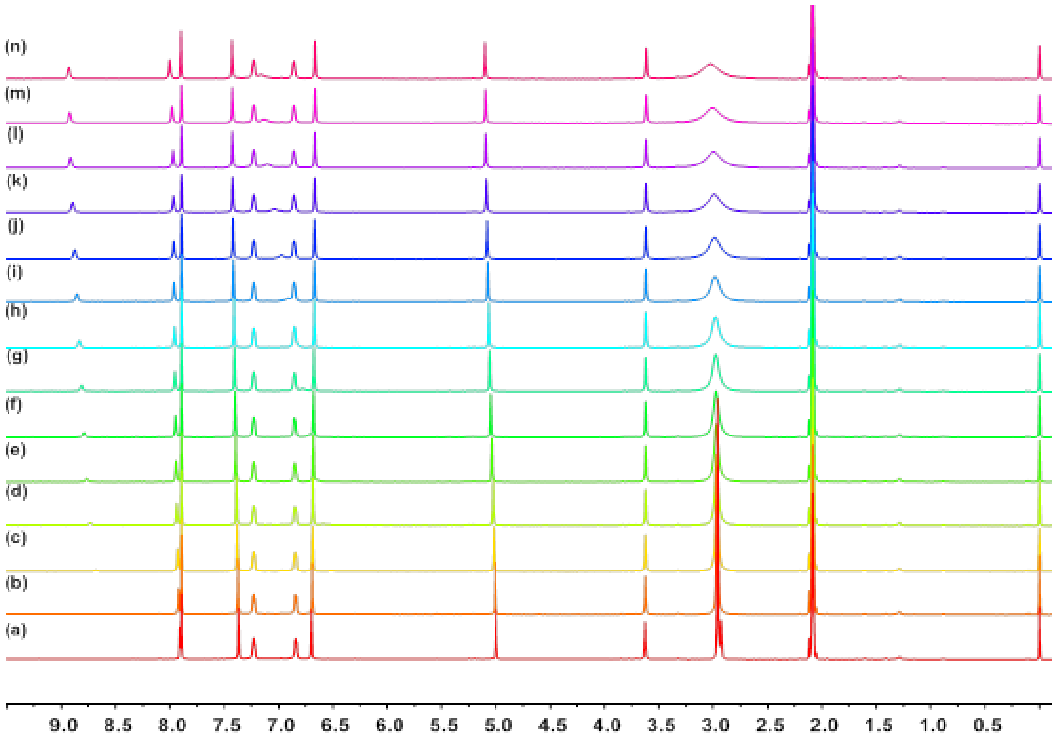


**Figure S31:** $^{1}$H NMR spectra (500 MHz, 298 K, $CDCl_3$/acetone-$d_6$ 1:2 (v/v)) of **H1** with different equivalents of **G3**: (a) 0.00, (b) 0.20, (c) 0.40, (d) 0.60, (e) 0.80, (f) 1.00, (g) 1.20, (h) 1.40, (i)1.60, (j) 1.80, (k) 2.00, (l) 2.20, (m) 2.40, (n) 2.50. [**H1**]$_0$ = 2.00 mM.

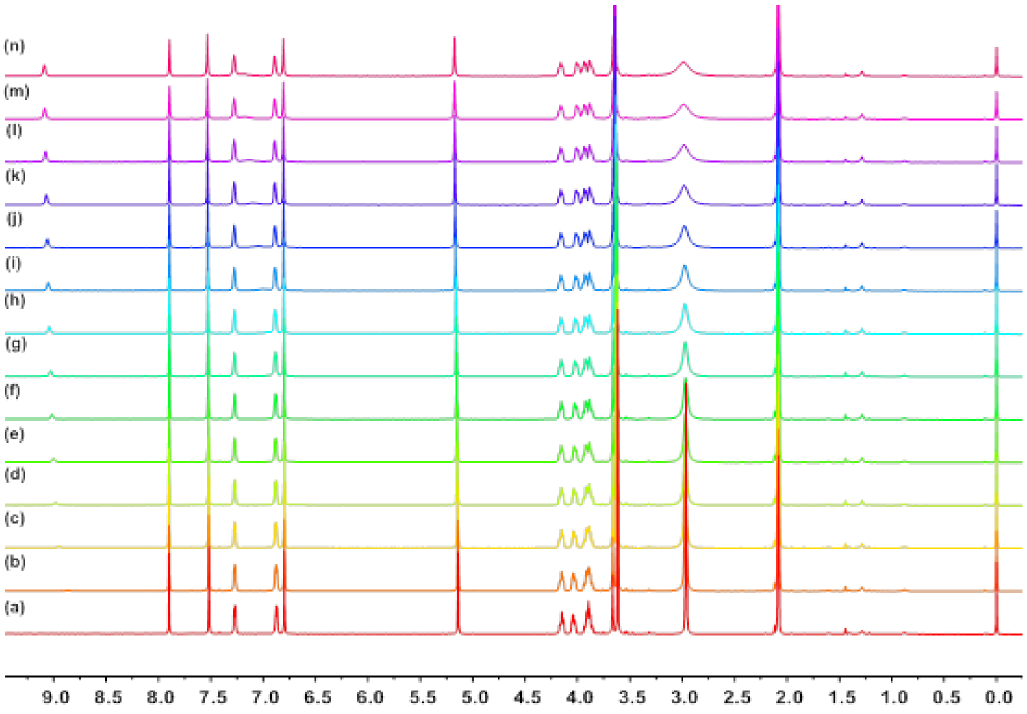


**Figure S32:** $^{1}$H NMR spectra (500 MHz, 298 K, $CDCl_3$/acetone-$d_6$ 1:2 (v/v)) of **H1** with different equivalents of **G3**: (a) 0.00, (b) 0.20, (c) 0.40, (d) 0.60, (e) 0.80, (f) 1.00, (g) 1.20, (h) 1.40, (i)1.60, (j) 1.80, (k) 2.00, (l) 2.20, (m) 2.40, (n) 2.50. [**H4**]$_0$ = 2.00 mM.

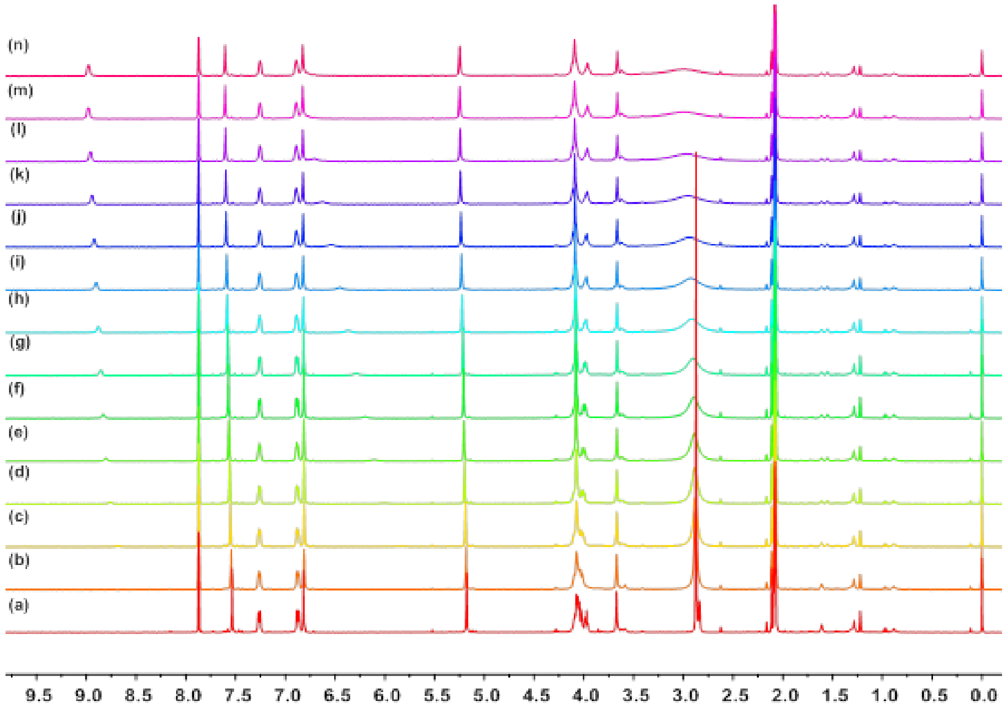


**Figure S33:** $^{1}$H NMR spectra (400 MHz, 298 K, $CDCl_3$/acetone-$d_6$ 1:2 (v/v)) of **H5** with different equivalents of **G3**: (a) 0.00, (b) 0.20, (c) 0.40, (d) 0.60, (e) 0.80, (f) 1.00, (g) 1.20, (h) 1.40, (i)1.60, (j) 1.80, (k) 2.00, (l) 2.20, (m) 2.40, (n) 2.50. [**H5**]$_0$ = 2.00 mM.

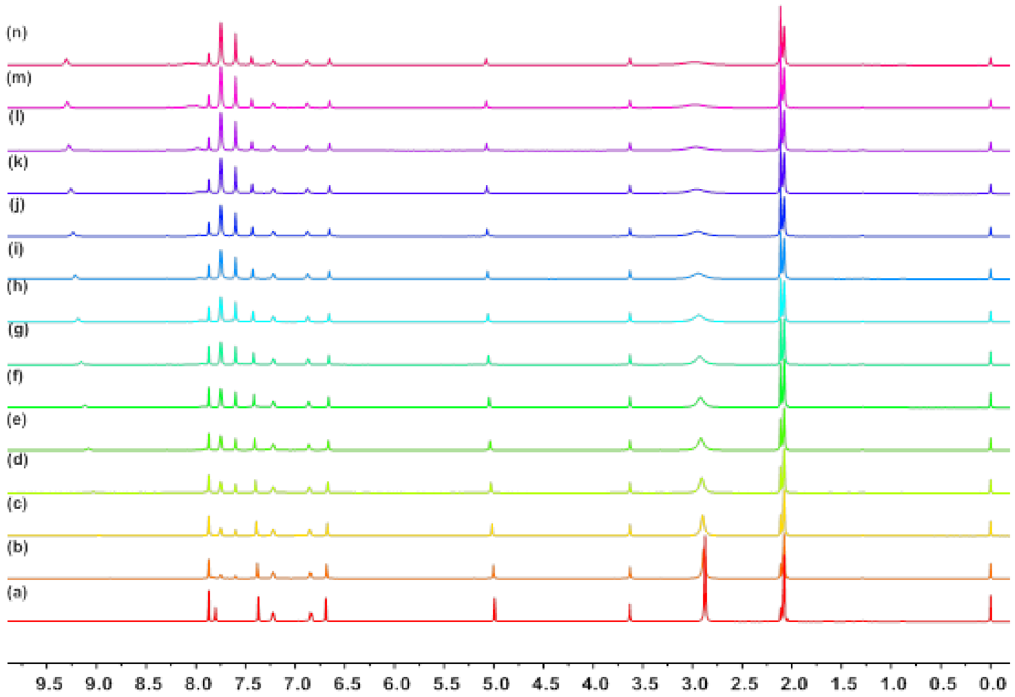


**Figure S34:** $^{1}$H NMR spectra (500 MHz, 298 K, $CDCl_3$/acetone-$d_6$ 1:2 (v/v)) of **H1** with different equivalents of **G4**: (a) 0.00, (b) 0.20, (c) 0.40, (d) 0.60, (e) 0.80, (f) 1.00, (g) 1.20, (h) 1.40, (i)1.60, (j) 1.80, (k) 2.00, (l) 2.20, (m) 2.40, (n) 2.50. [**H1**]$_0$ = 2.00 mM.

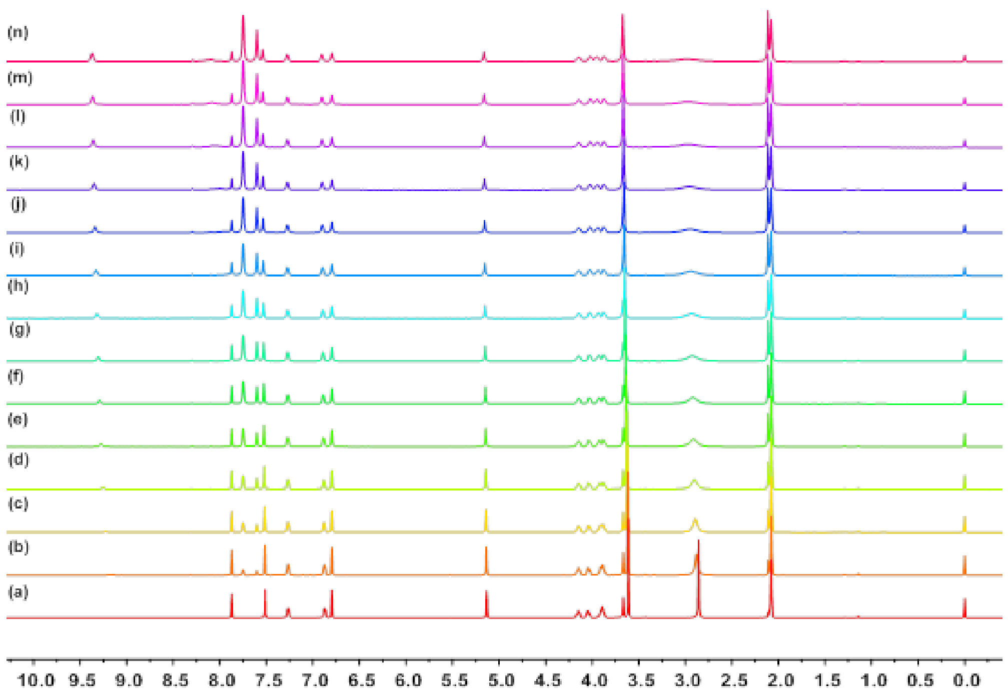


**Figure S35:** $^{1}$H NMR spectra (500 MHz, 298 K, $CDCl_3$/acetone-$d_6$ 1:2 (v/v)) of **H4** with different equivalents of **G4**: (a) 0.00, (b) 0.20, (c) 0.40, (d) 0.60, (e) 0.80, (f) 1.00, (g) 1.20, (h) 1.40, (i)1.60, (j) 1.80, (k) 2.00, (l) 2.20, (m) 2.40, (n) 2.50. $[\textbf{H4}]_0$ = 2.00 mM.

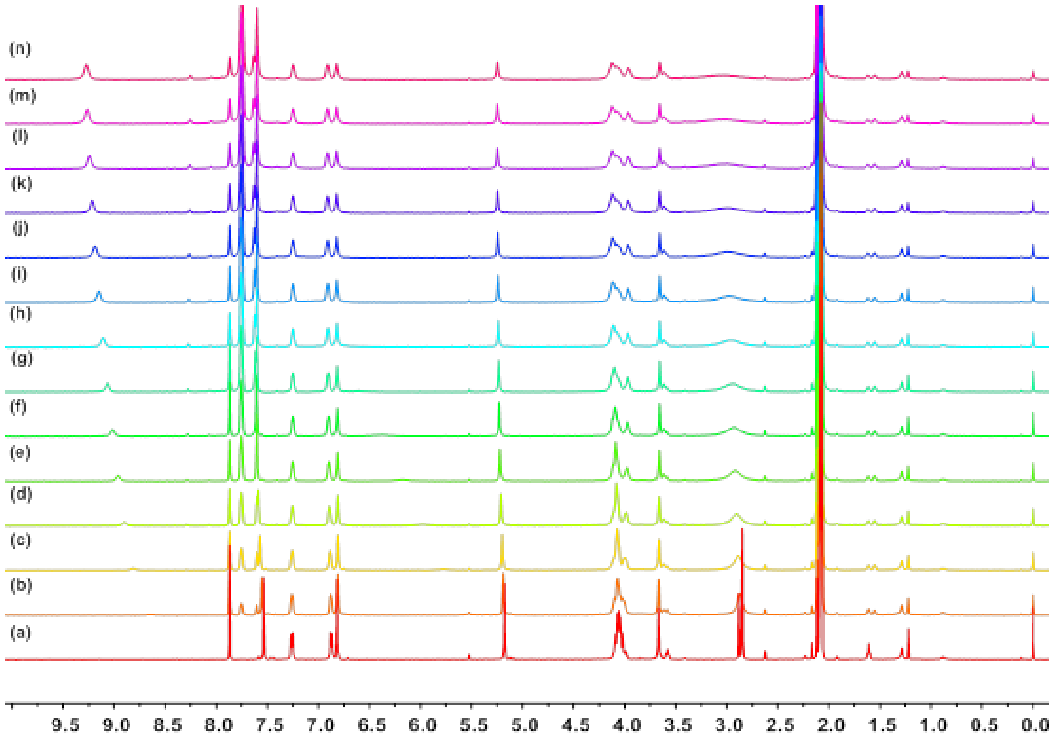


**Figure S36:** $^{1}$H NMR spectra (400 MHz, 298 K, $CDCl_3$/acetone-$d_6$ 1:2 (v/v)) of **H5** with different equivalents of **G4**: (a) 0.00, (b) 0.20, (c) 0.40, (d) 0.60, (e) 0.80, (f) 1.00, (g) 1.20, (h) 1.40, (i)1.60, (j) 1.80, (k) 2.00, (l) 2.20, (m) 2.40, (n) 2.50. $[\textbf{H5}]_0$ = 2.00 mM.

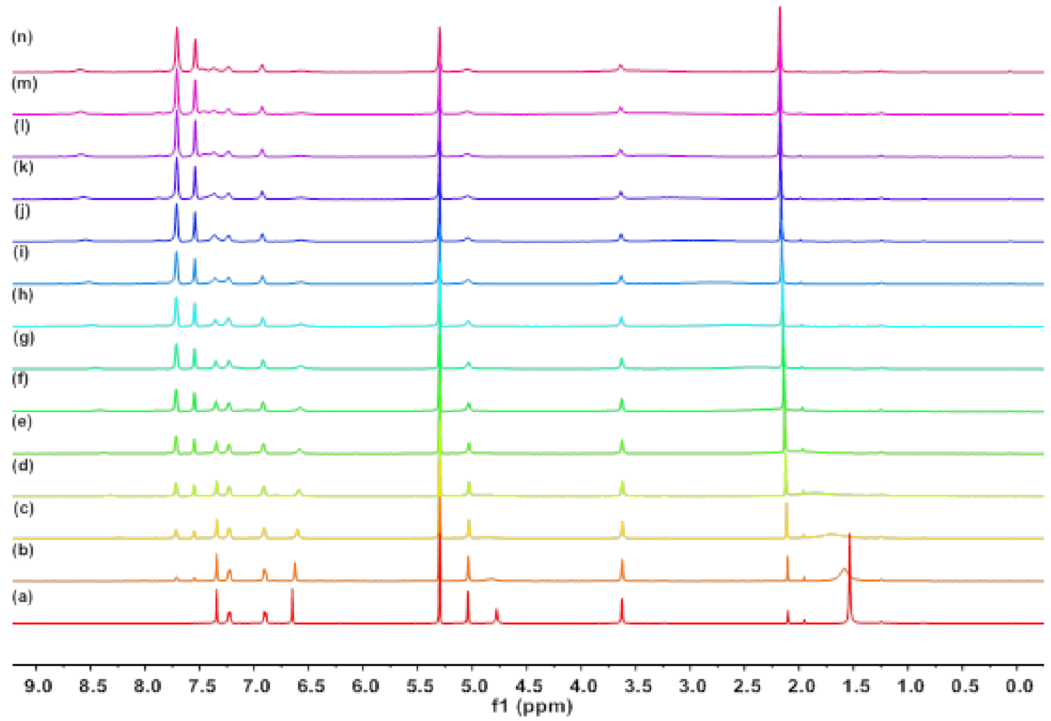


**Figure S37:** $^1$H NMR spectra (400 MHz, 298 K, $CD_2Cl_2$) of **H1** with different equivalents of **G4**: (a) 0.00, (b) 0.20, (c) 0.40, (d) 0.60, (e) 0.80, (f) 1.00, (g) 1.20, (h) 1.40, (i)1.60, (j) 1.80, (k) 2.00, (l) 2.20, (m) 2.40, (n) 2.50. [**H1**]$_0$ = 2.00 mM.

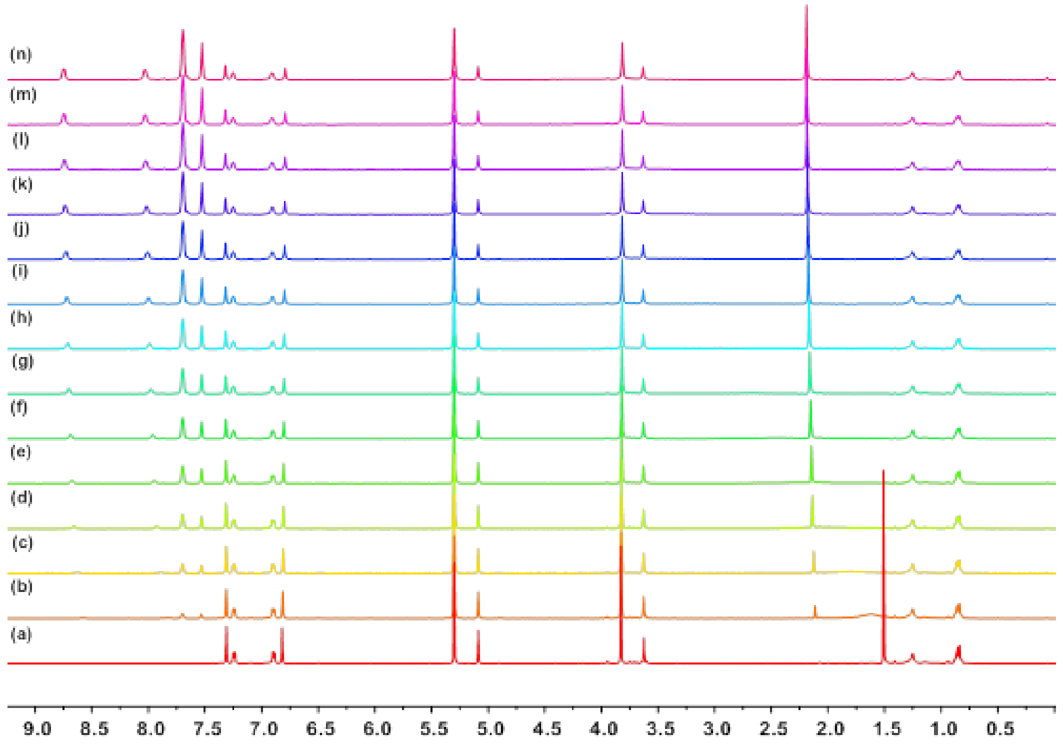


**Figure S38:** $^1$H NMR spectra (400 MHz, 298 K, $CD_2Cl_2$) of **H2** with different equivalents of **G4**: (a) 0.00, (b) 0.20, (c) 0.40, (d) 0.60, (e) 0.80, (f) 1.00, (g) 1.20, (h) 1.40, (i)1.60, (j) 1.80, (k) 2.00, (l) 2.20, (m) 2.40, (n) 2.50. [**H2**]$_0$ = 2.00 mM.

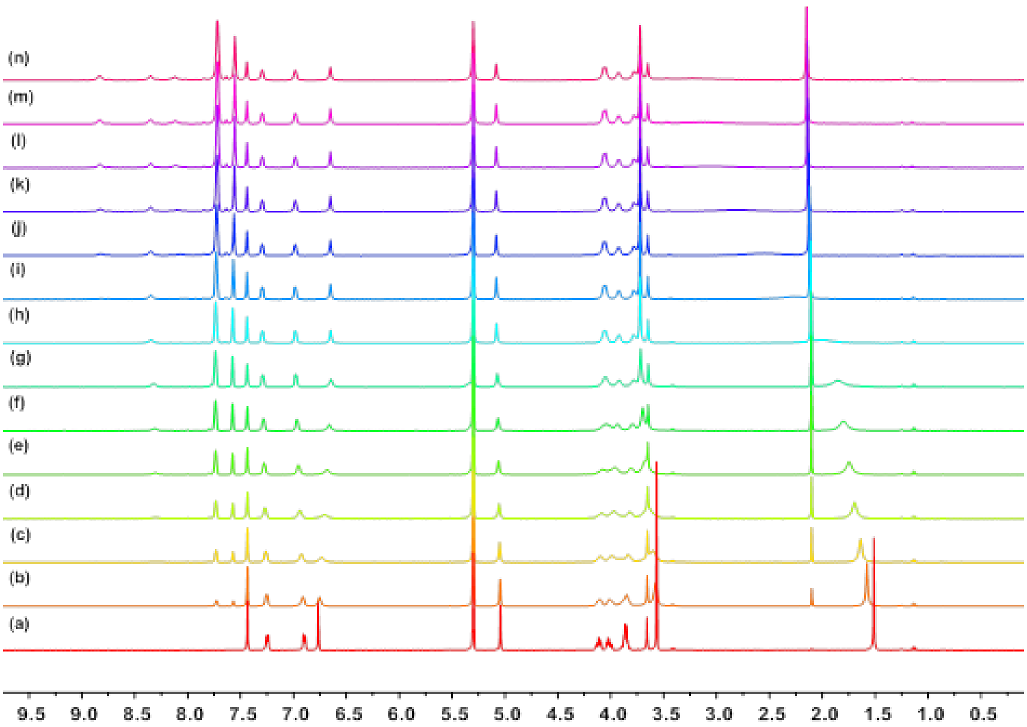


**Figure S39:** $^{1}$H NMR spectra (400 MHz, 298 K, $CD_2Cl_2$) of **H4** with different equivalents of **G4**: (a) 0.00, (b) 0.20, (c) 0.40, (d) 0.60, (e) 0.80, (f) 1.00, (g) 1.20, (h) 1.40, (i)1.60, (j) 1.80, (k) 2.00, (l) 2.20, (m) 2.40, (n) 2.50. [**H4**]$_0$ = 2.00 mM.

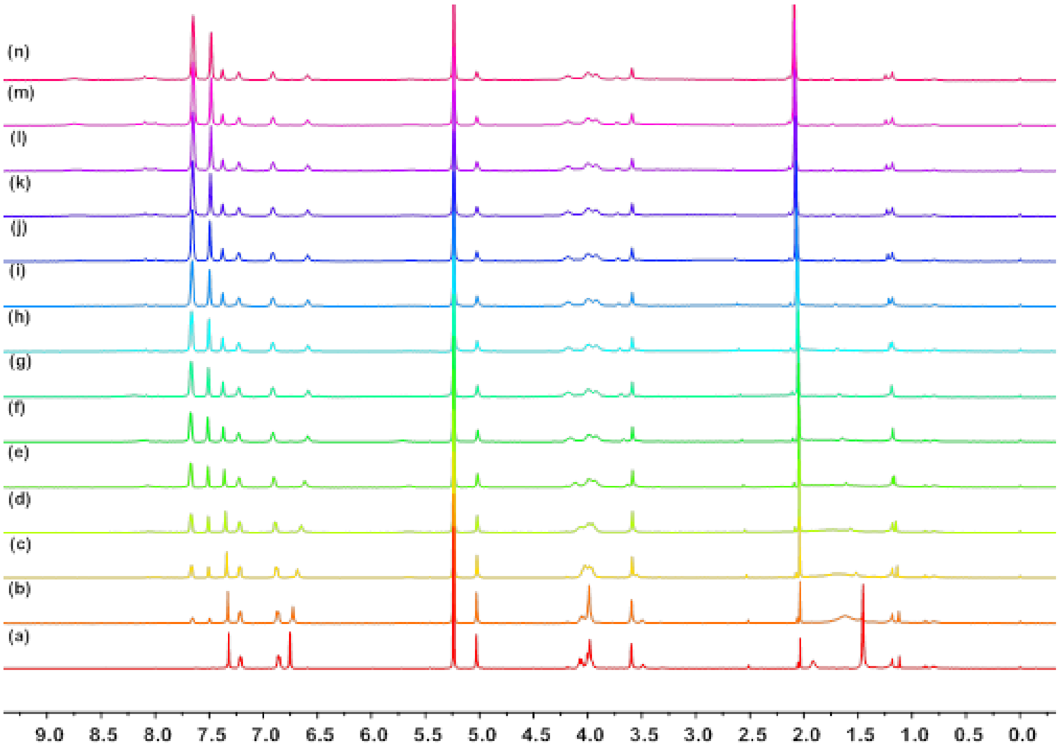


**Figure S40:** $^{1}$H NMR spectra (400 MHz, 298 K, $CD_2Cl_2$) of **H5** with different equivalents of **G4**: (a) 0.00, (b) 0.20, (c) 0.40, (d) 0.60, (e) 0.80, (f) 1.00, (g) 1.20, (h) 1.40, (i)1.60, (j) 1.80, (k) 2.00, (l) 2.20, (m) 2.40, (n) 2.50. [**H5**]$_0$ = 2.00 mM.

## 5. High-resolution mass spectra (ESI) for the complexes

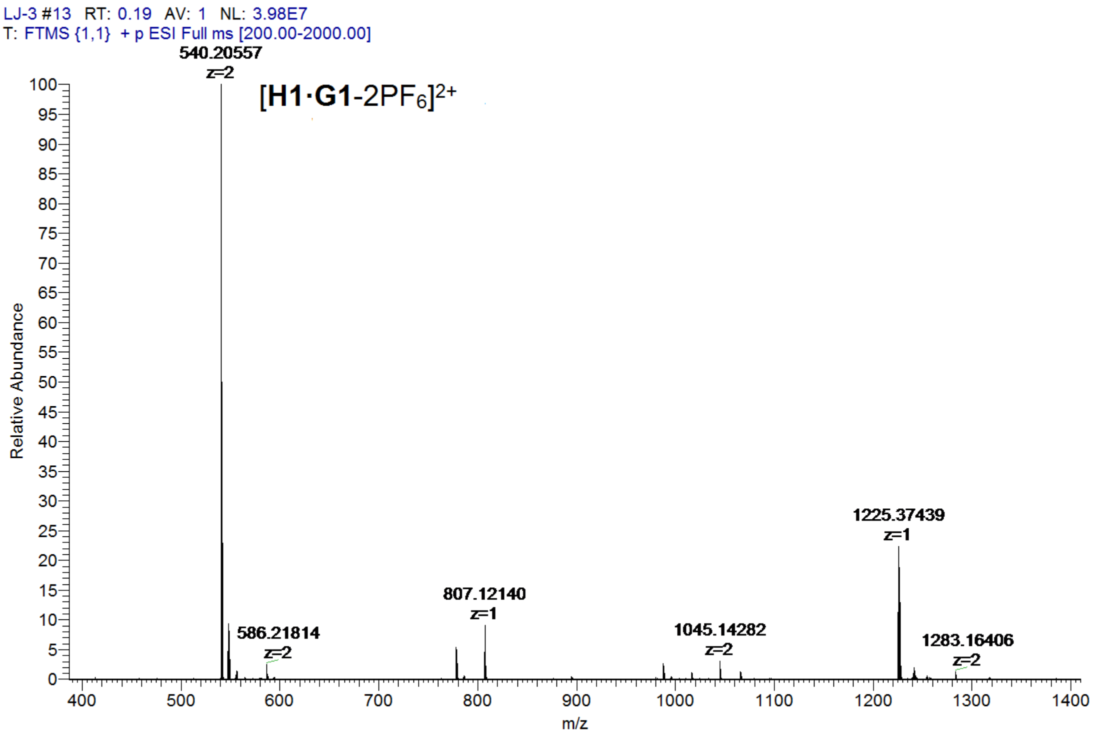


**Figure S41:** ESIMS spectrum of complex **H1·G1**.

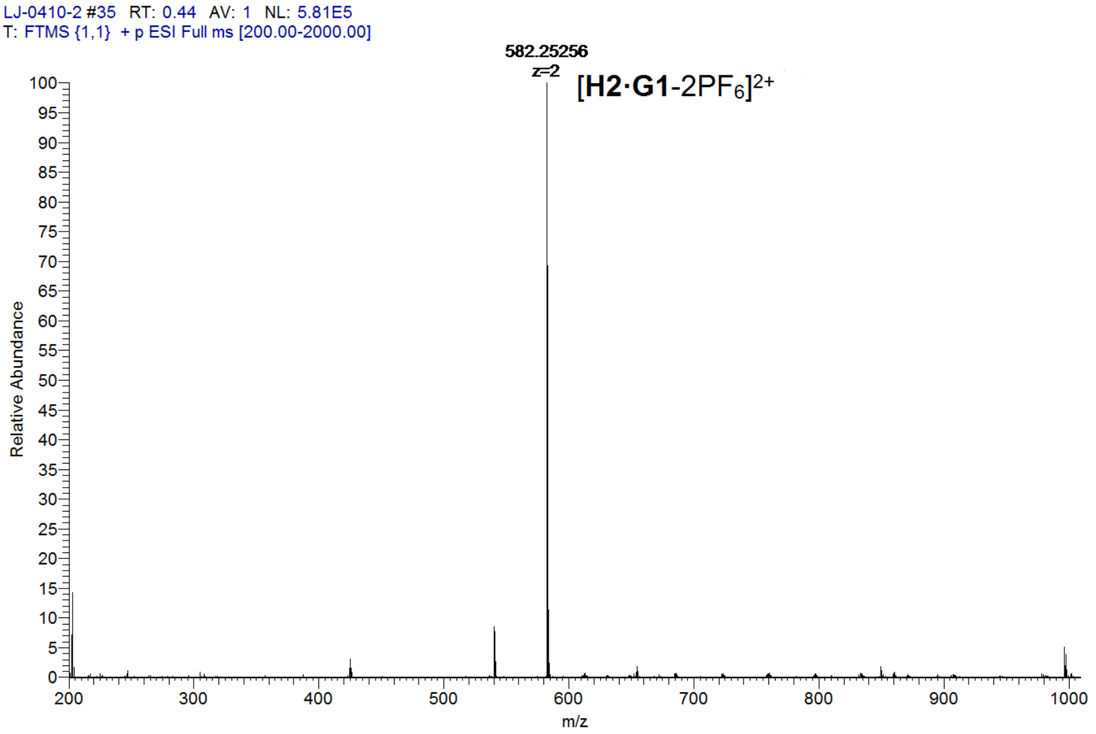


**Figure S42:** ESIMS spectrum of complex **H2·G1**.

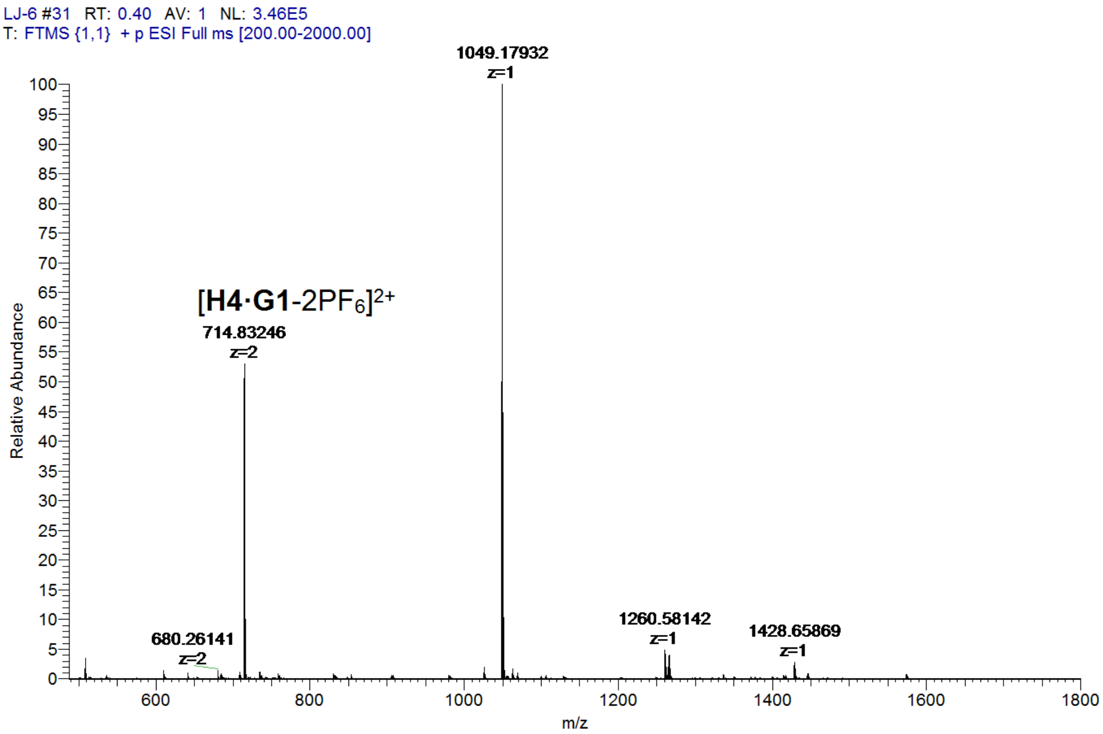


**Figure S43:** ESIMS spectrum of complex **H4·G1**.

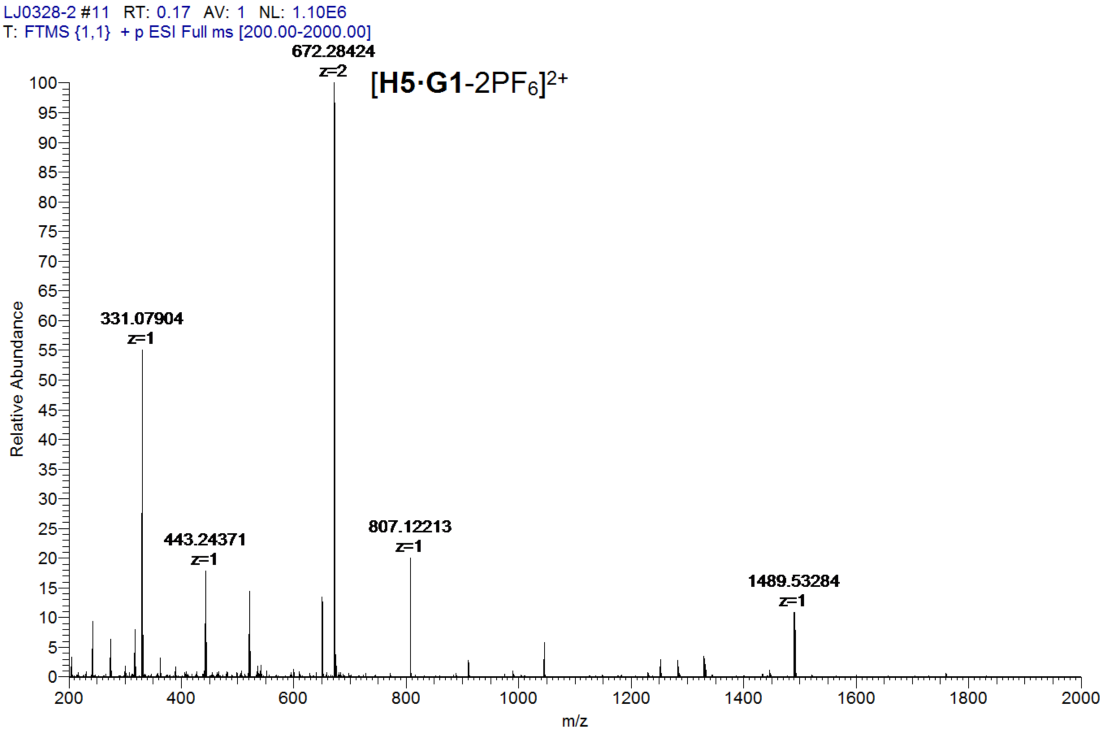


**Figure S44:** ESIMS spectrum of complex **H5·G1**.

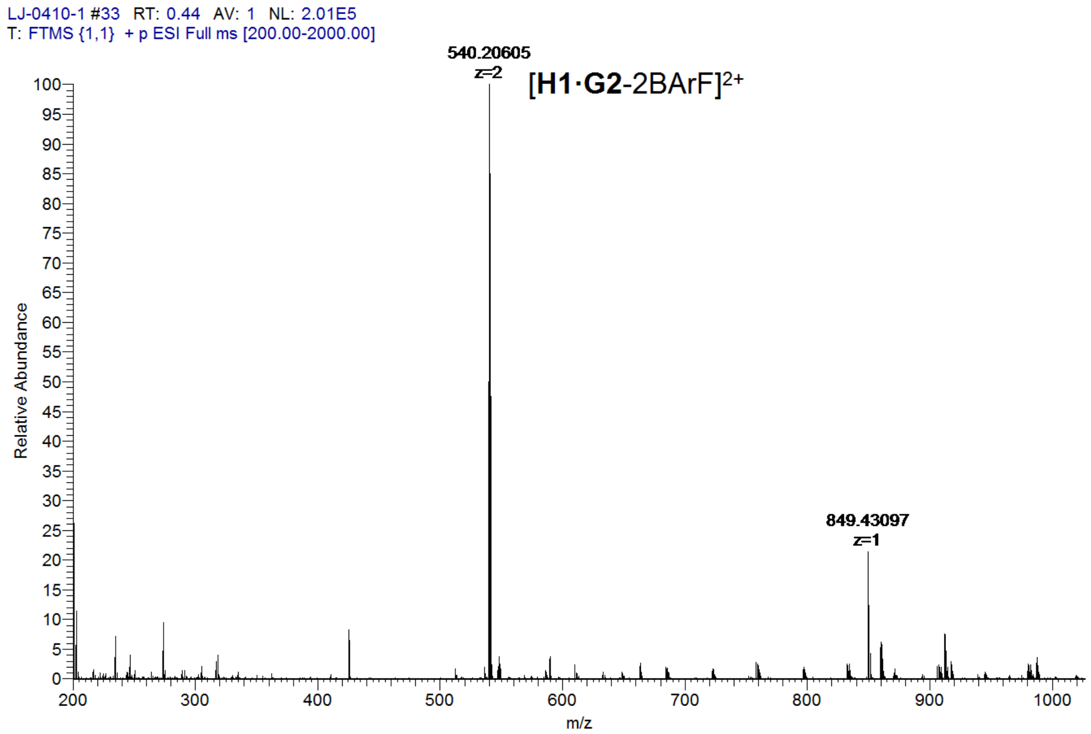


**Figure S45:** ESIMS spectrum of complex **H1**·**G2**.

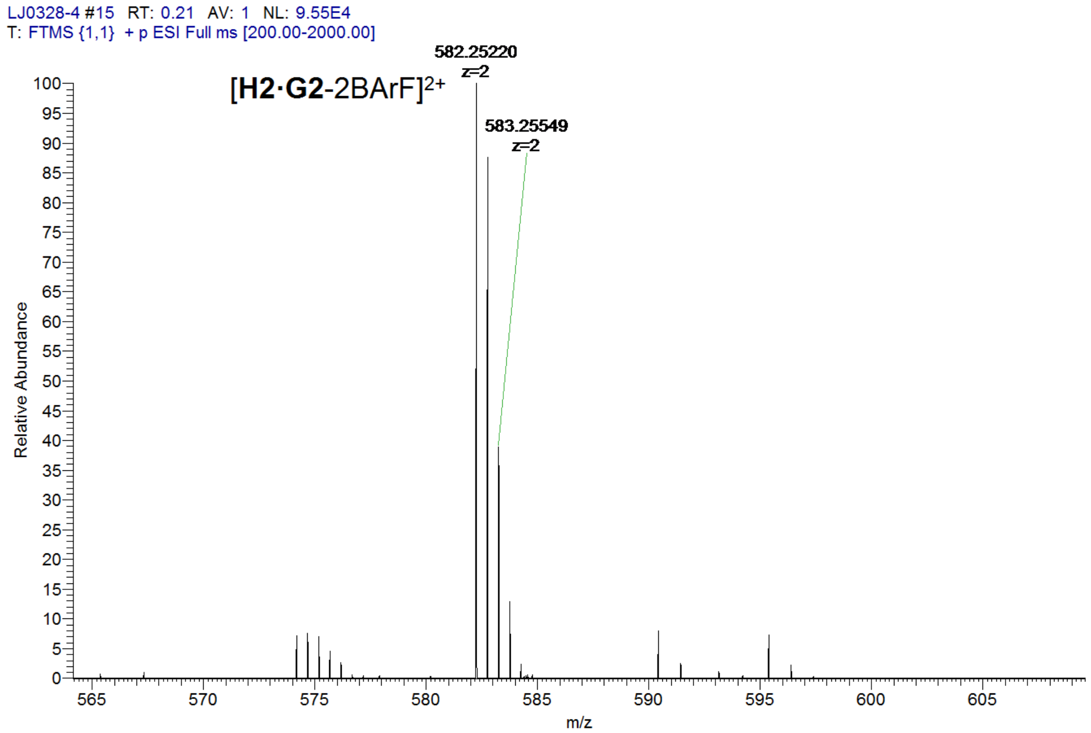


**Figure S46:** ESIMS spectrum of complex **H2**·**G2**.

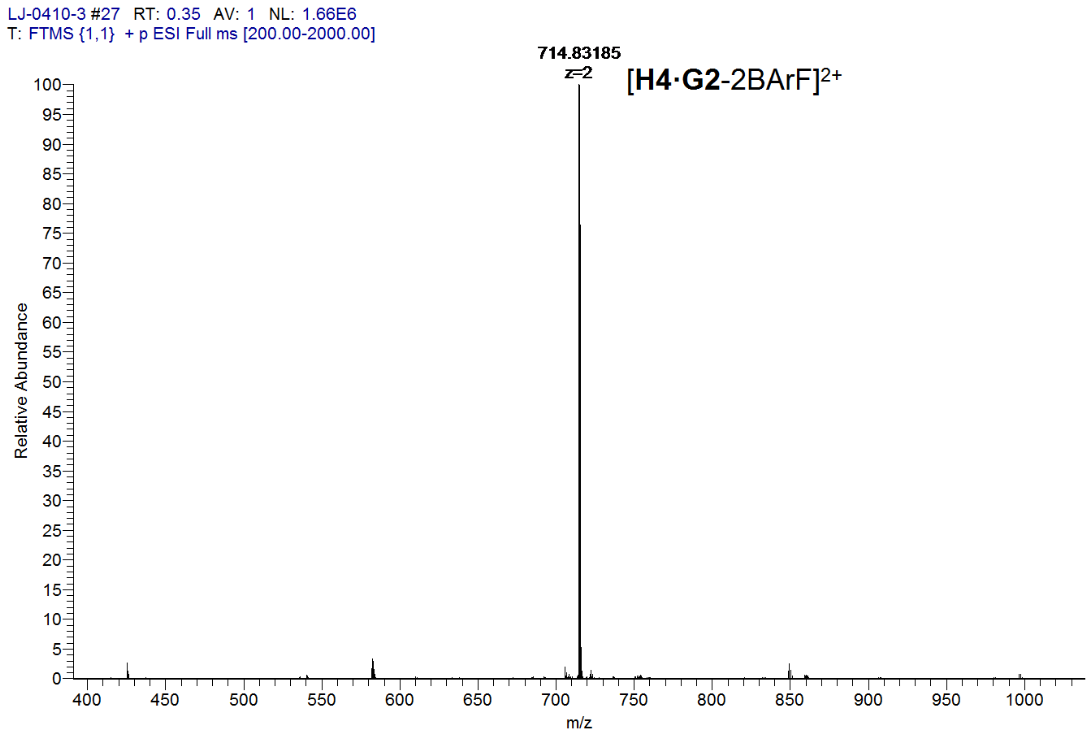


**Figure S47:** ESIMS spectrum of complex **H4**·**G2**.

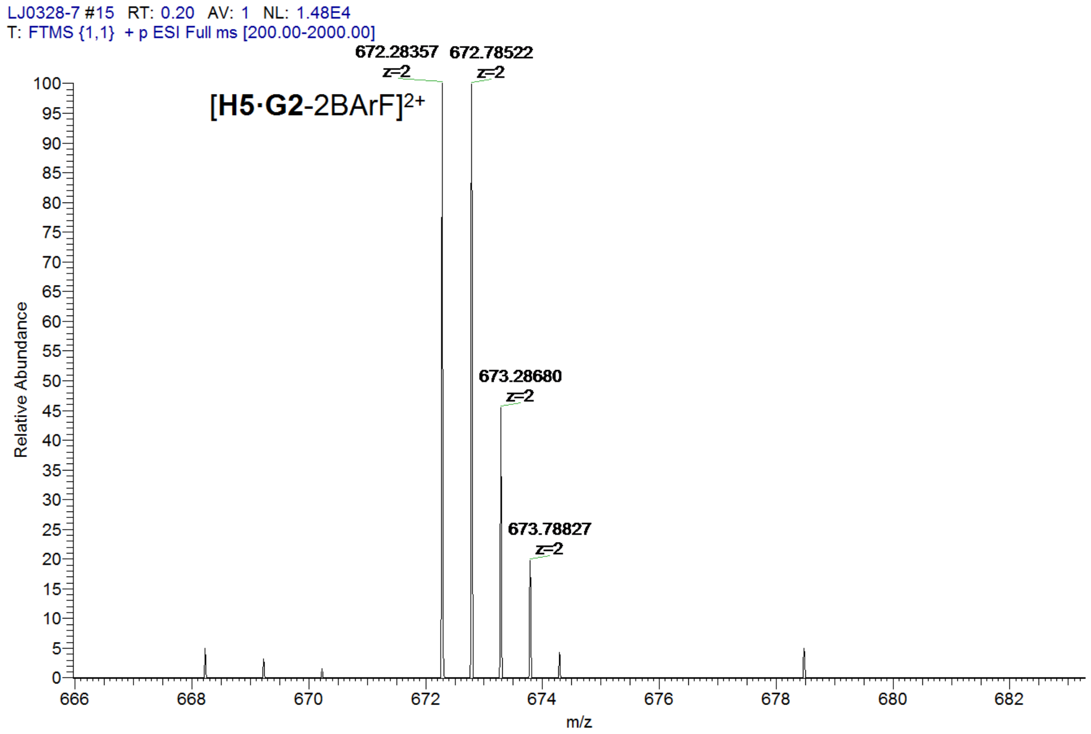


. **Figure S48:** ESIMS spectrum of complex **H5**·**G2**.

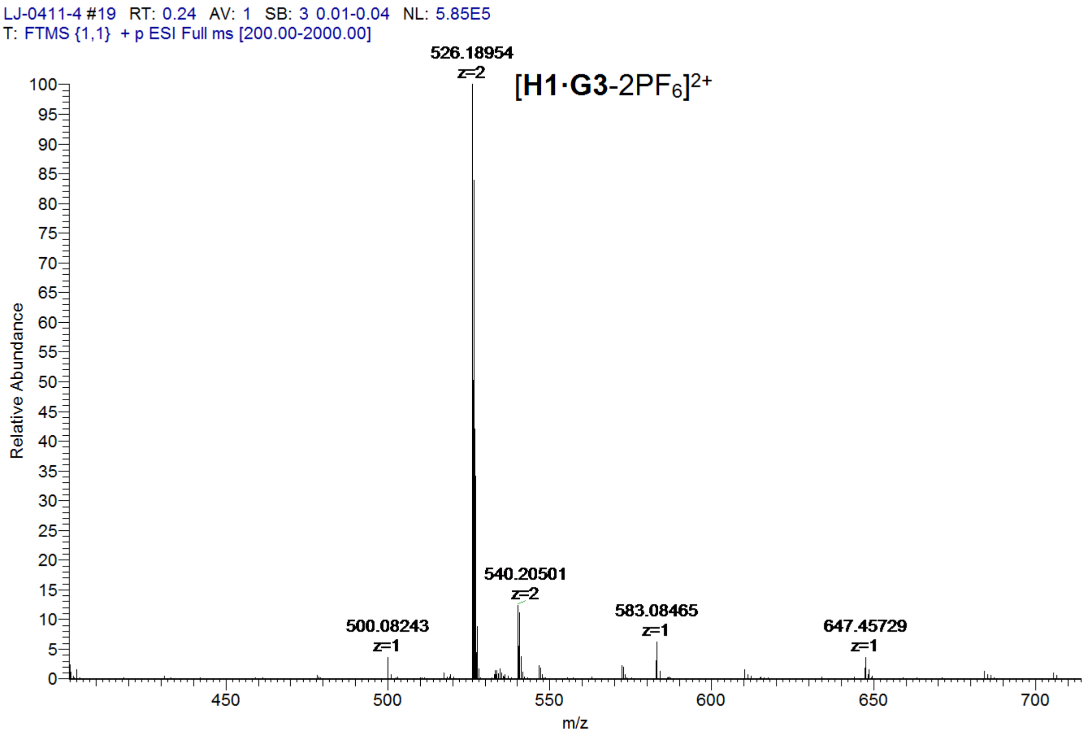


**Figure S49:** ESIMS spectrum of complex **H1·G3**

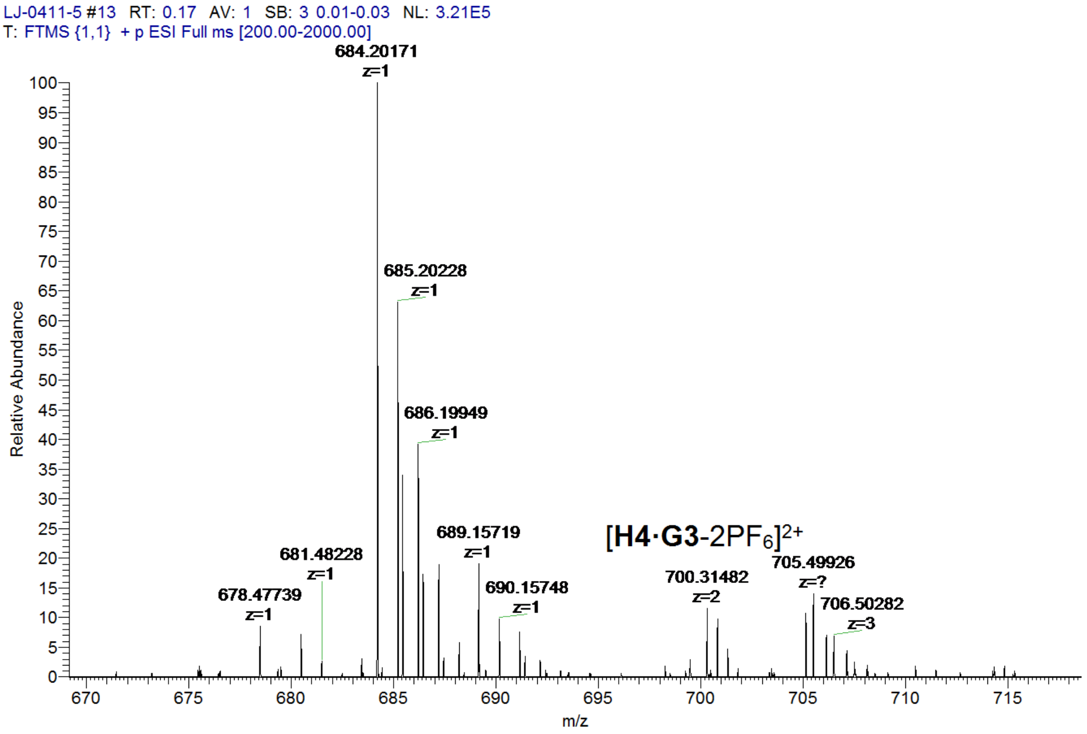


. **Figure S50:** ESIMS spectrum of complex **H4·G3**.

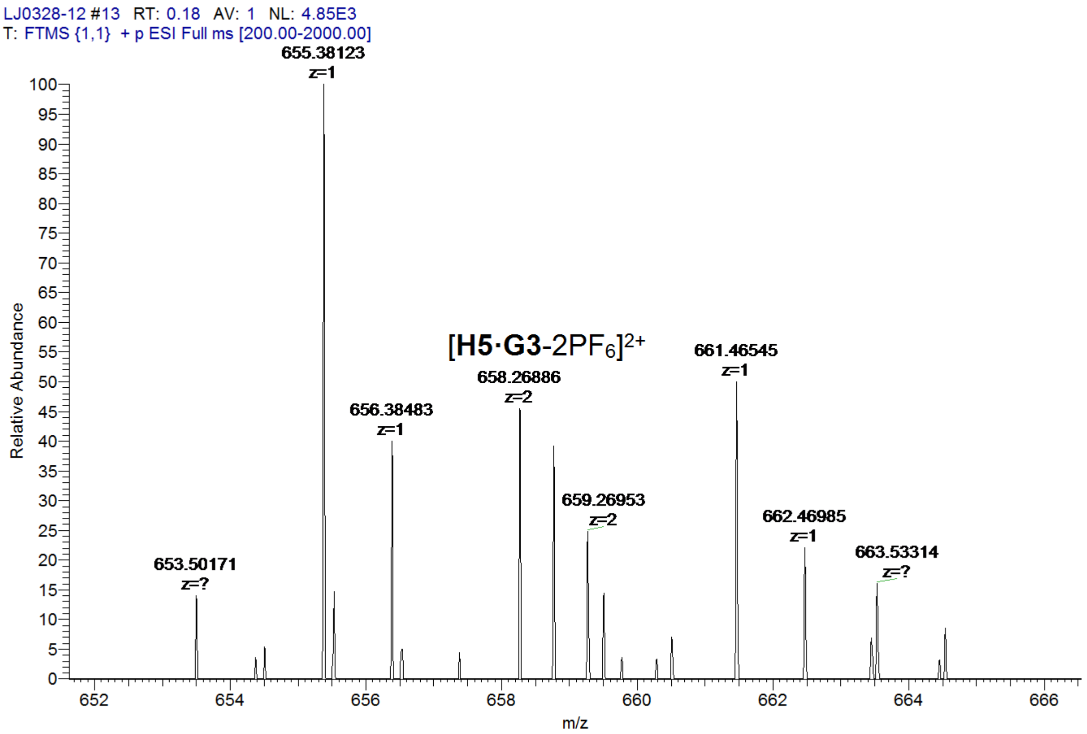


**Figure S51:** ESIMS spectrum of complex **H5·G3**.

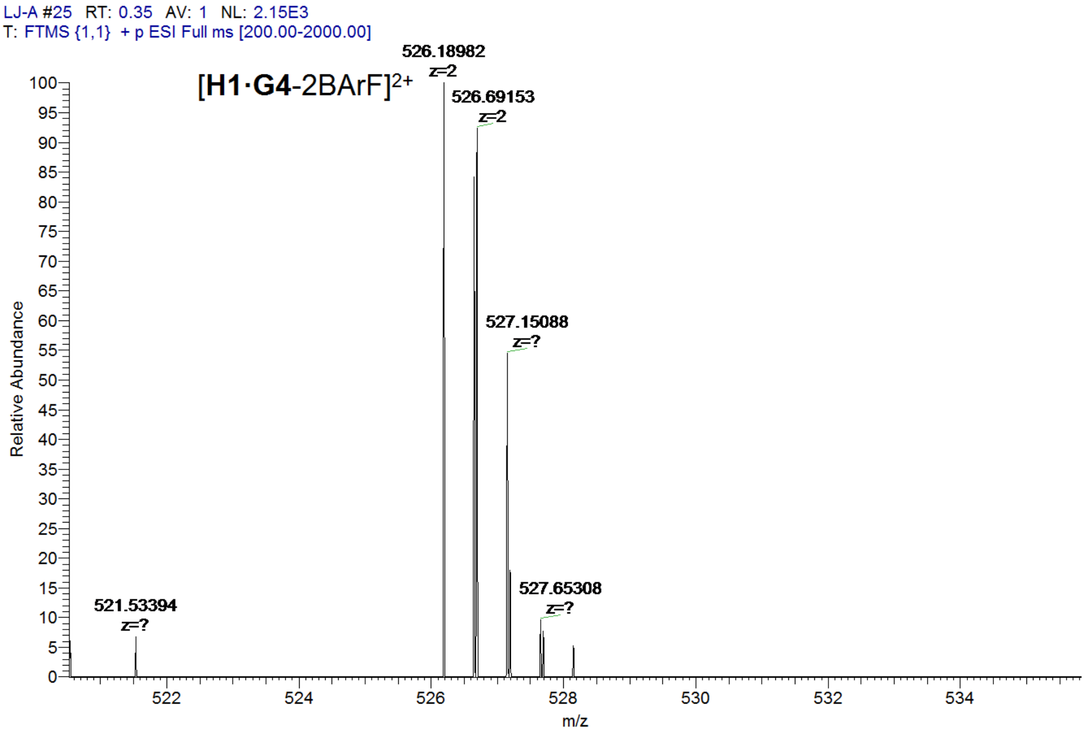


**Figure S52:** ESIMS spectrum of complex **H1·G4**

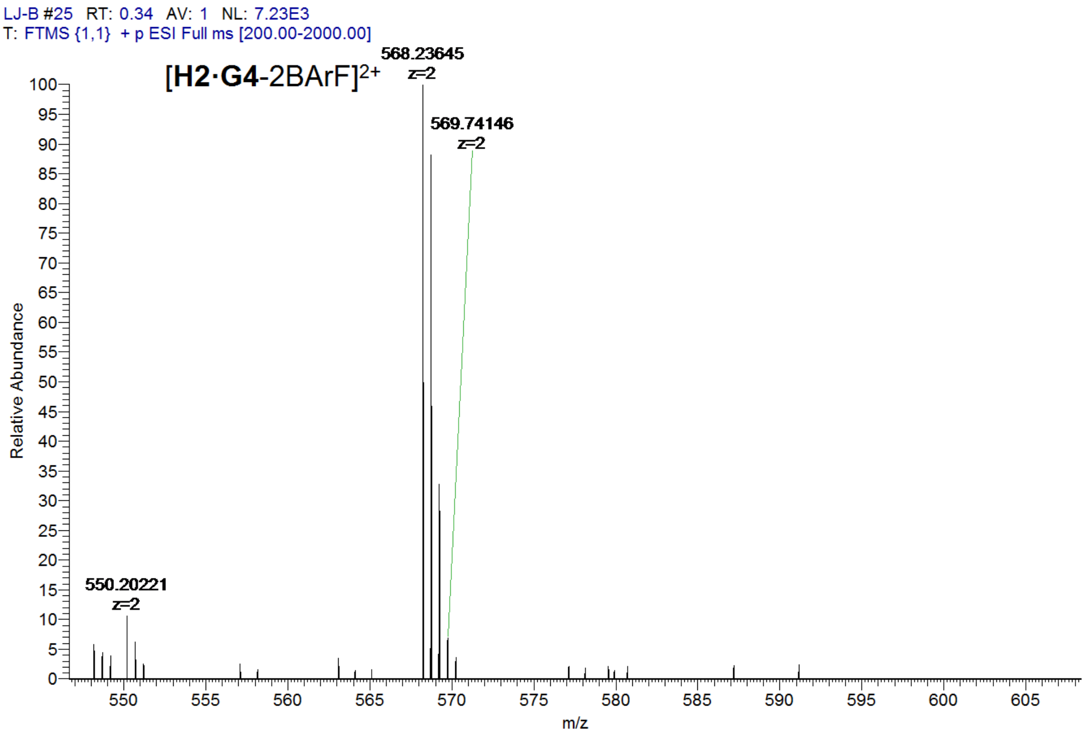


**Figure S53:** ESIMS spectrum of complex **H2·G4**

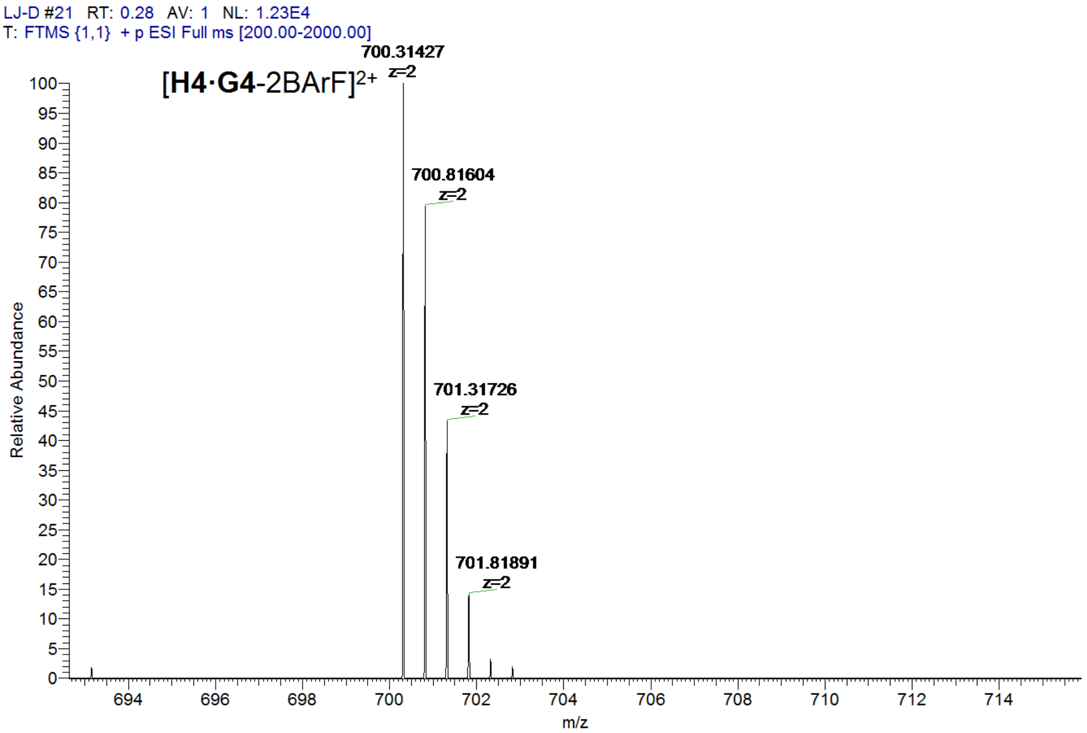


. **Figure S54:** ESIMS spectrum of complex **H4·G4**

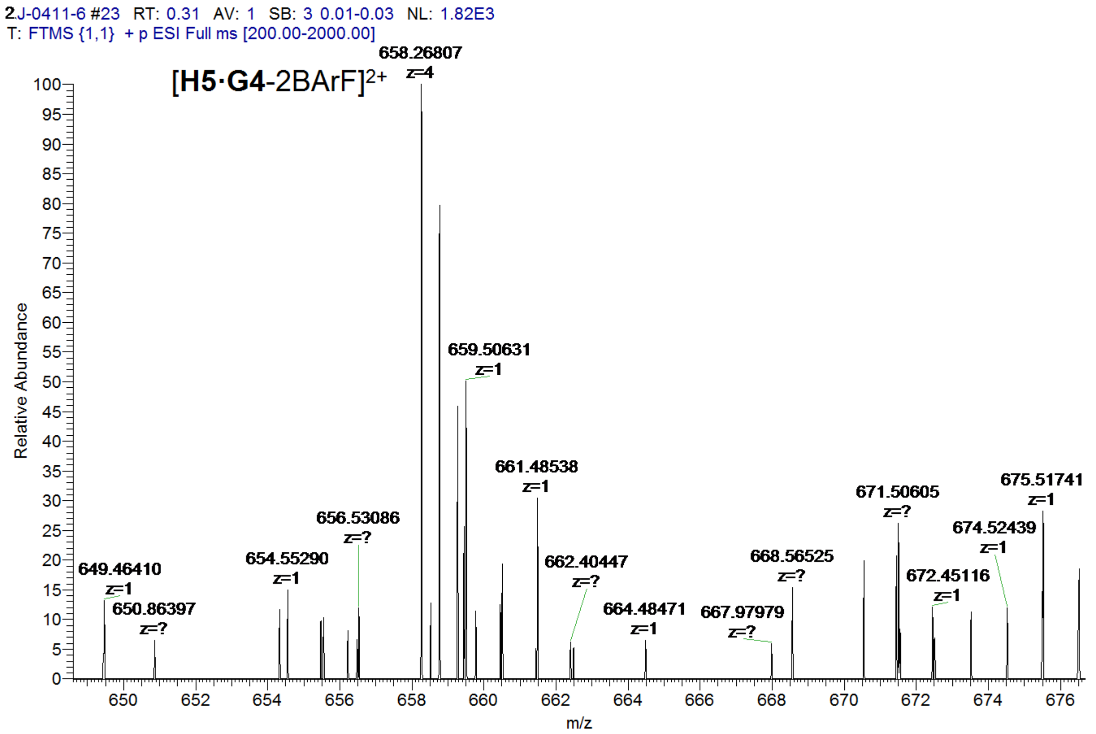


**Figure S55:** ESIMS spectrum of complex **H5·G4**

## 6. Nonlinear curve fitting and job plots for the complexes

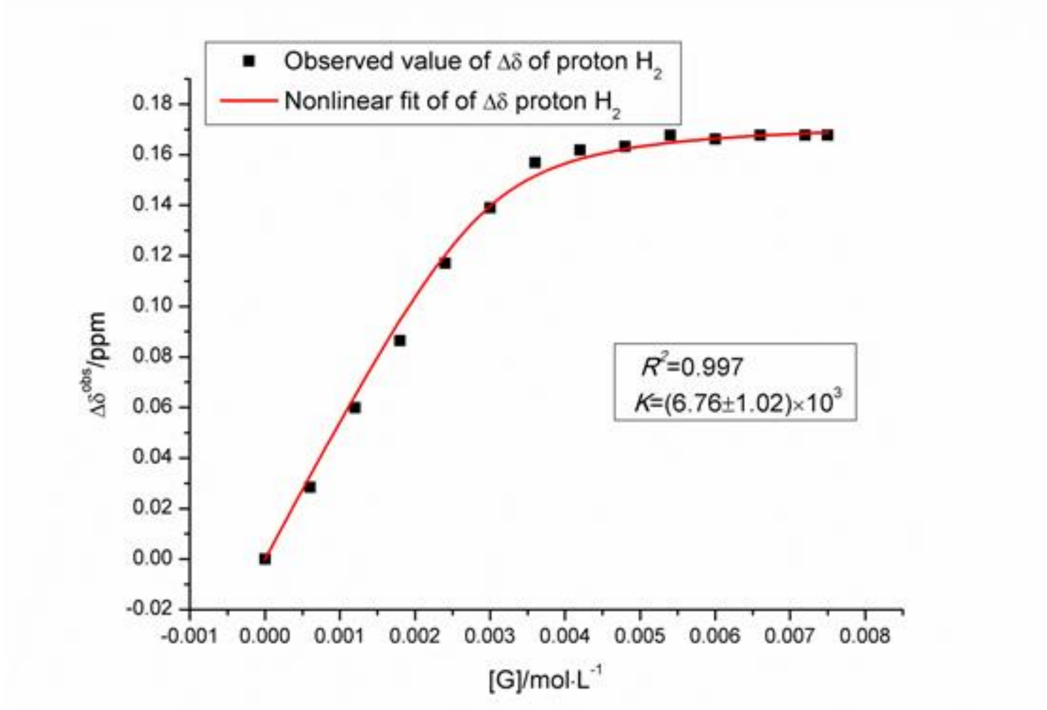


**Figure S56:** Plots of $\Delta\delta_{obs}$ (ppm) for the bridgehead proton $H_2$ of **H1** vs **G1** concentration in $CDCl_3$/acetone-*d*$_6$ 1:2 (v/v) at 298 K.

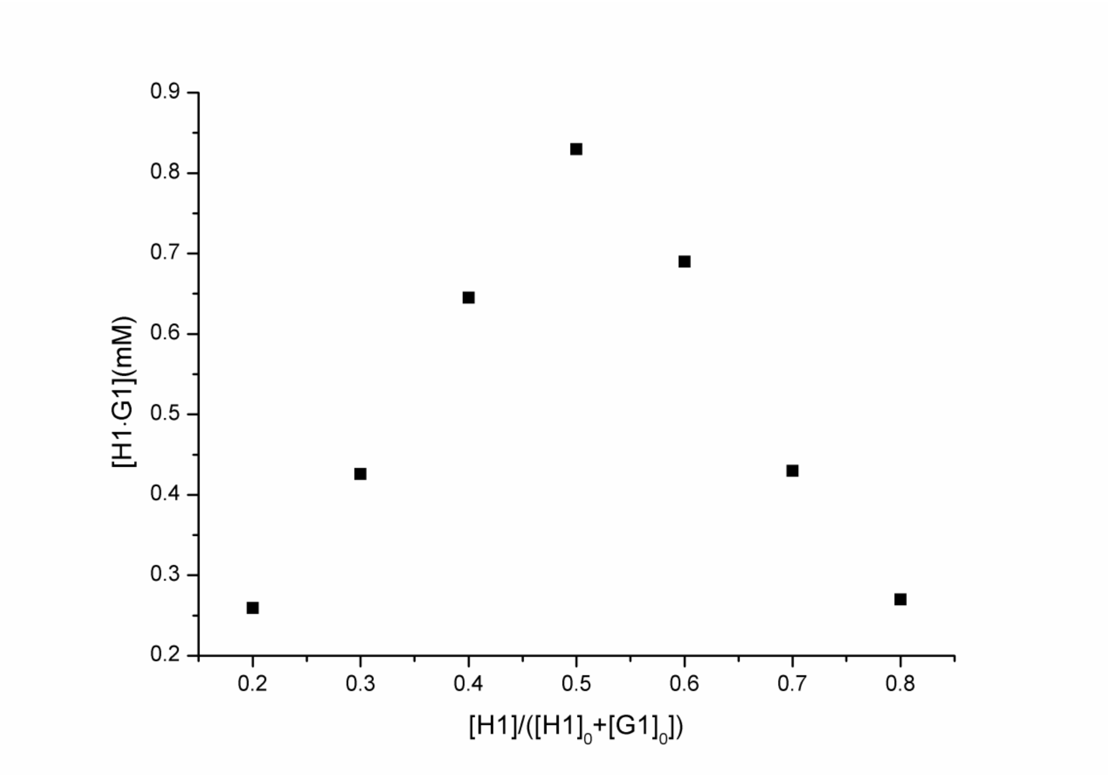


**Figure S57**: Job plot for the complexation of **H1** and **G1** in $CDCl_3$/acetone-$d_6$ 1:2 (v/v) at 298 K.

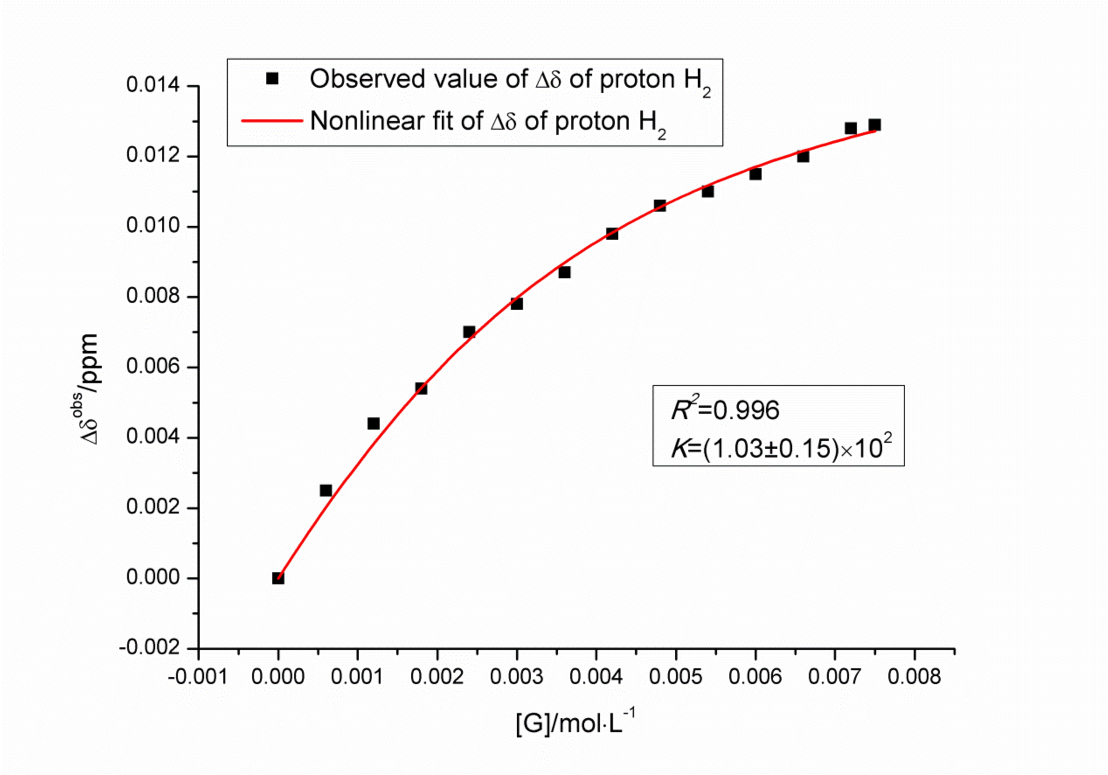


**Figure S58:** Plots of $\Delta\delta_{obs}$ (ppm) for the bridgehead proton $H_2$ of **H2** vs **G1** concentration in $CDCl_3$/acetone-$d_6$ 1:2 (v/v) at 298 K.

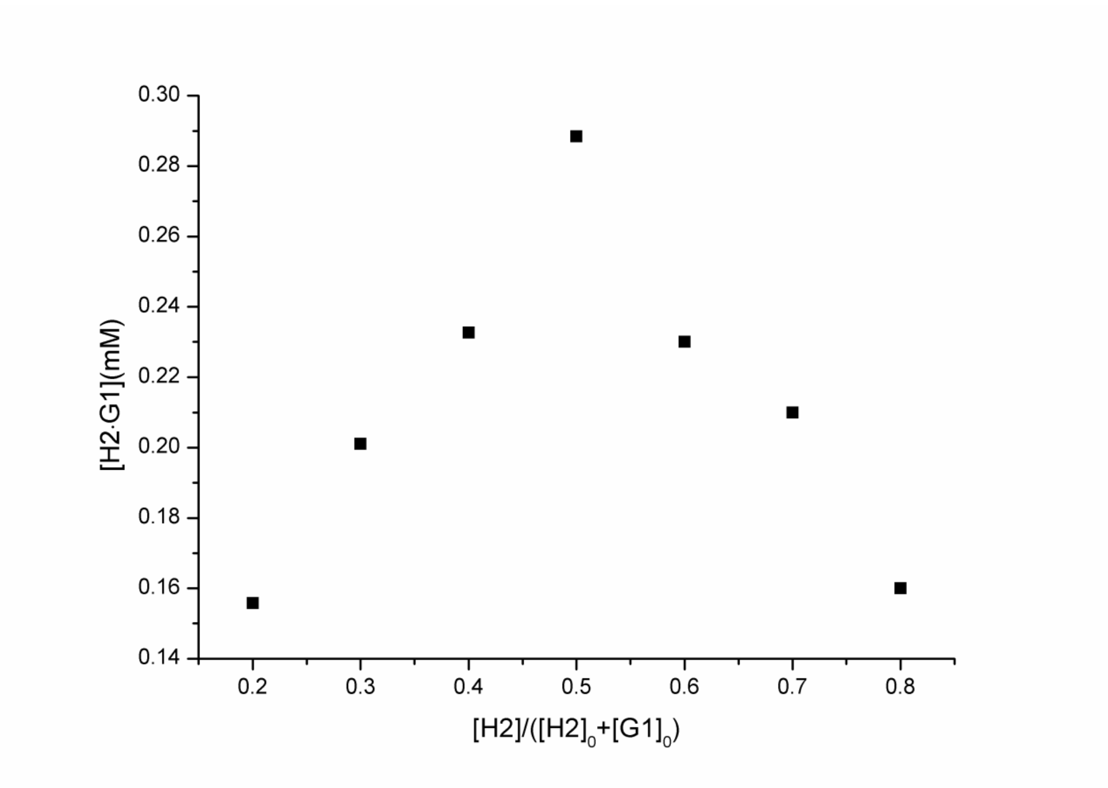


**Figure S59:** Job plot for the complexation of **H2** and **G1** in $CDCl_3$/acetone-$d_6$ 1:2 (v/v) at 298 K.

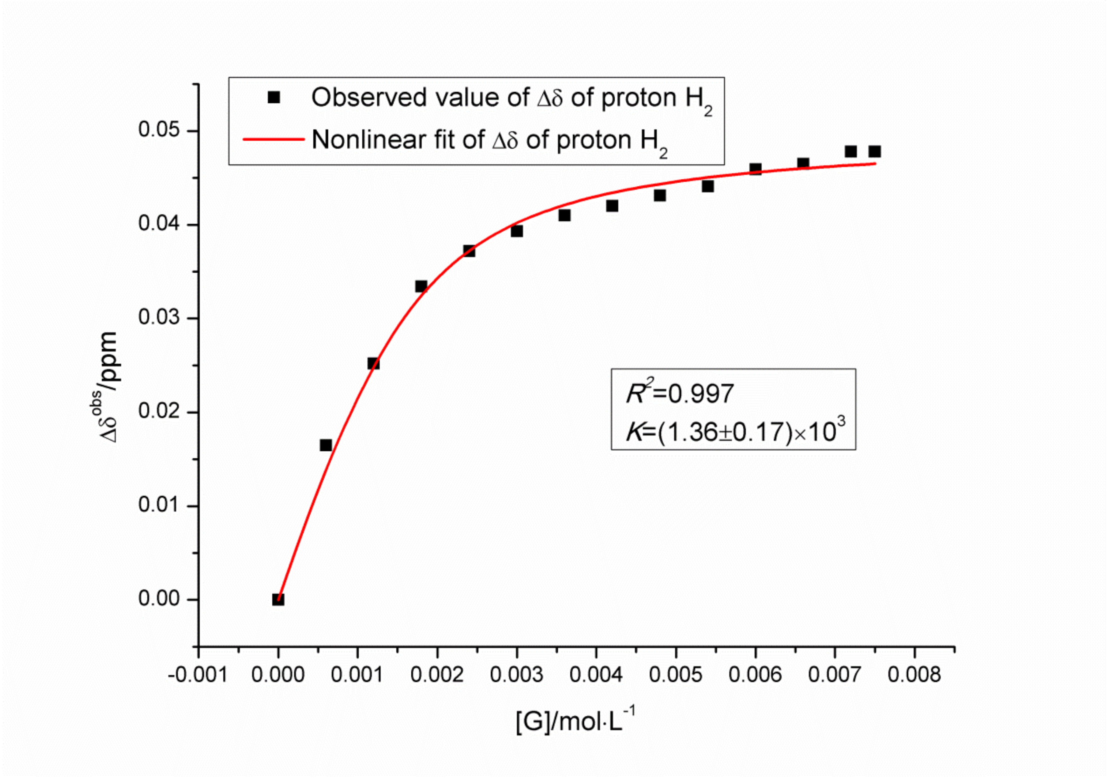


**Figure S60:** Plots of $\Delta\delta_{obs}$ (ppm) for the bridgehead proton $H_2$ of **H4** vs **G1** concentration in $CDCl_3$/acetone-$d_6$ 1:2 (v/v) at 298 K.

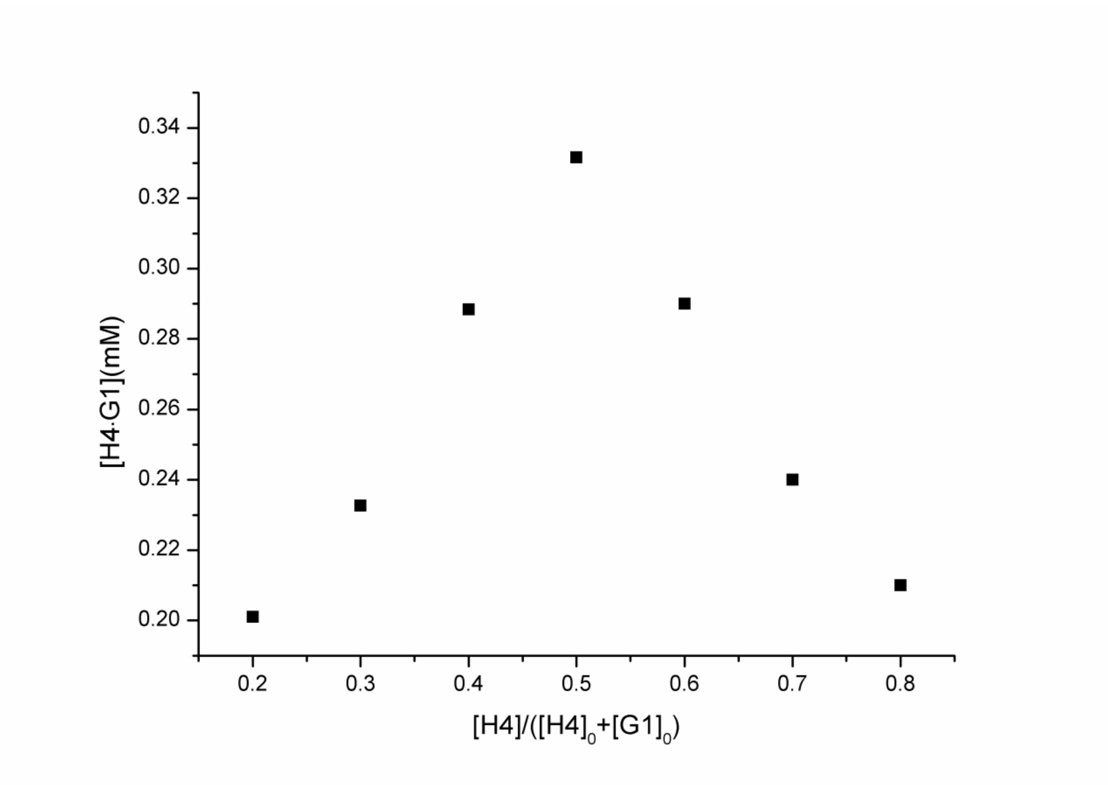


**Figure S61:** Job plot for the complexation of **H4** and **G1** in $CDCl_3$/acetone-$d_6$ 1:2 (v/v) at 298 K.

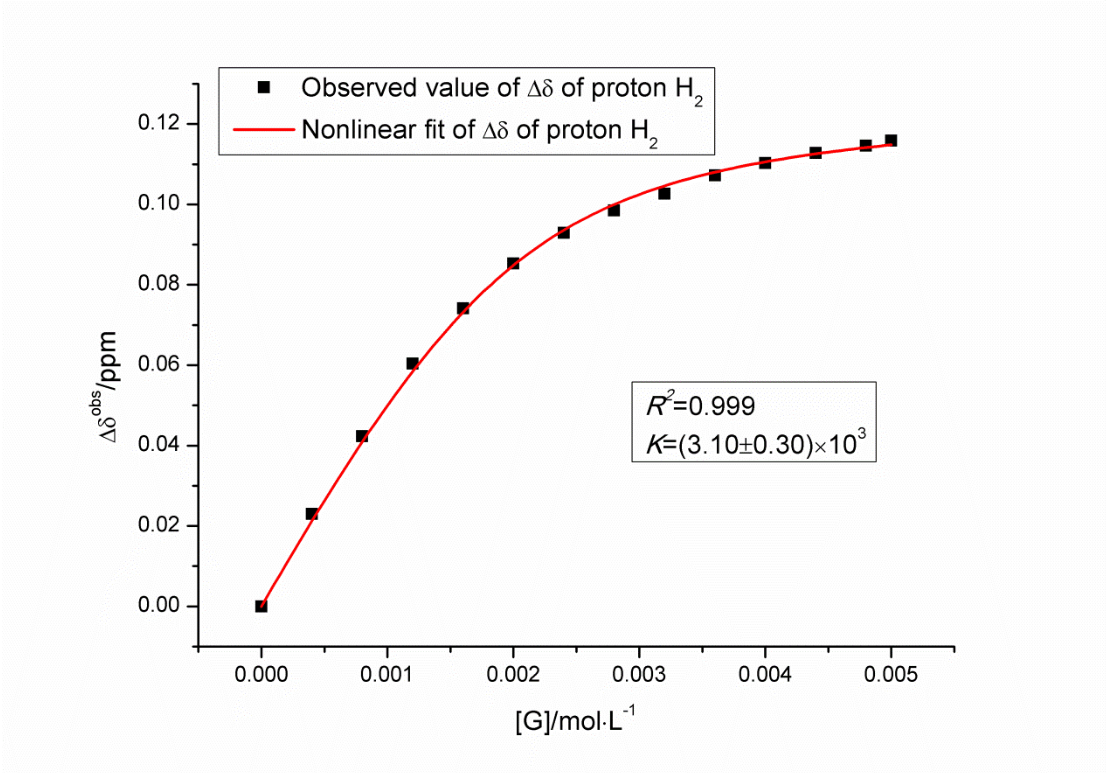


**Figure S62:** Plots of $\Delta\delta_{obs}$ (ppm) for the bridgehead proton $H_2$ of **H5** vs **G1** concentration in $CDCl_3$/acetone-$d_6$ 1:2 (v/v) at 298 K.

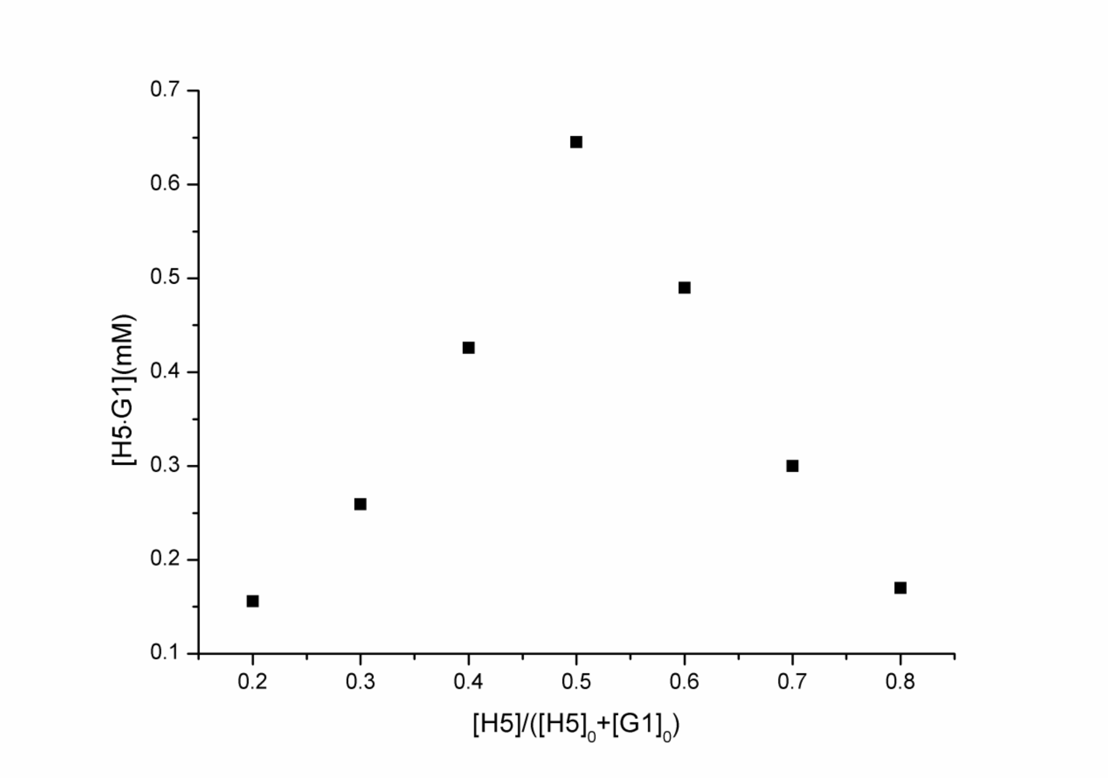


**Figure S63:** Job plot for the complexation of **H5** and **G1** in $CDCl_3$/acetone-$d_6$ 1:2 (v/v) at 298 K.

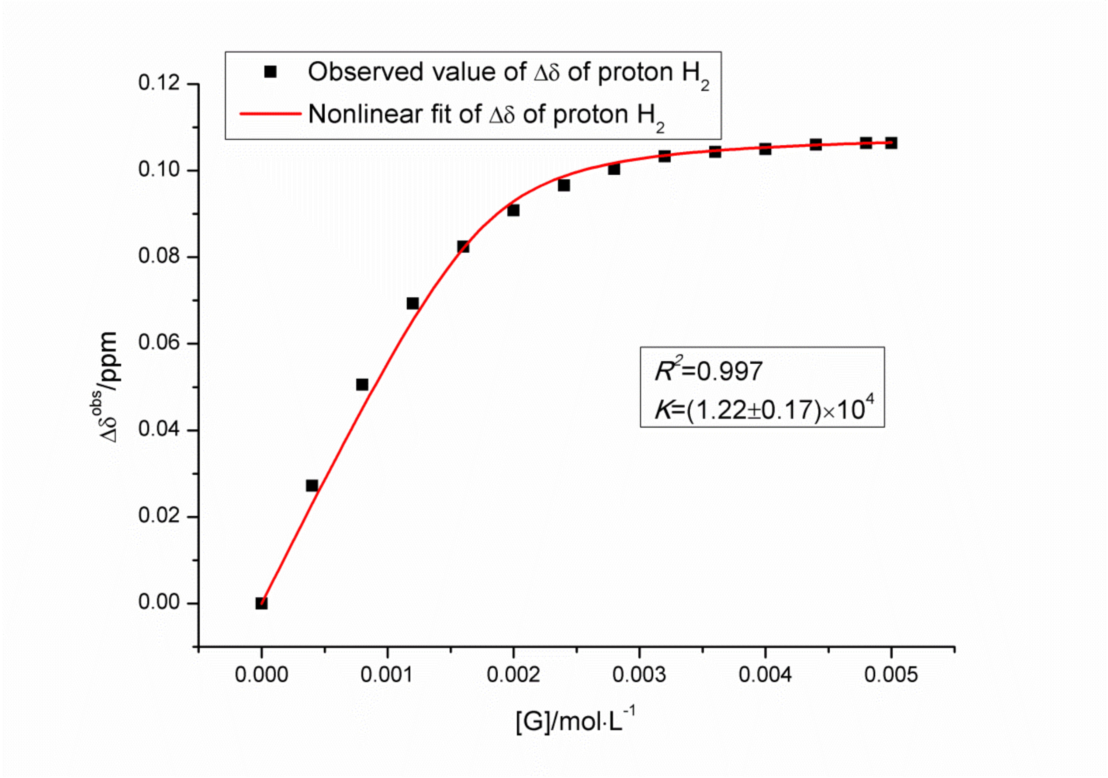


**Figure S64:** Plots of $\Delta\delta_{obs}$ (ppm) for the bridgehead proton $H_2$ of **H1** vs **G2** concentration in $CDCl_3$/acetone-$d_6$ 1:2 (v/v) at 298 K.

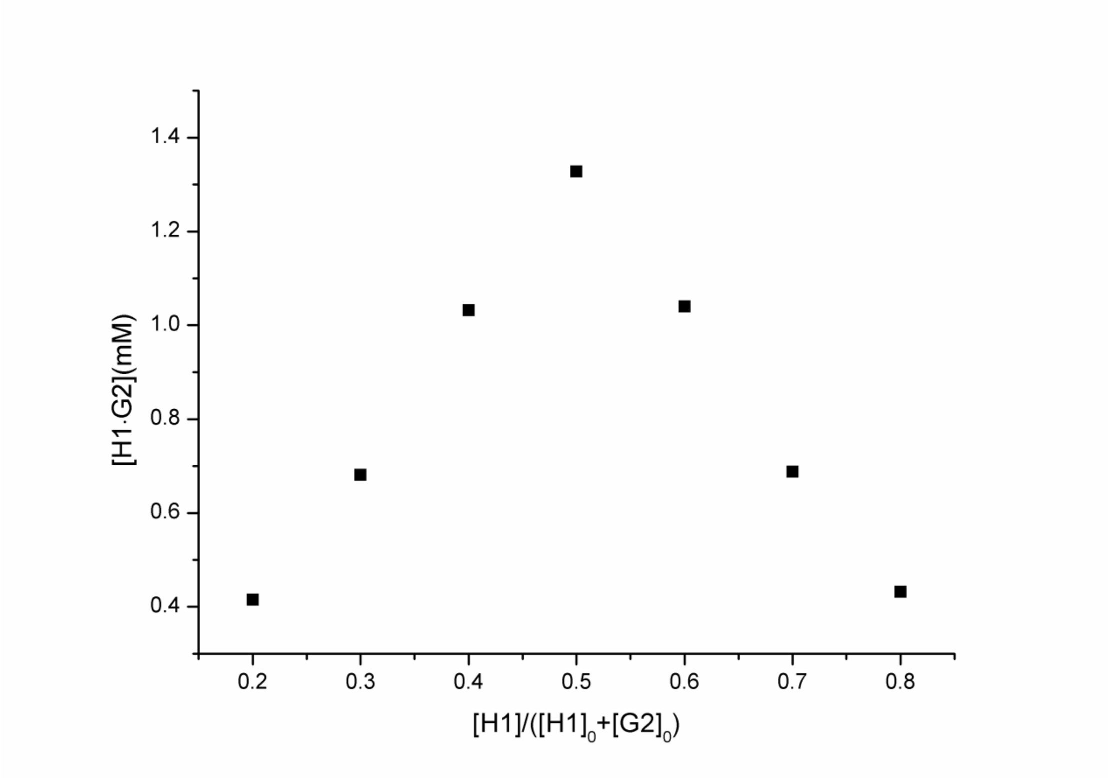


**Figure S65:** Job plot for the complexation of **H1** and **G2** in $CDCl_3$/acetone-$d_6$ 1:2 (v/v) at 298 K.

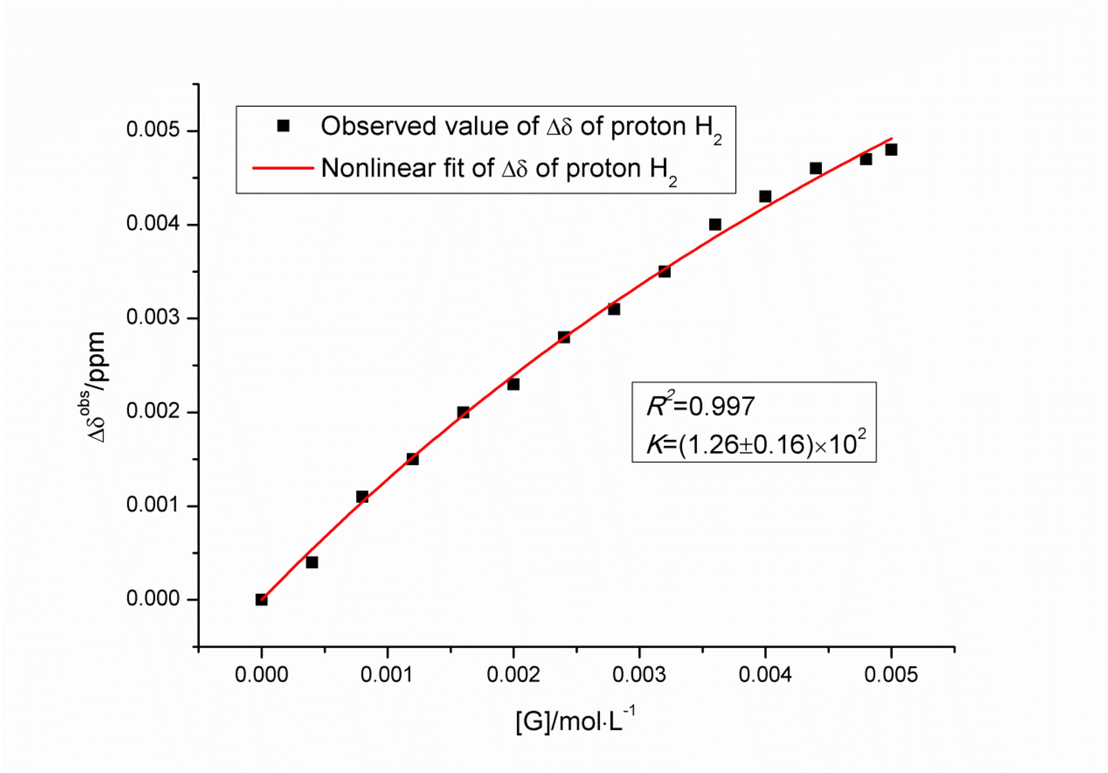


**Figure S66:** Plots of $\Delta\delta_{obs}$ (ppm) for the bridgehead proton $H_2$ of **H2** vs **G2** concentration in $CDCl_3$/acetone-$d_6$ 1:2 (v/v) at 298 K.

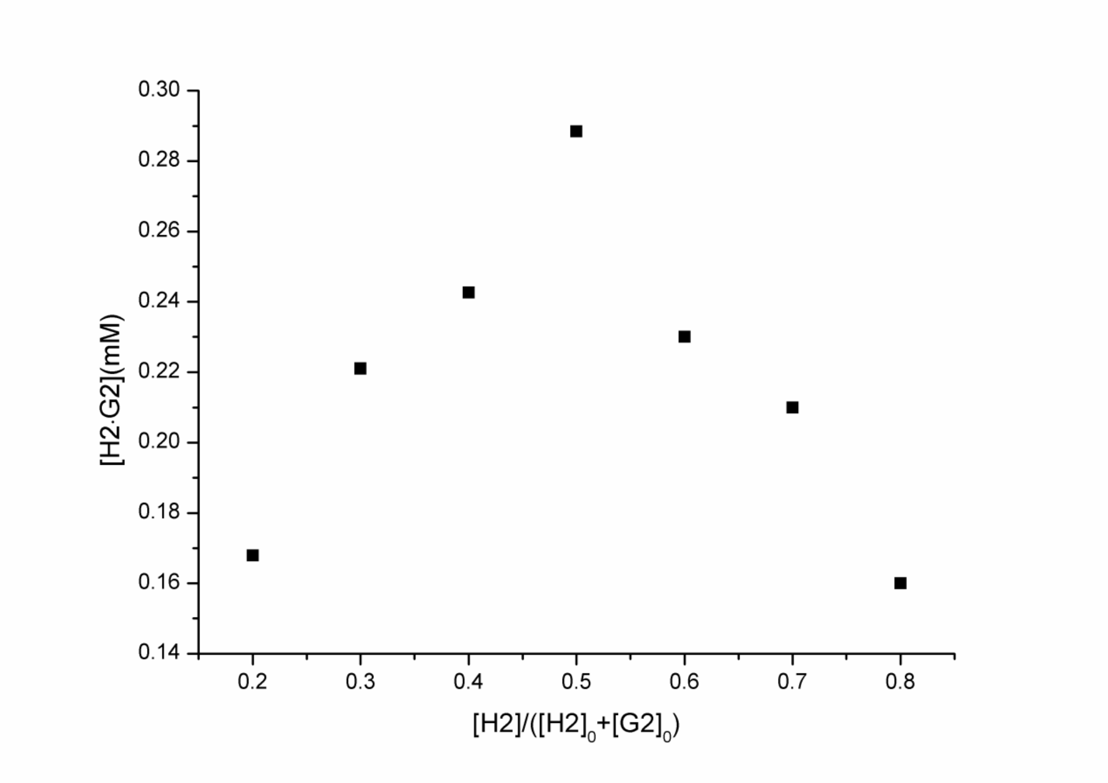


**Figure S67:** Job plot for the complexation of **H2** and **G2** in $CDCl_3$/acetone-$d_6$ 1:2 (v/v) at 298 K.

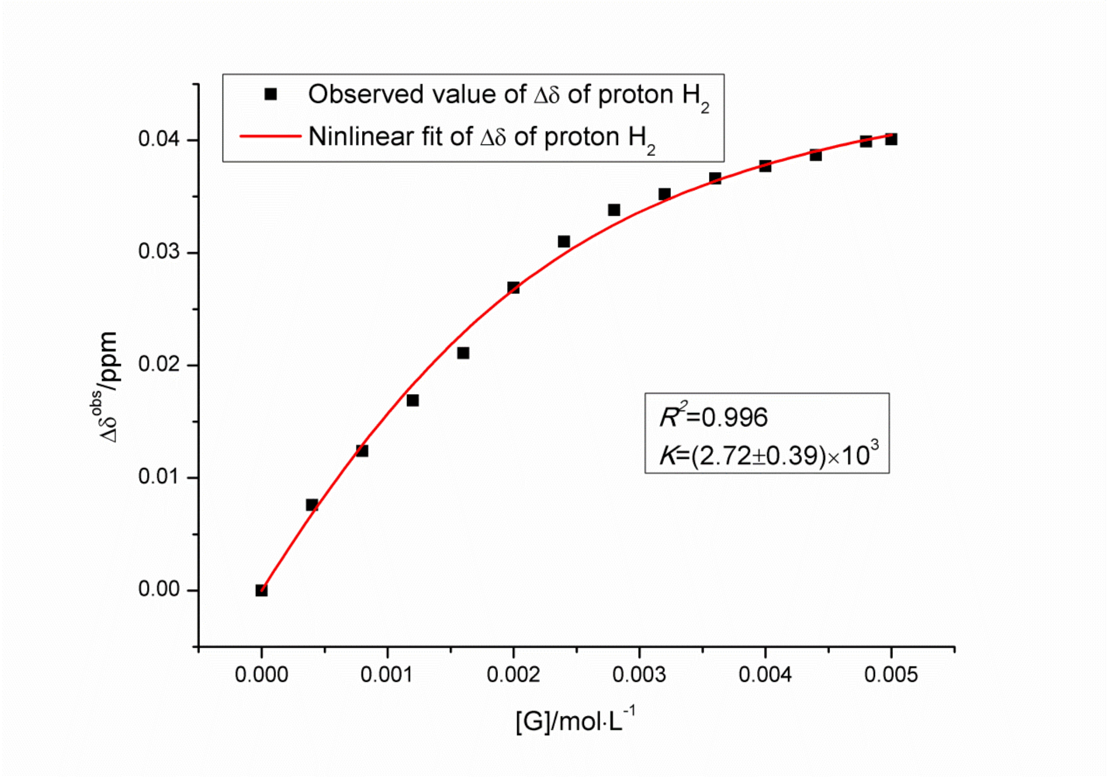


**Figure S68:** Plots of $\Delta\delta_{obs}$ (ppm) for the bridgehead proton $H_2$ of **H4** vs **G2** concentration in $CDCl_3$/acetone-$d_6$ 1:2 (v/v) at 298 K.

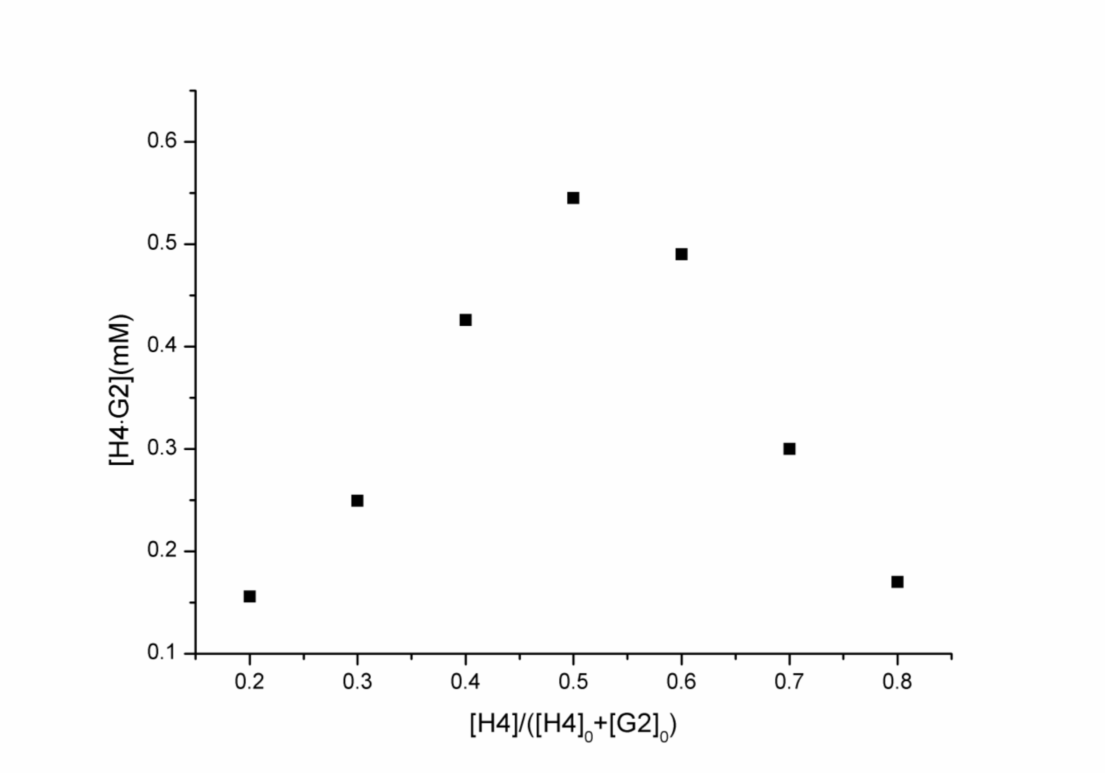


**Figure S69:** Job plot for the complexation of **H4** and **G2** in $CDCl_3$/acetone-$d_6$ 1:2 (v/v) at 298 K.

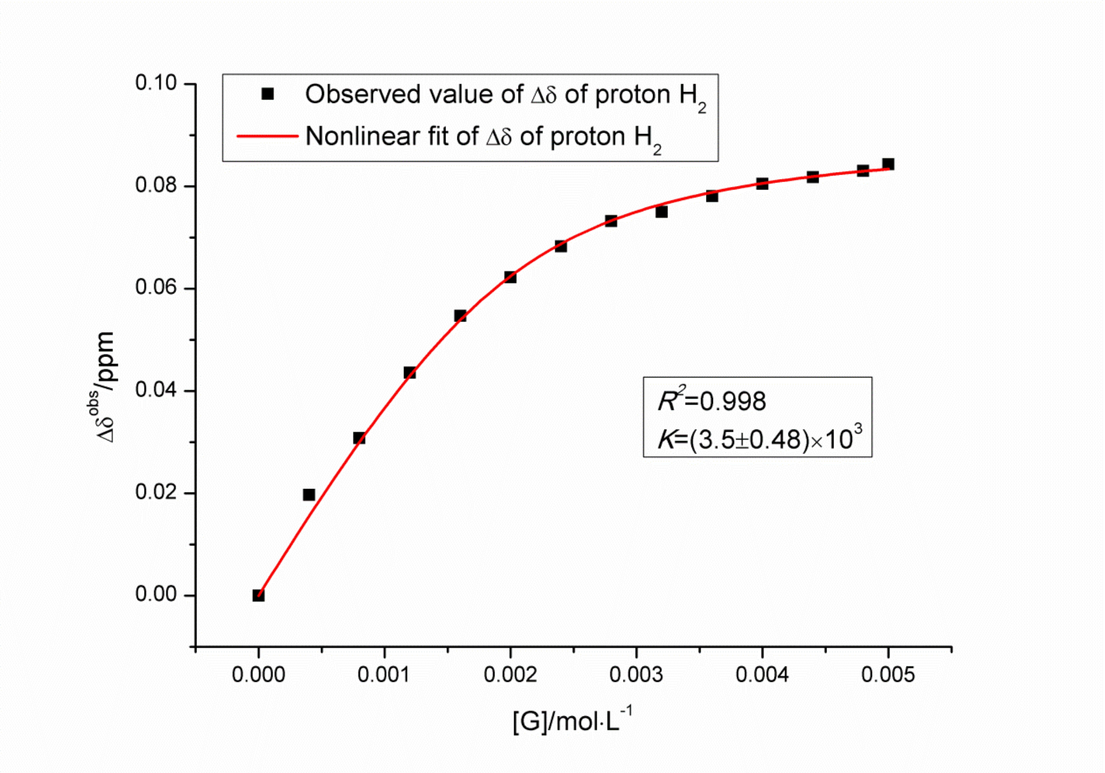


**Figure S70:** Plots of $\Delta\delta_{obs}$ (ppm) for the bridgehead proton $H_2$ of **H5** vs **G2** concentration in $CDCl_3$/acetone-$d_6$ 1:2 (v/v) at 298 K.

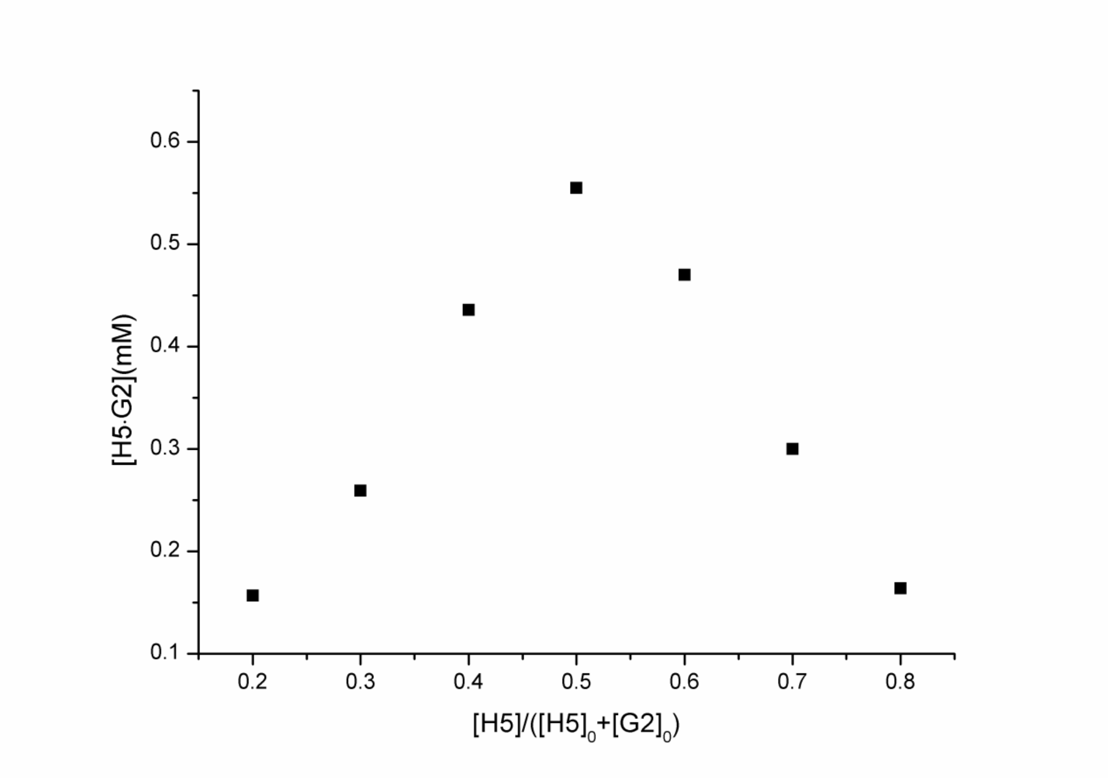


**Figure S71:** Job plot for the complexation of **H5** and **G2** in $CDCl_3$/acetone-$d_6$ 1:2 (v/v) at 298 K.

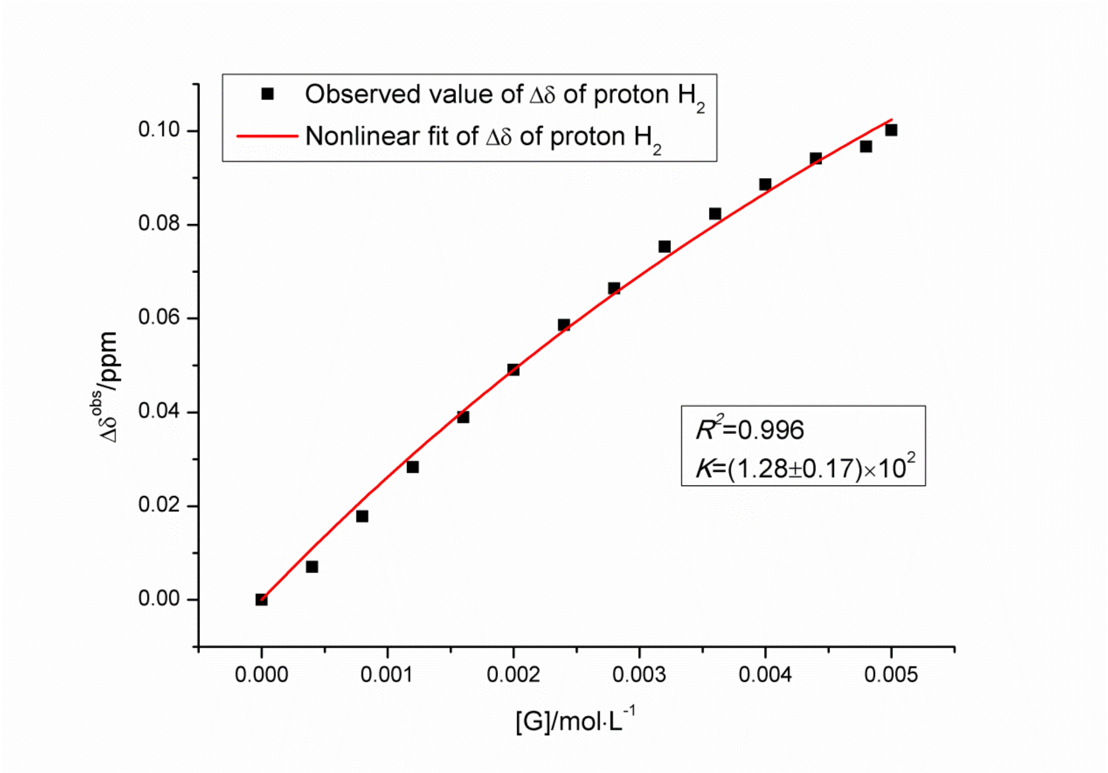


**Figure S72:** Plots of $\Delta\delta_{obs}$ (ppm) for the bridgehead proton $H_2$ of **H1** vs **G3** concentration in $CDCl_3$/acetone-$d_6$ 1:2 (v/v) at 298 K.

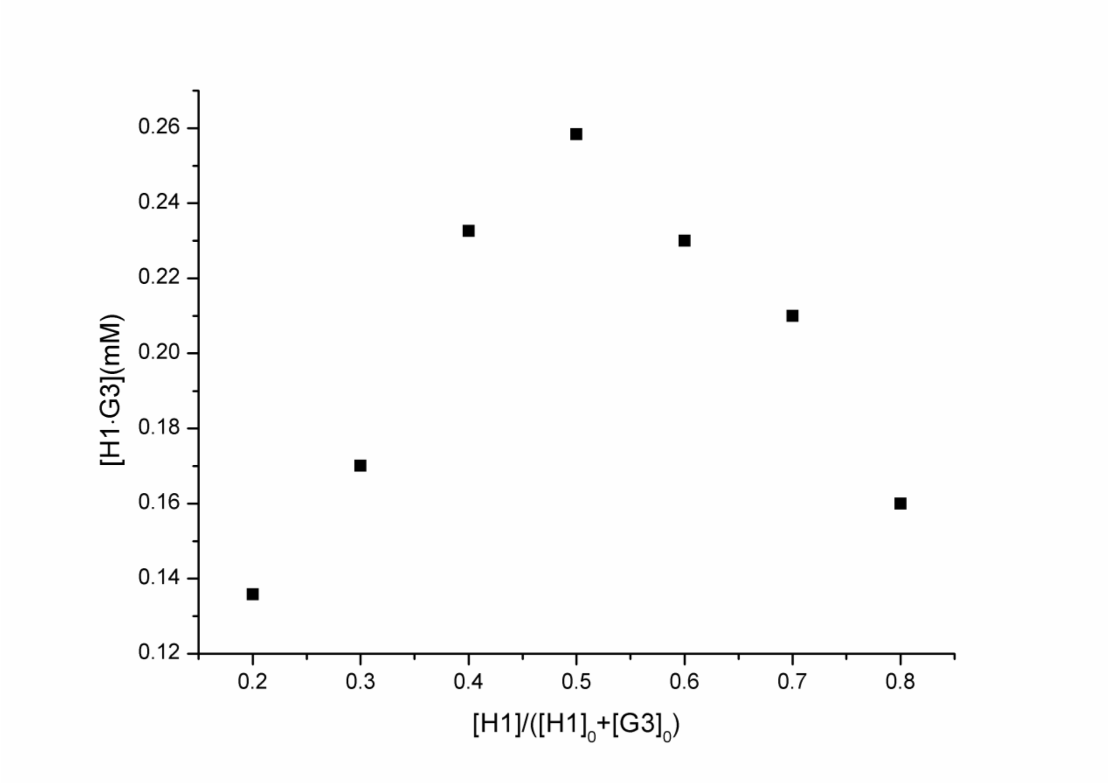


**Figure S73:** Job plot for the complexation of **H1** and **G3** in $CDCl_3$/acetone-$d_6$ 1:2 (v/v) at 298 K.

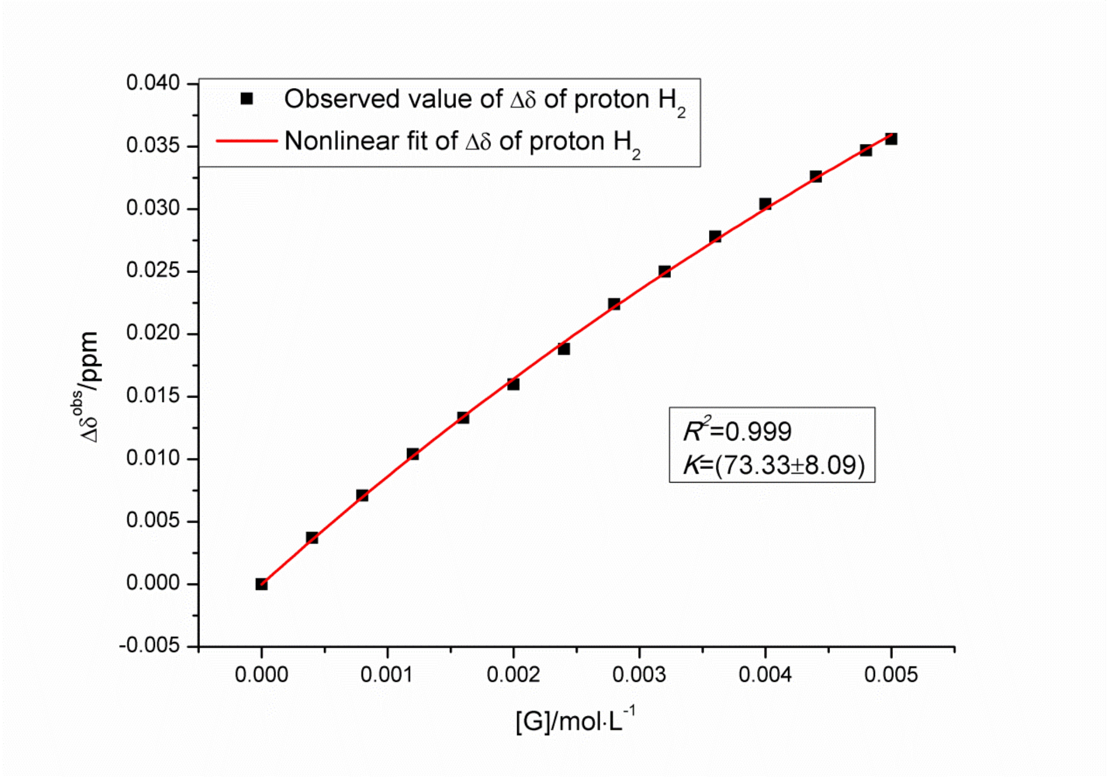


**Figure S74:** Plots of $\Delta\delta_{obs}$ (ppm) for the bridgehead proton $H_2$ of **H4** vs **G3** concentration in $CDCl_3$/acetone-$d_6$ 1:2 (v/v) at 298 K.

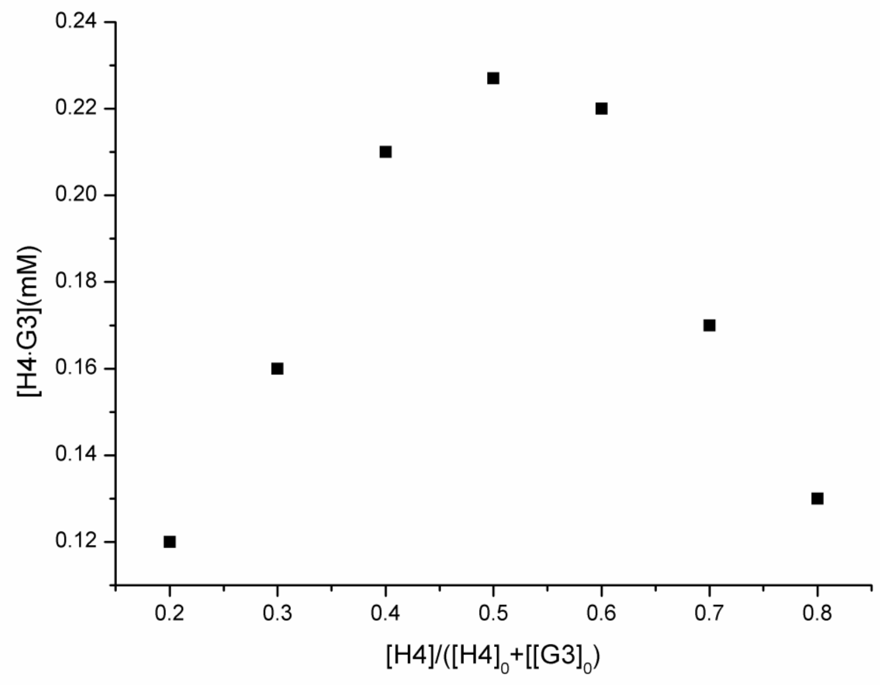


**Figure S75:** Job plot for the complexation of **H4** and **G3** in $CDCl_3$/acetone-$d_6$ 1:2 (v/v) at 298 K.

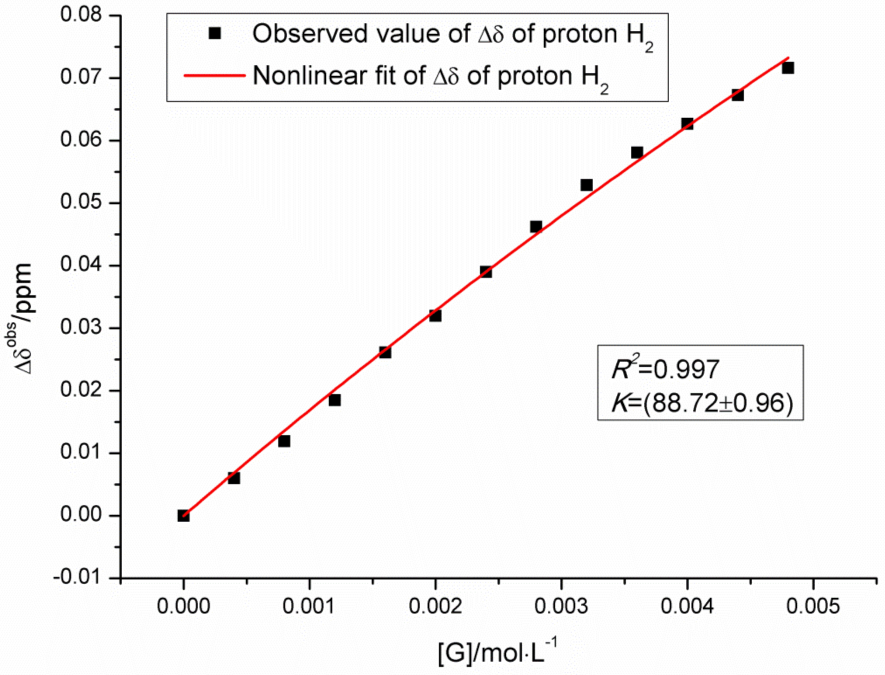


**Figure S76:** Plots of $\Delta\delta_{obs}$ (ppm) for the bridgehead proton $H_2$ of **H5** vs **G3** concentration in $CDCl_3$/acetone-$d_6$ 1:2 (v/v) at 298 K.

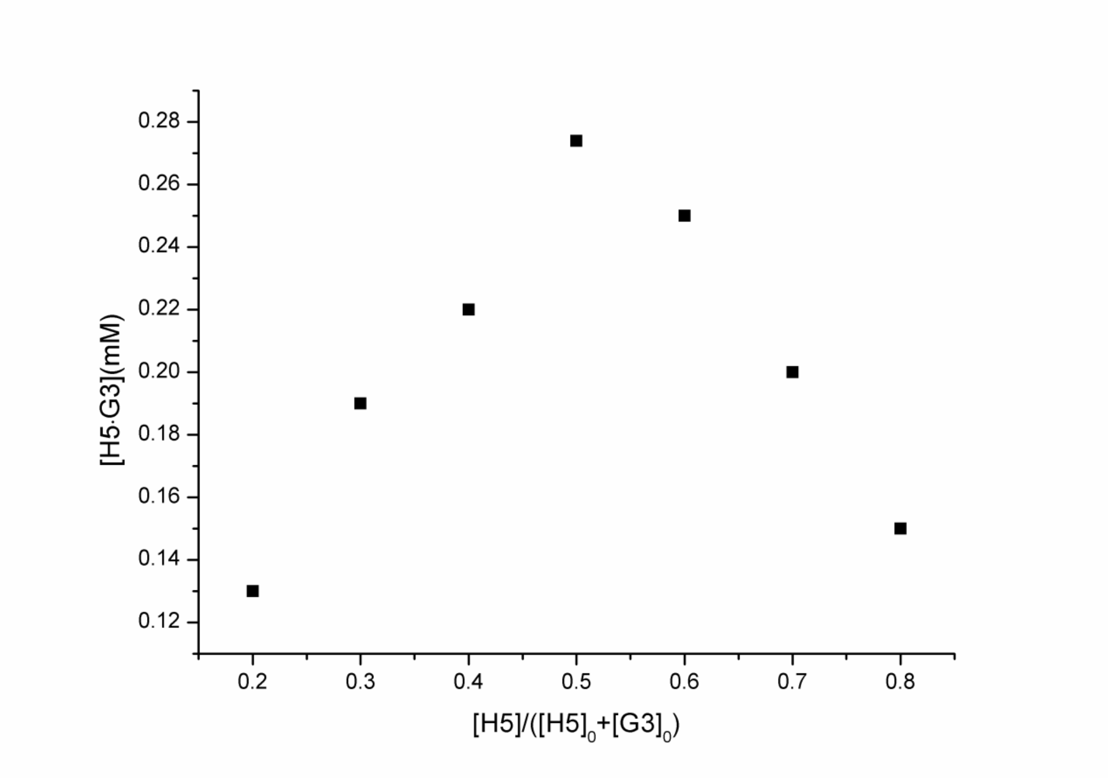


**Figure S77:** Job plot for the complexation of **H5** and **G3** in $CDCl_3$/acetone-$d_6$ 1:2 (v/v) at 298 K.

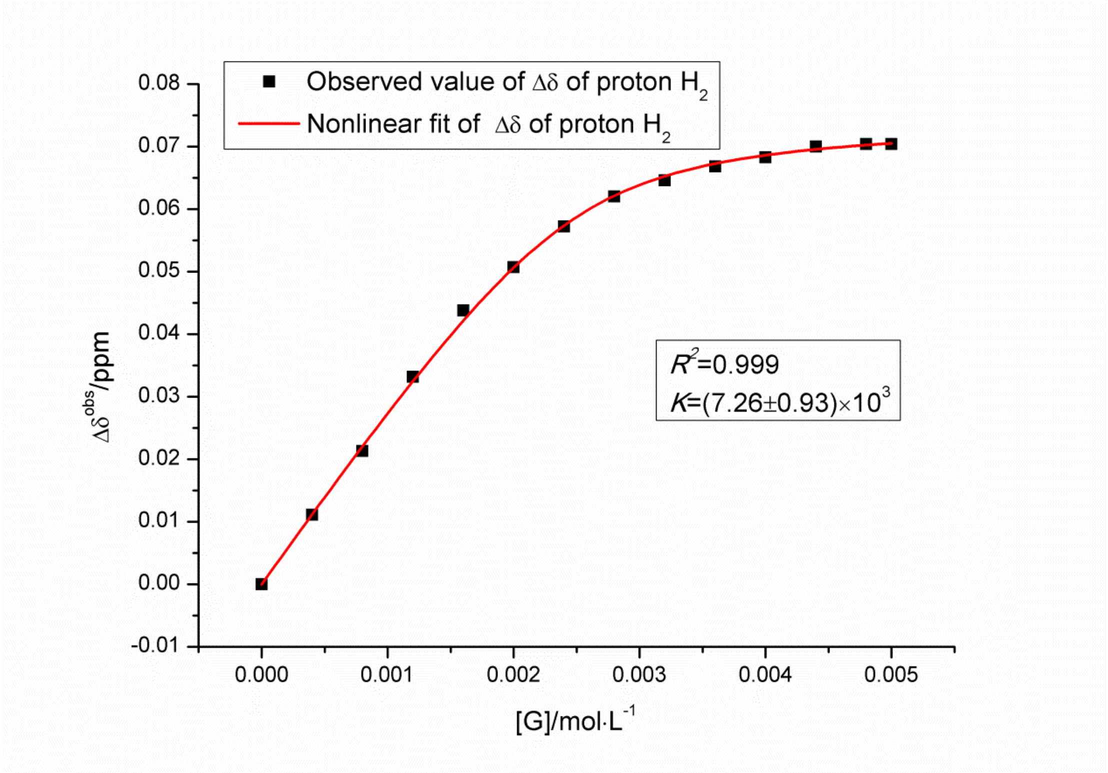


**Figure S78:** Plots of $\Delta\delta_{obs}$ (ppm) for the bridgehead proton $H_2$ of **H1** vs **G4** concentration in $CDCl_3$/acetone-$d_6$ 1:2 (v/v) at 298 K.

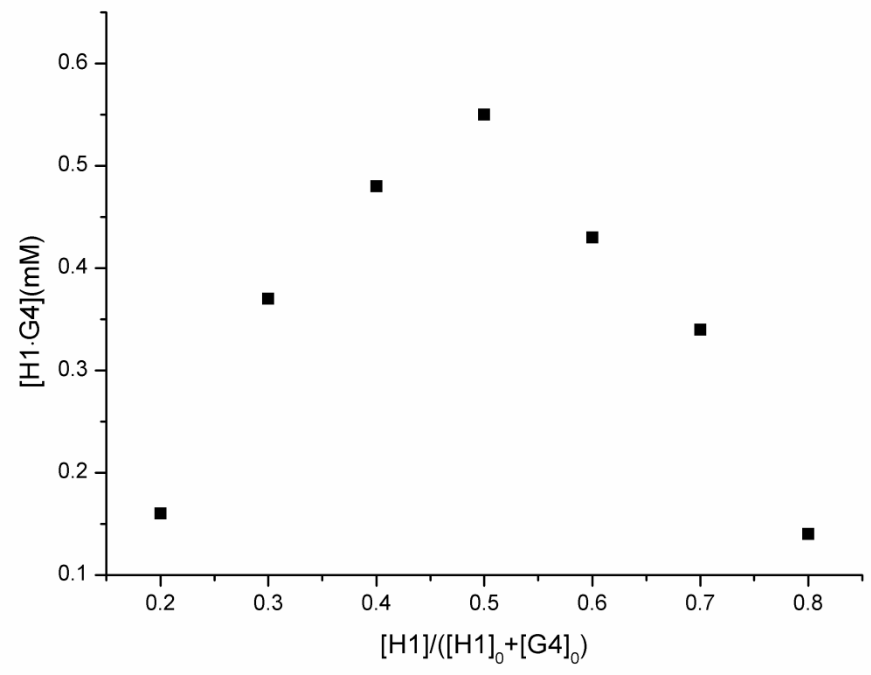


**Figure S79:** Job plot for the complexation of **H1** and **G4** in $CDCl_3$/acetone-$d_6$ 1:2 (v/v) at 298 K.

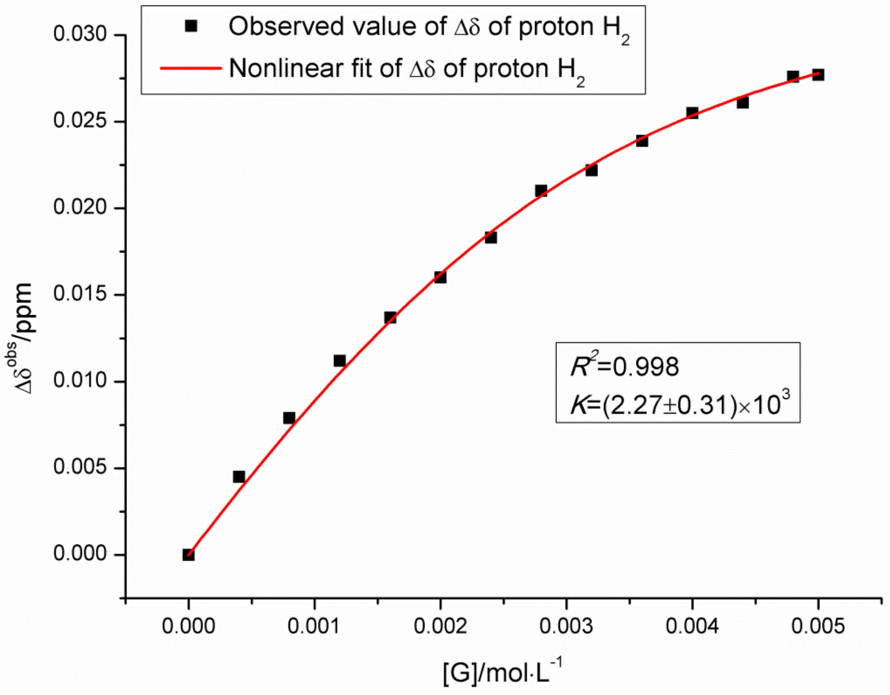


**Figure S80:** Plots of $\Delta\delta_{obs}$ (ppm) for the bridgehead proton $H_2$ of **H4** vs **G4** concentration in $CDCl_3$/acetone-$d_6$ 1:2 (v/v) at 298 K.

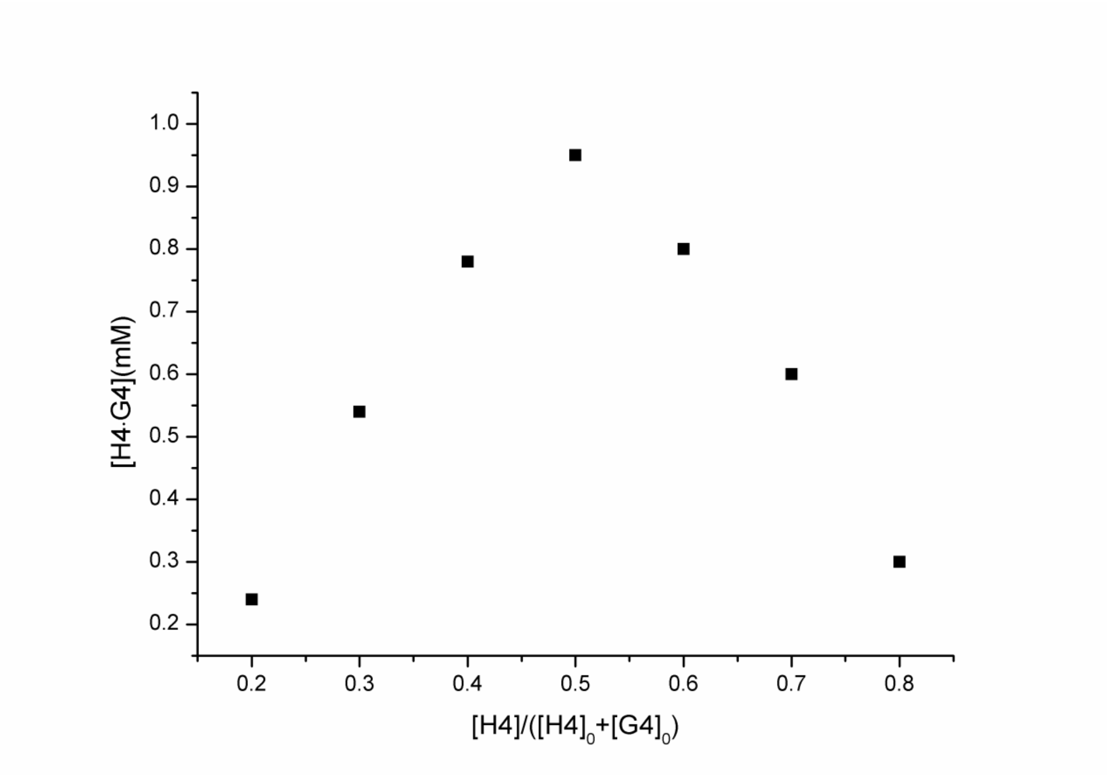


**Figure S81:** Job plot for the complexation of **H4** and **G4** in $CDCl_3$/acetone-$d_6$ 1:2 (v/v) at 298 K.

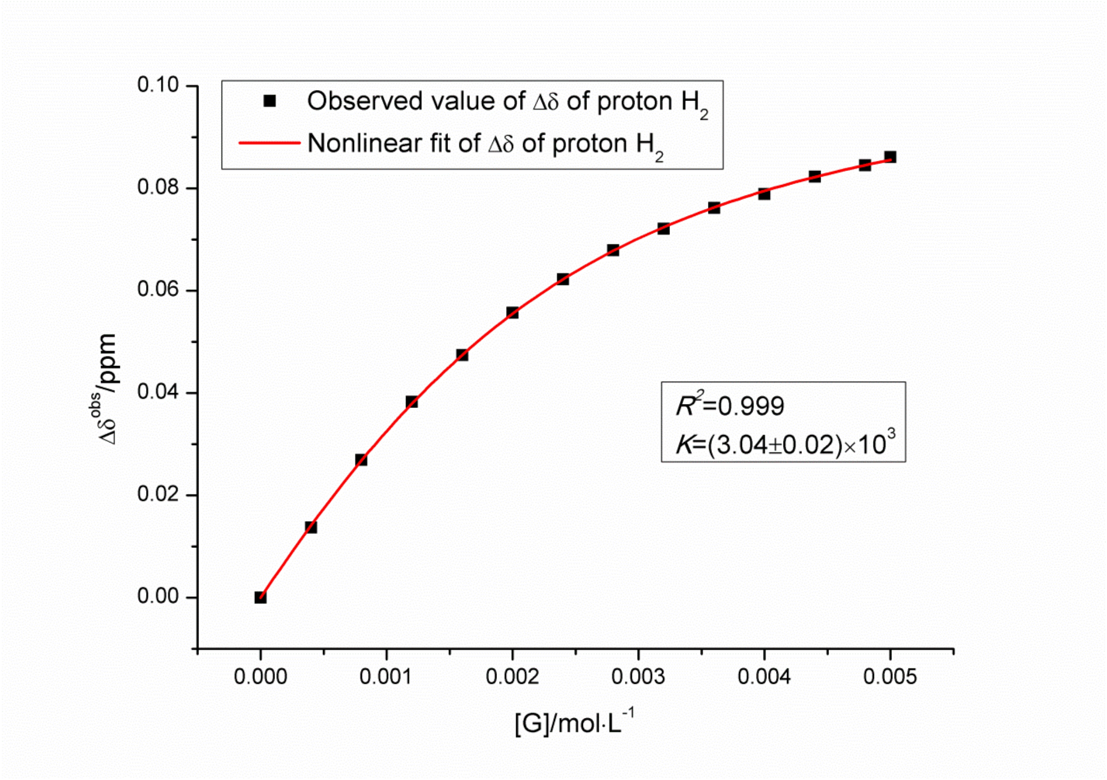


**Figure S82:** Plots of $\Delta\delta_{obs}$ (ppm) for the bridgehead proton $H_2$ of **H5** vs **G4** concentration in $CDCl_3$/acetone-$d_6$ 1:2 (v/v) at 298 K.

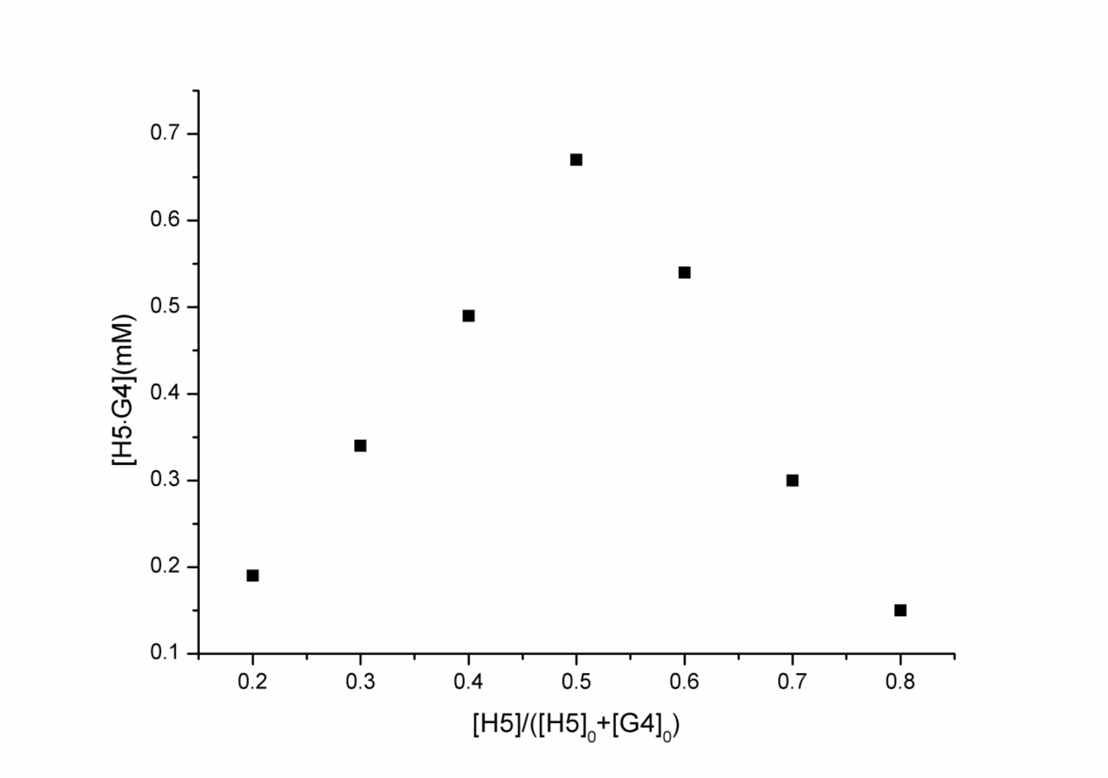


**Figure S83:** Job plot for the complexation of **H5** and **G4** in $CDCl_3$/acetone-$d_6$ 1:2 (v/v) at 298 K.

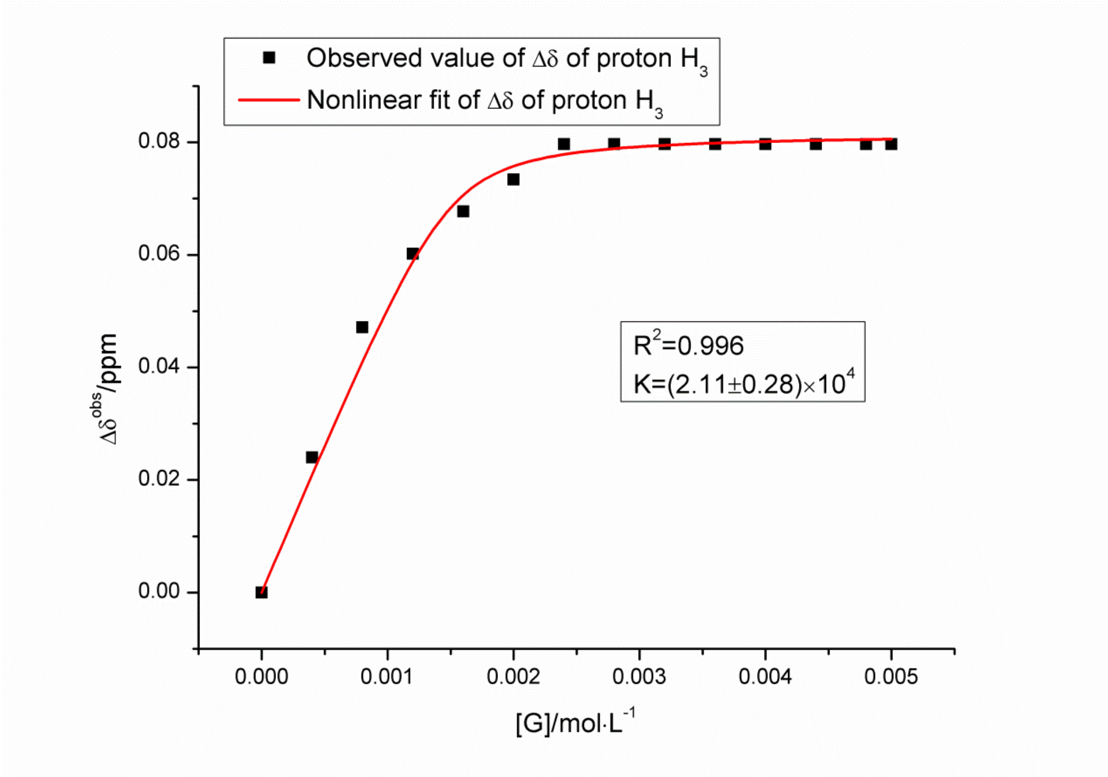


**Figure S84:** Plots of $\Delta\delta_{obs}$ (ppm) for the bridgehead proton $H_3$ of **H1** vs **G4** concentration in $CD_2Cl_2$ at 298 K.

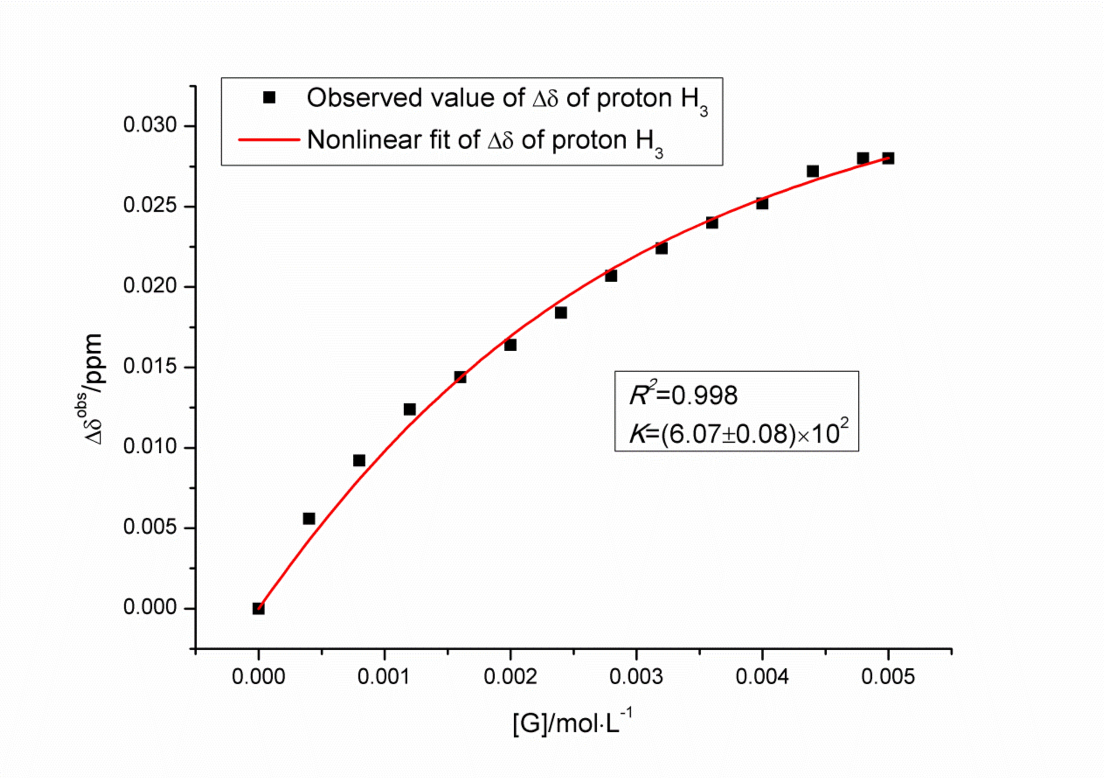


**Figure S85:** Plots of $\Delta\delta_{obs}$ (ppm) for the bridgehead proton $H_3$ of **H2** vs **G4** concentration in $CD_2Cl_2$ at 298 K.

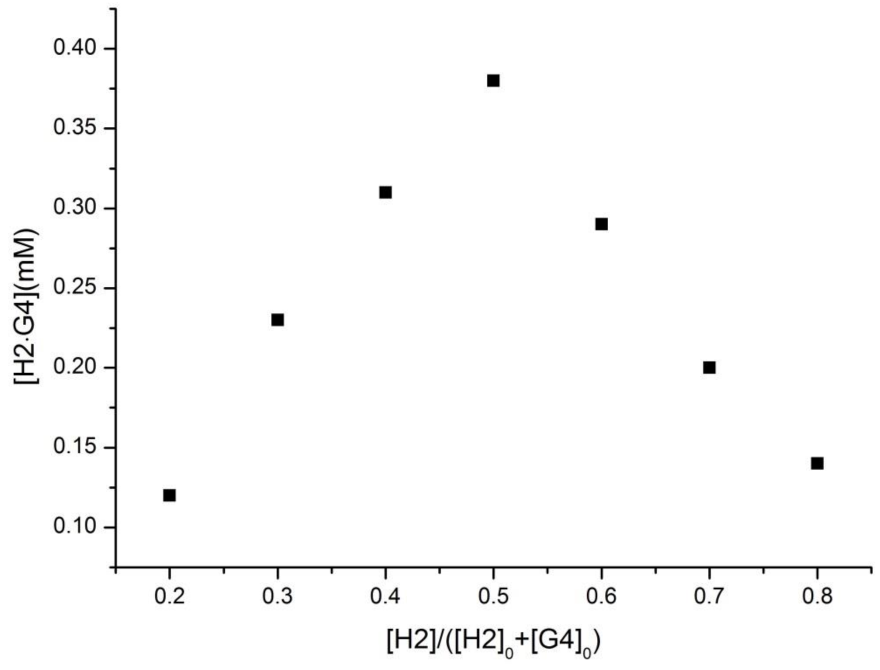


**Figure S86:** Job plot for the complexation of **H2** and **G4** in $CD_2Cl_2$ at 298 K.

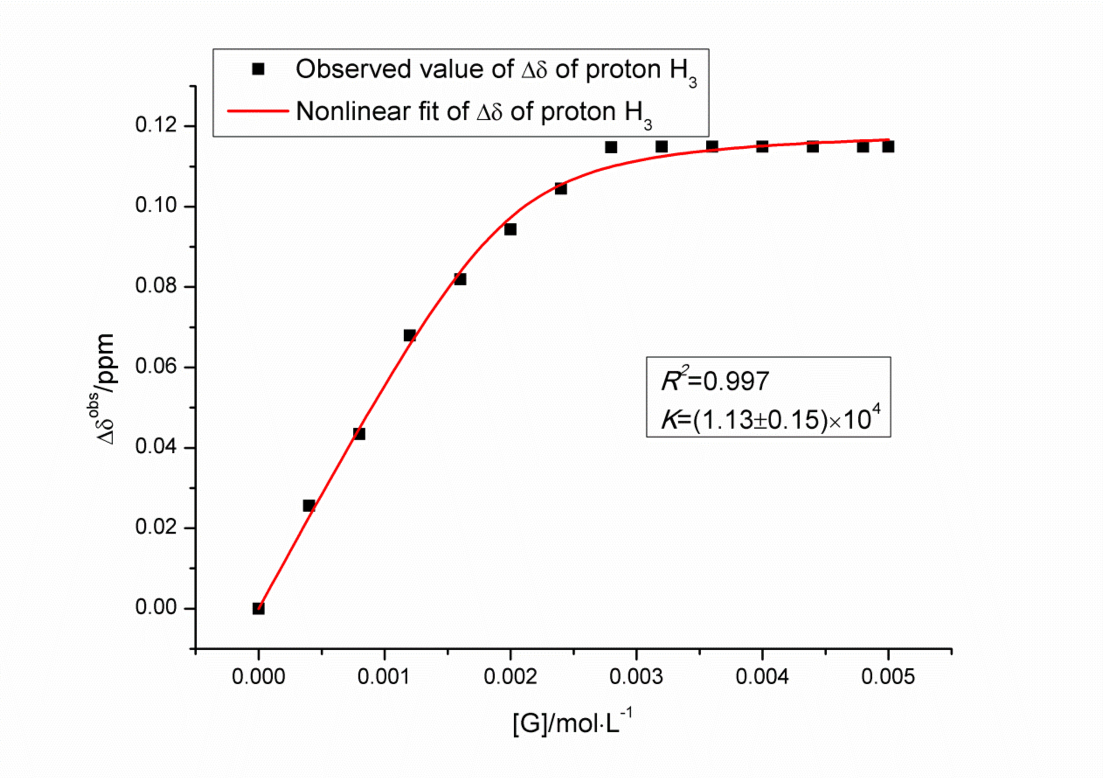


**Figure S87:** Plots of $\Delta\delta_{obs}$ (ppm) for the bridgehead proton $H_3$ of **H4** vs **G4** concentration in $CD_2Cl_2$ at 298 K.

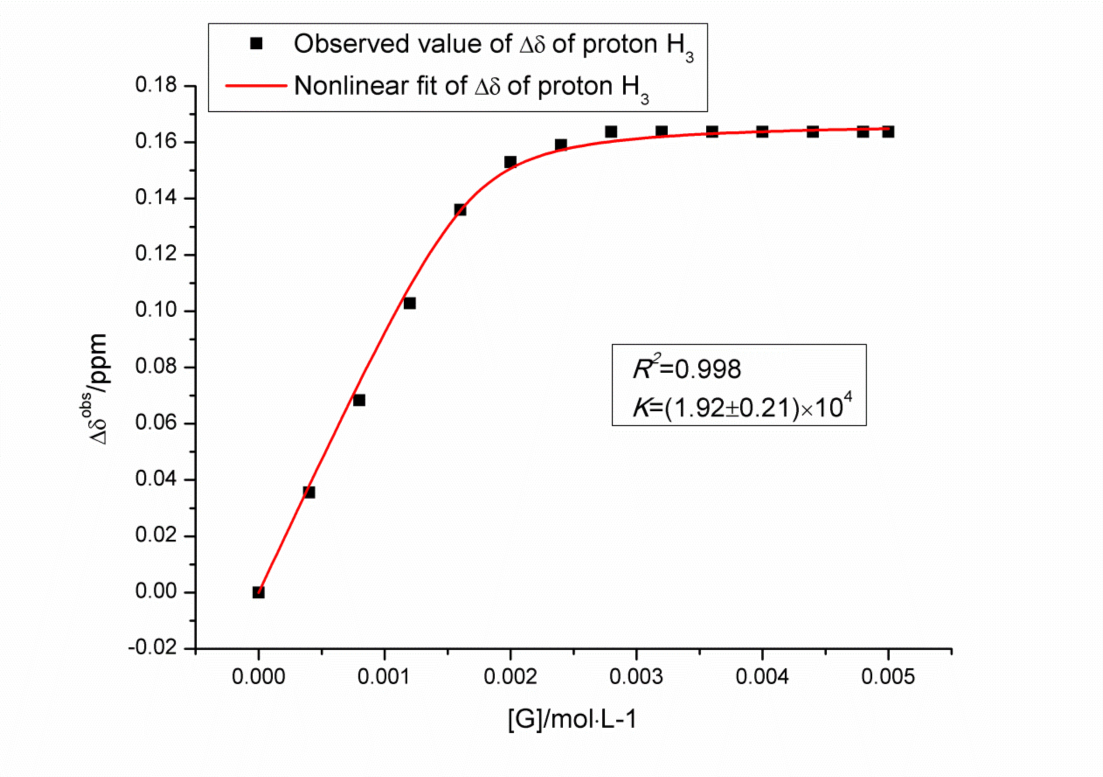


**Figure S88:** Plots of $\Delta\delta_{obs}$ (ppm) for the bridgehead proton $H_3$ of **H5** vs **G4** concentration in $CD_2Cl_2$ at 298 K.

## 7. Crystal structures and crystal data

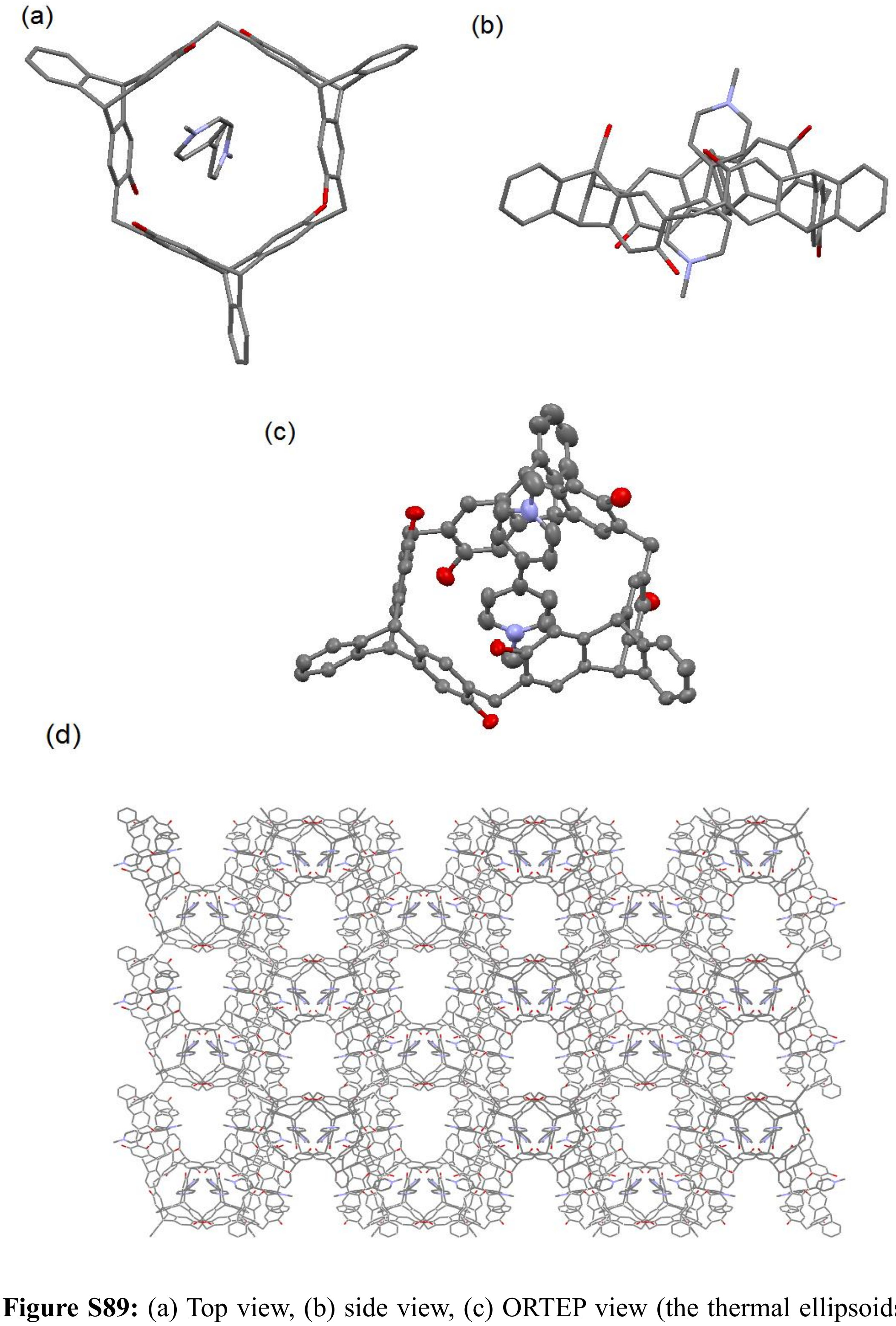

**Figure S89:** (a) Top view, (b) side view, (c) ORTEP view (the thermal ellipsoids are displayed at 50% probability), (d) packing mode of complex **H1·G1**. Solvent molecules, $PF_6^-$ counteranions and hydrogen atoms were omitted for clarity.

**Table S1:** Crystal data and structure refinement for complex **H1·G1** (CCDC 1909906).

| | |
|---|---|
| Empirical formula | $C_{75}H_{56}F_{12}N_2O_6P_2$ |
| Formula weight | 1371.15 |
| Temperature | 169.99(10) K |
| Wavelength | 1.54184 Å |
| Crystal system | Tetragonal |
| Space group | P 4/n |
| Unit cell dimensions | a = 30.69180(10) Å α= 90°<br>b = 30.69180(10) Å β= 90°<br>c = 20.24310(10) Å γ = 90° |
| Volume | 19068.73(16) Å$^3$ |
| Z | 8 |
| Density (calculated) | 0.955 Mg/m$^3$ |
| Absorption coefficient | 0.952 mm$^{-1}$ |
| F(000) | 5648 |
| Crystal size | 0.245 x 0.231 x 0.212 mm$^3$ |
| Theta range for data collection | 2.183 to 75.438°. |
| Index ranges | -38<=h<=38, -37<=k<=32, -24<=l<=24 |
| Reflections collected | 93931 |
| Independent reflections | 19235 [R(int) = 0.0310] |
| Completeness to theta = 67.684° | 99.8 % |
| Absorption correction | Semi-empirical from equivalents |
| Max. and min. transmission | 1.00000 and 0.84185 |
| Refinement method | Full-matrix least-squares on F$^2$ |
| Data / restraints / parameters | 19235 / 0 / 914 |
| Goodness-of-fit on F$^2$ | 1.095 |
| Final R indices [I>2sigma(I)] | R1 = 0.0946, wR2 = 0.2965 |
| R indices (all data) | R1 = 0.1058, wR2 = 0.3073 |
| Extinction coefficient | n/a |
| Largest diff. peak and hole | 0.930 and -0.577 e.Å$^{-3}$ |

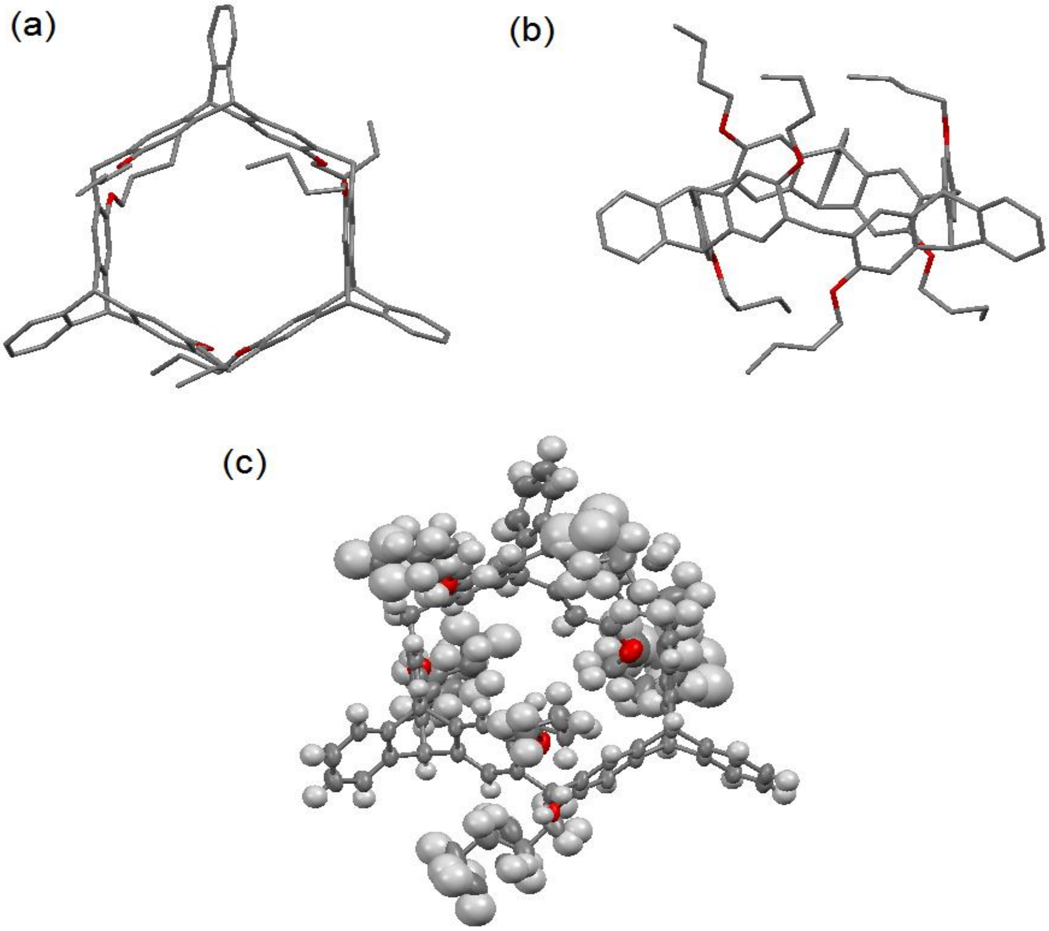


**Figure S90:** (a) Top view, (b) side view, and (c) ORTEP view (the thermal ellipsoids are displayed at 50% probability) of **H3**. Solvent molecules and hydrogen atoms were omitted for clarity.

**Table S2:** Crystal data and structure refinement for **H3** (CCDC 1909907).

| | |
|---|---|
| Empirical formula | $C_{87}H_{90}O_6$ |
| Formula weight | 1231.58 |
| Temperature | 169.99(10) K |
| Crystal system | monoclinic |
| Space group | $P2_1/n$ |
| Unit cell dimensions | a = 23.3453(2) Å α= 90°<br>b = 17.96290(10) Å β= 115.9080°<br>c = 23.3923(2) Å γ = 90° |
| Volume | 8823.65(14) Å$^3$ |
| Z | 4 |

| | |
|---|---|
| Density (calculated) | 0.927 Mg/m$^3$ |
| Absorption coefficient | 0.440 mm$^{-1}$ |
| F(000) | 2640 |
| Crystal size | 0.35 x 0.35 x 0.15 mm$^3$ |
| Theta range for data collection | 6.47 to 150.802°. |
| Index ranges | -24<=h<=29, -21<=k<=21, -29<=l<=23 |
| Reflections collected | 72701 |
| Independent reflections | 17519 [R(int) = 0.0252] |
| Completeness to theta = 67.684° | 99.8 % |
| Absorption correction | Semi-empirical from equivalents |
| Max. and min. transmission | 1.00000 and 0.84185 |
| Refinement method | Full-matrix least-squares on F$^2$ |
| Data / restraints / parameters | 17519 / 418 / 962 |
| Goodness-of-fit on F$^2$ | 1.995 |
| Final R indices [I>2sigma(I)] | R1 = 0.0710, wR2 = 0.2370 |
| R indices (all data) | R1 = 0.0790, wR2 = 0.2459 |
| Extinction coefficient | n/a |
| Largest diff. peak and hole | 0.64 and -0.38 e.Å$^{-3}$ |

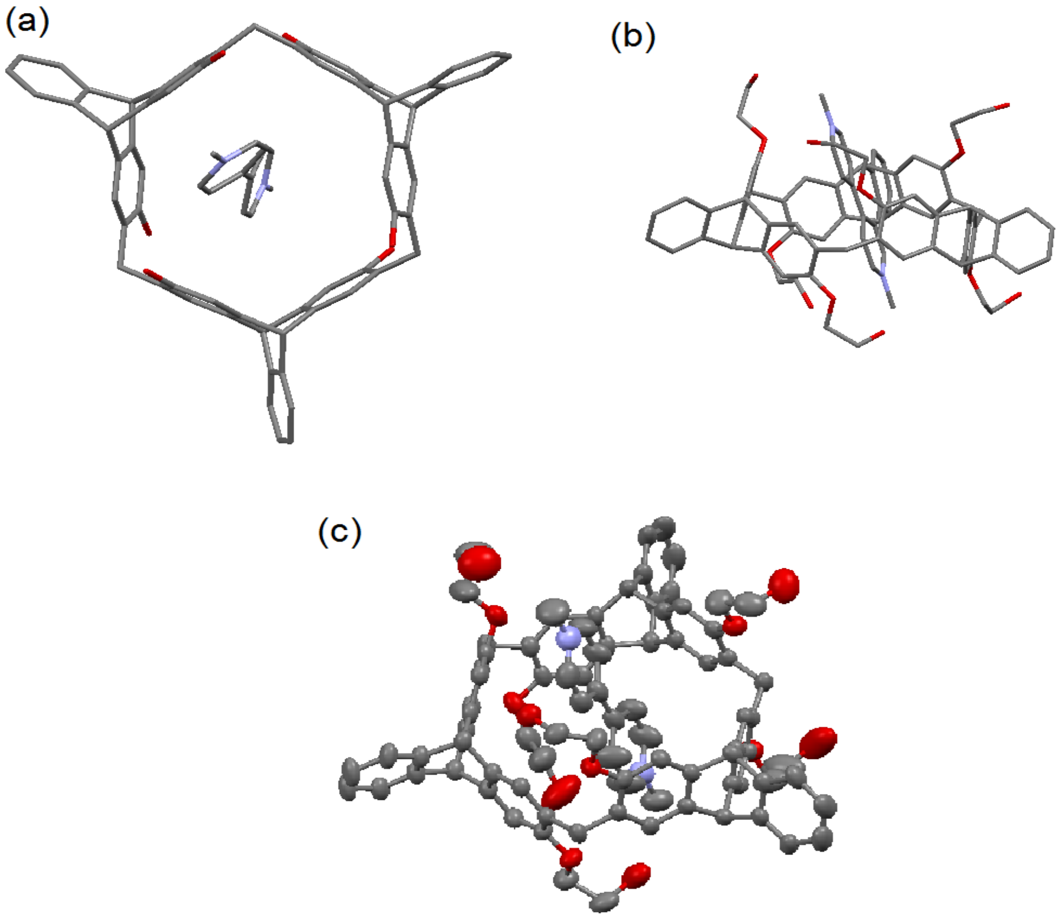


**Figure S91:** (a) Top view, (b) side view, and (c) ORTEP view (the thermal ellipsoids are displayed at 50% probability) of complex **H5·G1**. Solvent molecules, $PF_6^-$ counteranions and hydrogen atoms were omitted for clarity.

**Table S3:** Crystal data and structure refinement for complex **H5·G1** (CCDC 1909908).

| | |
|---|---|
| Empirical formula | $C_{87}H_{80}F_{12}N_2O_{12}P_2$ |
| Formula weight | 1635.47 |
| Temperature | 169.99(10) K |
| Crystal system | monoclinic |
| Space group | $P2_1/n$ |
| Unit cell dimensions | a = 12.58890(10) Å α= 90°<br>b = 23.8485(2) Å β= 94.4480(10)°<br>c = 28.2505(2)Å γ = 90° |
| Volume | 8456.00(12) $Å^3$ |
| Z | 4 |

| | |
|---|---|
| Density (calculated) | 1.285 $Mg/m^3$ |
| Absorption coefficient | 1.211 $mm^{-1}$ |
| F(000) | 3400.0 |
| Crystal size | 0.2 x 0.1 x 0.05 $mm^3$ |
| Theta range for data collection | 4.856 to 151.66°. |
| Index ranges | -15<=h<=15, -29<=k<=27, -32<=l<=34 |
| Reflections collected | 243913 |
| Independent reflections | 32345 [R(int) = 0.0579] |
| Completeness to theta = 67.684° | 99.8 % |
| Absorption correction | Semi-empirical from equivalents |
| Max. and min. transmission | 1.00000 and 0.84185 |
| Refinement method | Full-matrix least-squares on $F^2$ |
| Data / restraints / parameters | 32345/241/2148 |
| Goodness-of-fit on $F^2$ | 1.643 |
| Final R indices [I>2sigma(I)] | R1 = 0.0783, wR2 = 0.2105 |
| R indices (all data) | R1 = 0.0901, wR2 = 0.2189 |
| Extinction coefficient | n/a |
| Largest diff. peak and hole | 0.98 and -0.52 e.$Å^{-3}$ |

## 8. DFT calculations for complexation between hosts and guests

Molecular models of free host, free guests and the complexes were constructed by referring to the single crystal or calculated structures that involved in the related interaction modes. To make sure of the accuracy, density functional theory (DFT) calculations based on the B3LYP/6-31G level of theory were carried using the Gaussian 09 program package. The actual counterions were not included in the calculations.

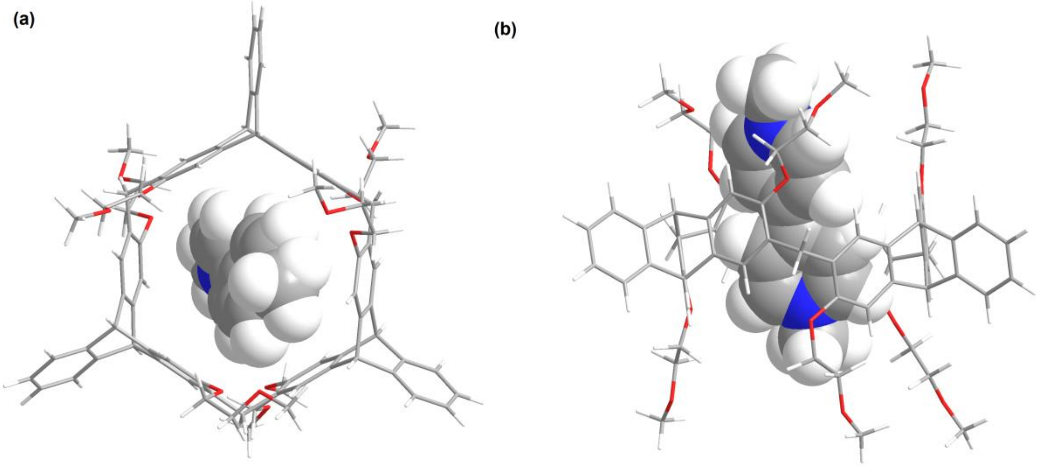


**Figure S92:** Calculated structure of the complex **H4·G1** at the B3LYP/6-31G level. (a) Top view and (b) side view of the complex.

The atomic coordinates of **H4·G1**:

| | | | |
|---|---|---|---|
| O | -4.77370200 | 1.42591000 | 2.02105100 |
| C | -5.89638700 | 2.02421300 | 2.78077600 |
| O | 2.82455500 | 3.69279800 | -2.33459100 |
| C | 2.89603100 | 4.01241900 | -3.76300900 |
| O | 2.62601700 | 3.57764600 | 3.01751200 |
| C | 2.77962300 | 3.58990300 | 4.47997700 |
| O | 2.80634900 | -4.13387800 | -1.85215100 |
| C | 3.76396900 | -4.86920000 | -2.69034100 |
| O | 0.85176300 | -4.18512500 | 3.22952800 |
| C | 0.34303100 | -4.02342500 | 4.59028700 |
| O | -4.24118600 | -0.02186900 | -3.21639000 |
| C | -3.87880000 | -0.40062300 | -4.57816500 |
| C | -4.41560100 | 1.90375100 | -0.34236100 |

| | | | |
|---|---|---|---|
| C | -4.21122900 | 2.24115500 | 1.00585100 |
| C | -3.40375700 | 3.32850800 | 1.38585500 |
| C | -2.75721900 | 4.07251800 | 0.40670300 |
| C | -2.92639300 | 3.73758900 | -0.95371500 |
| C | -3.74623100 | 2.67495000 | -1.31965200 |
| C | -1.81585700 | 5.26597400 | 0.62308500 |
| C | -2.13161000 | 4.65872000 | -1.89150100 |
| C | -2.57561000 | 6.09276900 | -1.56145200 |
| C | -2.40363000 | 6.42129600 | -0.20225500 |
| C | -2.75606200 | 7.68398500 | 0.26848900 |
| C | -3.28447900 | 8.62660600 | -0.62915800 |
| C | -3.45543600 | 8.30044100 | -1.97838400 |
| C | -3.09980100 | 7.02658600 | -2.45212600 |
| C | -0.49926500 | 4.87354900 | -0.06458500 |
| C | 0.76828900 | 4.81324300 | 0.51120000 |
| C | 1.89547200 | 4.43417400 | -0.24806200 |
| C | 1.69426700 | 4.12546700 | -1.60643300 |
| C | 0.42146100 | 4.18353900 | -2.20102500 |
| C | -0.67264400 | 4.55410000 | -1.42580500 |
| C | 3.28530600 | 4.38436200 | 0.38006300 |
| C | 3.68938200 | 3.02399700 | 0.93979300 |
| C | 4.39363200 | 2.09030300 | 0.14791500 |

| | | | |
|---|---|---|---|
| C | 4.76447200 | 0.84791300 | 0.66223000 |
| C | 4.43783200 | 0.50848400 | 1.99035300 |
| C | 3.75443000 | 1.41319300 | 2.79743100 |
| C | 3.38197000 | 2.66171700 | 2.26724500 |
| C | 4.90114400 | -0.89956700 | 2.38555000 |
| C | 5.53369800 | -0.26832100 | -0.06229100 |
| C | 6.76020200 | -0.57925900 | 0.81095100 |
| C | 6.41733800 | -0.92500200 | 2.13317100 |
| C | 7.40864900 | -1.24192000 | 3.05891900 |
| C | 8.75482900 | -1.21286100 | 2.65794500 |
| C | 9.09533900 | -0.86899500 | 1.34584200 |
| C | 8.09459600 | -0.54993200 | 0.41283700 |
| C | 4.27766100 | -1.84474200 | 1.34684700 |
| C | 3.44634100 | -2.93204400 | 1.59602200 |
| C | 2.95662900 | -3.72901800 | 0.53692900 |
| C | 3.34070800 | -3.38740700 | -0.76958100 |
| C | 4.17796500 | -2.28854700 | -1.03370000 |
| C | 4.63756300 | -1.51220600 | 0.02348000 |
| C | 2.05008600 | -4.92198400 | 0.81645800 |
| C | 0.56530100 | -4.58811000 | 0.90625000 |
| C | -0.02412400 | -4.24814700 | 2.14138700 |
| C | -1.40376600 | -3.98660500 | 2.24323000 |

| | | | |
|---|---|---|---|
| C | -2.19620100 | -4.05627800 | 1.09939100 |
| C | -1.62789800 | -4.38707700 | -0.14560100 |
| C | -0.26098900 | -4.65292800 | -0.23233800 |
| C | -2.66602800 | -4.44263200 | -1.27877500 |
| C | -3.71315900 | -3.82296600 | 1.02352400 |
| C | -4.30691600 | -5.10747800 | 0.42274900 |
| C | -3.74162400 | -5.44207300 | -0.82371200 |
| C | -4.16729600 | -6.57659400 | -1.51009500 |
| C | -5.16657100 | -7.38548000 | -0.94332500 |
| C | -5.72707600 | -7.05397500 | 0.29416900 |
| C | -5.29757500 | -5.90858800 | 0.98539100 |
| C | -3.34833800 | -3.06651500 | -1.28278500 |
| C | -3.44754000 | -2.18760700 | -2.36194000 |
| C | -4.09991700 | -0.95147300 | -2.18602300 |
| C | -4.64814500 | -0.58338200 | -0.93893200 |
| C | -4.55270000 | -1.50078900 | 0.12518100 |
| C | -3.90817700 | -2.72791400 | -0.03531900 |
| C | -5.34073900 | 0.76029800 | -0.75170400 |
| H | -6.69383200 | 2.30743700 | 2.08754700 |
| H | -5.55504800 | 2.91801800 | 3.31231300 |
| H | 2.64784900 | 5.06982700 | -3.91210300 |
| H | 2.18555800 | 3.39948700 | -4.33011800 |

| | | | |
|---|---|---|---|
| H | 3.83046000 | 3.44384700 | 4.74676200 |
| H | 2.18225100 | 2.79120000 | 4.93400100 |
| H | 4.32876100 | -5.57275200 | -2.06687700 |
| H | 4.47034800 | -4.17579900 | -3.16052400 |
| H | 1.20609700 | -3.69324200 | 5.16870500 |
| H | -0.42377900 | -3.24261400 | 4.63551600 |
| H | -4.35872100 | -1.34582700 | -4.85277500 |
| H | -2.79116400 | -0.51369600 | -4.66612300 |
| H | -3.30075800 | 3.57939800 | 2.43722100 |
| H | -3.88749000 | 2.41935000 | -2.36445700 |
| H | -1.68633400 | 5.52241900 | 1.67660300 |
| H | -2.26234400 | 4.40626900 | -2.94566100 |
| H | -2.62596900 | 7.94002200 | 1.31570400 |
| H | -3.56203000 | 9.61241600 | -0.27177500 |
| H | -3.86582200 | 9.03396700 | -2.66407700 |
| H | -3.23506700 | 6.77594400 | -3.50008500 |
| H | 0.91338800 | 5.06731100 | 1.55635500 |
| H | 0.28772000 | 3.94727400 | -3.25096500 |
| H | 4.02153100 | 4.67834600 | -0.37282000 |
| H | 3.32303000 | 5.11933900 | 1.18836600 |
| H | 4.63802200 | 2.35848800 | -0.87539100 |
| H | 3.51453900 | 1.15584900 | 3.82328600 |

| | | | |
|---|---|---|---|
| H | 4.63784000 | -1.16322200 | 3.41143800 |
| H | 5.79993200 | -0.00274400 | -1.08770700 |
| H | 7.14794700 | -1.50934000 | 4.07848200 |
| H | 9.53360200 | -1.45826100 | 3.37197500 |
| H | 10.13755400 | -0.84755200 | 1.04577000 |
| H | 8.36236000 | -0.28232100 | -0.60517300 |
| H | 3.15869300 | -3.18984400 | 2.60972800 |
| H | 4.45999900 | -2.05457000 | -2.05586900 |
| H | 2.18604800 | -5.66356500 | 0.02511600 |
| H | 2.36210800 | -5.37995900 | 1.75862400 |
| H | -1.86044000 | -3.76280900 | 3.20024700 |
| H | 0.18907800 | -4.93811500 | -1.17943400 |
| H | -2.22933200 | -4.70504700 | -2.24509400 |
| H | -4.15443700 | -3.56611400 | 1.98883300 |
| H | -3.73475000 | -6.83622800 | -2.47185100 |
| H | -5.50480500 | -8.27129200 | -1.47018700 |
| H | -6.49908900 | -7.68315700 | 0.72399000 |
| H | -5.73603600 | -5.65340000 | 1.94551000 |
| H | -3.04291600 | -2.46756000 | -3.32757000 |
| H | -5.00146800 | -1.23360500 | 1.07810400 |
| H | -5.83394900 | 1.03490900 | -1.68793100 |
| H | -6.11803100 | 0.64263600 | 0.00766500 |

| | | | |
|---|---|---|---|
| C | -1.60499200 | -0.06345000 | 1.16087300 |
| C | -1.01631600 | -0.43695600 | -0.03688400 |
| C | 0.25333400 | 0.06164700 | -0.38111600 |
| C | 0.87711800 | 0.94998300 | 0.51419800 |
| C | 0.25020200 | 1.30458800 | 1.69738400 |
| N | -0.96759500 | 0.78360900 | 2.01464900 |
| C | 0.92586600 | -0.33425700 | -1.64218200 |
| C | 1.80949200 | 0.54455200 | -2.30101400 |
| C | 2.45334600 | 0.14664600 | -3.46055600 |
| N | 2.23922400 | -1.10133200 | -3.97106600 |
| C | 1.40783700 | -1.98461700 | -3.35004100 |
| C | 0.73535400 | -1.61294200 | -2.19371100 |
| C | -1.61000600 | 1.11434600 | 3.31913200 |
| C | 2.92667100 | -1.51077400 | -5.23082500 |
| H | -2.58593300 | -0.40972500 | 1.45053400 |
| H | -1.57076800 | -1.09483200 | -0.69276100 |
| H | 1.85763400 | 1.35913200 | 0.31568900 |
| H | 0.69542000 | 2.00181800 | 2.39488700 |
| H | 2.01785700 | 1.54152500 | -1.93612100 |
| H | 3.15143200 | 0.80555600 | -3.97549200 |
| H | 1.36832500 | -2.98546300 | -3.76596600 |
| H | 0.12370300 | -2.35437100 | -1.69581800 |

| | | | |
|---|---|---|---|
| H | -1.31380500 | 0.36868100 | 4.06136500 |
| H | -1.27890500 | 2.10195500 | 3.63753400 |
| H | -2.69336400 | 1.11300700 | 3.18682900 |
| H | 3.03356500 | -2.59702800 | -5.23038300 |
| H | 2.32715600 | -1.19128000 | -6.08721900 |
| H | 3.90362800 | -1.02969700 | -5.27285900 |
| C | -0.20821300 | -5.32785900 | 5.15772700 |
| H | -0.96193000 | -5.76955100 | 4.48871900 |
| H | 0.60170000 | -6.05952400 | 5.28748200 |
| O | -0.79395600 | -4.95749700 | 6.42608400 |
| C | -1.24204100 | -6.08431800 | 7.22841300 |
| H | -1.64706600 | -5.65832800 | 8.14627300 |
| H | -2.02449400 | -6.65521600 | 6.71045700 |
| H | -0.40715400 | -6.75463600 | 7.47145500 |
| C | -6.38255700 | 0.98013800 | 3.77000200 |
| H | -5.55071600 | 0.63324100 | 4.40389100 |
| H | -6.79606300 | 0.10710200 | 3.24253000 |
| O | -7.39253300 | 1.64772700 | 4.55207900 |
| C | -8.06308500 | 0.79762100 | 5.52097800 |
| H | -7.35371200 | 0.39914900 | 6.25932900 |
| H | -8.79254900 | 1.43310400 | 6.02229600 |
| H | -8.57987200 | -0.03647100 | 5.02738600 |

| | | | |
|---|---|---|---|
| C | 2.27634900 | 4.93781400 | 4.96959100 |
| H | 1.28038500 | 5.15037400 | 4.54988400 |
| H | 2.95658900 | 5.74245300 | 4.65651000 |
| O | 2.22392100 | 4.81756400 | 6.40626800 |
| C | 1.93594800 | 6.06143600 | 7.10192400 |
| H | 0.94996000 | 6.45567800 | 6.82050100 |
| H | 1.94298600 | 5.81938900 | 8.16437700 |
| H | 2.70213200 | 6.81920300 | 6.89194600 |
| C | -4.35136700 | 0.71823300 | -5.49083500 |
| H | -3.98379500 | 1.69078700 | -5.12995000 |
| H | -5.44908300 | 0.75587200 | -5.51822900 |
| O | -3.80211600 | 0.40197200 | -6.78987600 |
| C | -4.30974000 | 1.23525000 | -7.86746400 |
| H | -4.05014000 | 2.29126400 | -7.71129600 |
| H | -3.83422700 | 0.87313400 | -8.77899200 |
| H | -5.39965000 | 1.14232200 | -7.96045500 |
| C | 4.30431100 | 3.73807400 | -4.23557100 |
| H | 5.02825800 | 4.21242700 | -3.56116800 |
| H | 4.43802600 | 4.15447900 | -5.24445000 |
| C | 2.98724600 | -5.62904500 | -3.73917800 |
| H | 2.20844700 | -6.24285000 | -3.26880300 |
| H | 3.67312100 | -6.29114800 | -4.28586400 |

| | | | |
|---|---|---|---|
| O | 4.53022100 | 2.29602700 | -4.26898700 |
| O | 2.37127600 | -4.68880300 | -4.67647000 |
| C | 1.71001800 | -5.36139200 | -5.79554900 |
| H | 0.90134300 | -6.00988000 | -5.43826300 |
| H | 1.29842200 | -4.57629300 | -6.43052100 |
| H | 2.42901300 | -5.95810000 | -6.36879300 |
| C | 5.88906500 | 1.95004200 | -4.67211600 |
| H | 6.62332800 | 2.37404800 | -3.97682500 |
| H | 5.95444600 | 0.86103200 | -4.64842400 |
| H | 6.10139200 | 2.30789400 | -5.68710400 |

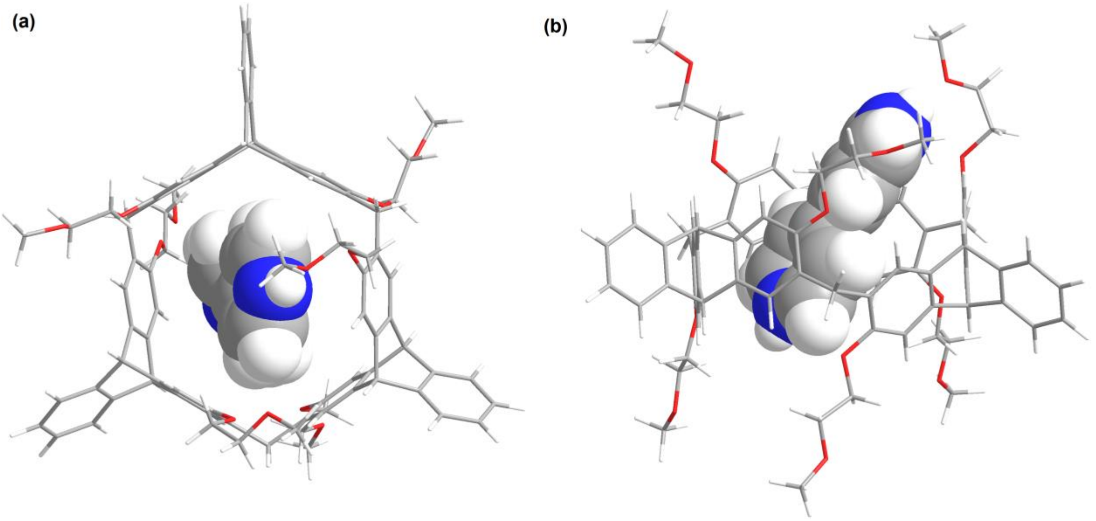


**Figure S93:** Calculated structure of the complex **H4·G3** at the B3LYP/6-31G level. (a) Top view and (b) side view of the complex.

The atomic coordinates of **H4·G3**:

| | | | |
|---|---|---|---|
| O | 5.26468000 | 0.31224600 | 2.20246000 |
| C | 5.70101900 | -0.17118700 | 3.51553000 |
| O | -1.26586100 | -4.19316300 | -2.32959200 |

| | | | |
|---|---|---|---|
| C | -1.23759200 | -4.29673400 | -3.79060600 |
| O | -1.10723800 | -4.13279200 | 2.90473900 |
| C | -1.23691200 | -4.17610200 | 4.37128900 |
| O | -4.39288300 | 2.66658100 | -2.28591200 |
| C | -5.57154700 | 2.80822600 | -3.15180700 |
| O | -2.69273800 | 3.60496300 | 2.89601400 |
| C | -2.31277800 | 3.65506900 | 4.30874900 |
| O | 4.31687600 | 1.72731000 | -3.00397300 |
| C | 4.01902500 | 2.05302100 | -4.39692700 |
| C | 4.94833000 | -0.13259100 | -0.12127600 |
| C | 4.93367200 | -0.61294200 | 1.20521400 |
| C | 4.53731900 | -1.93234700 | 1.50869500 |
| C | 4.13759200 | -2.77827300 | 0.47220300 |
| C | 4.15400000 | -2.31845400 | -0.85896300 |
| C | 4.55607200 | -1.01633800 | -1.14661800 |
| C | 3.65872400 | -4.23519700 | 0.59645000 |
| C | 3.71760100 | -3.39021000 | -1.86729000 |
| C | 4.63335800 | -4.60203800 | -1.62834900 |
| C | 4.60181000 | -5.05898100 | -0.29607600 |
| C | 5.37520300 | -6.14904800 | 0.09553400 |
| C | 6.18758600 | -6.78837700 | -0.85583900 |
| C | 6.21934200 | -6.33496300 | -2.17838300 |

| | | | |
|---|---|---|---|
| C | 5.43837900 | -5.23532600 | -2.57202800 |
| C | 2.27975600 | -4.28416400 | -0.08701200 |
| C | 1.07611900 | -4.73269300 | 0.45921200 |
| C | -0.11789200 | -4.72024700 | -0.29357100 |
| C | -0.04968800 | -4.24943500 | -1.61891800 |
| C | 1.15816800 | -3.81137700 | -2.18930600 |
| C | 2.31802400 | -3.82908700 | -1.41963500 |
| C | -1.43526400 | -5.21000300 | 0.30469600 |
| C | -2.34068000 | -4.10691100 | 0.84601300 |
| C | -3.36988200 | -3.55017000 | 0.05544700 |
| C | -4.17771600 | -2.52500400 | 0.54691200 |
| C | -3.96650700 | -2.01922100 | 1.84523300 |
| C | -2.97455900 | -2.56430500 | 2.65518400 |
| C | -2.17476200 | -3.60570500 | 2.15211000 |
| C | -4.90932600 | -0.86272700 | 2.20374000 |
| C | -5.33795900 | -1.82631600 | -0.17998500 |
| C | -6.56084800 | -1.94926600 | 0.74362000 |
| C | -6.33119400 | -1.42110800 | 2.02974000 |
| C | -7.33734700 | -1.44632800 | 2.99262100 |
| C | -8.58470000 | -2.00378900 | 2.66540500 |
| C | -8.81265600 | -2.52840300 | 1.38921100 |
| C | -7.79717400 | -2.50247600 | 0.41869700 |

| | | | |
|---|---|---|---|
| C | -4.71929800 | 0.18435300 | 1.09363700 |
| C | -4.34073200 | 1.51357500 | 1.24787000 |
| C | -4.22287200 | 2.37818600 | 0.13290000 |
| C | -4.52772800 | 1.84582200 | -1.13125800 |
| C | -4.89817600 | 0.49771400 | -1.29955000 |
| C | -4.97966700 | -0.33451800 | -0.19148700 |
| C | -3.80310300 | 3.83601600 | 0.32577100 |
| C | -2.31530700 | 4.06004600 | 0.59269300 |
| C | -1.77970300 | 3.94298400 | 1.89407700 |
| C | -0.41063500 | 4.15372900 | 2.14369000 |
| C | 0.43198000 | 4.49861000 | 1.08982500 |
| C | -0.08259300 | 4.65516800 | -0.21134500 |
| C | -1.44265100 | 4.44015500 | -0.44561400 |
| C | 0.98439300 | 5.11480700 | -1.21921500 |
| C | 1.93455100 | 4.80666600 | 1.18579000 |
| C | 2.09459400 | 6.24367700 | 0.65915500 |
| C | 1.58118500 | 6.41041600 | -0.64239200 |
| C | 1.64693500 | 7.64977700 | -1.27379400 |
| C | 2.23044500 | 8.73342900 | -0.59563000 |
| C | 2.73952100 | 8.56821200 | 0.69617500 |
| C | 2.67333900 | 7.31676800 | 1.33190700 |
| C | 2.11472200 | 4.07523400 | -1.14893700 |

| | | | |
|---|---|---|---|
| C | 2.65940400 | 3.36350500 | -2.21884000 |
| C | 3.72104000 | 2.46767700 | -1.98342400 |
| C | 4.24189800 | 2.27074600 | -0.68538300 |
| C | 3.68210200 | 3.01650300 | 0.37186400 |
| C | 2.62615000 | 3.90449000 | 0.15252800 |
| C | 5.38937200 | 1.29404400 | -0.44311600 |
| H | 6.41256600 | -0.99417300 | 3.39667600 |
| H | 4.84254400 | -0.52288300 | 4.10093400 |
| H | -0.58670000 | -5.12850000 | -4.08494800 |
| H | -0.85057100 | -3.37031700 | -4.23189600 |
| H | -2.26524900 | -4.42855100 | 4.64462500 |
| H | -0.98016500 | -3.20334800 | 4.80500700 |
| H | -6.23774400 | 3.57102300 | -2.73082700 |
| H | -6.11818600 | 1.86261700 | -3.21111700 |
| H | -3.07643100 | 3.06683600 | 4.81791100 |
| H | -1.34157300 | 3.17806100 | 4.47453900 |
| H | 4.13282900 | 3.12835900 | -4.56851500 |
| H | 2.99325100 | 1.75660400 | -4.64726500 |
| H | 4.57341400 | -2.29905200 | 2.52986900 |
| H | 4.57894900 | -0.65754900 | -2.17047300 |
| H | 3.63577000 | -4.59249900 | 1.62822200 |
| H | 3.74419200 | -3.03627000 | -2.89979700 |

| | | | |
|---|---|---|---|
| H | 5.35365200 | -6.50309400 | 1.12181000 |
| H | 6.79382900 | -7.63802800 | -0.56075900 |
| H | 6.85093200 | -6.83373300 | -2.90565500 |
| H | 5.46670800 | -4.88492400 | -3.59949400 |
| H | 1.03859000 | -5.10773300 | 1.47718300 |
| H | 1.19605600 | -3.46958500 | -3.21774500 |
| H | -1.98597100 | -5.76249700 | -0.46183000 |
| H | -1.20521900 | -5.90643200 | 1.11548100 |
| H | -3.51045200 | -3.92326400 | -0.95339900 |
| H | -2.82015000 | -2.18676400 | 3.66006200 |
| H | -4.73073000 | -0.45996800 | 3.20227000 |
| H | -5.51680400 | -2.23128300 | -1.17829600 |
| H | -7.16328900 | -1.04099100 | 3.98476400 |
| H | -9.37430100 | -2.02757200 | 3.40871300 |
| H | -9.77822300 | -2.95893400 | 1.14628600 |
| H | -7.97723200 | -2.91208700 | -0.57096300 |
| H | -4.12799100 | 1.91904200 | 2.23007900 |
| H | -5.11975700 | 0.11410300 | -2.29056200 |
| H | -4.08110800 | 4.40052700 | -0.56763400 |
| H | -4.37050900 | 4.24641400 | 1.16734800 |
| H | -0.01130800 | 4.07955600 | 3.14816400 |
| H | -1.85982100 | 4.59683500 | -1.43776800 |

| | | | |
|---|---|---|---|
| H | 0.59091400 | 5.24839800 | -2.22975400 |
| H | 2.33076900 | 4.68039100 | 2.19529100 |
| H | 1.25390600 | 7.78152500 | -2.27756900 |
| H | 2.28618000 | 9.70287100 | -1.07903700 |
| H | 3.18946500 | 9.40982400 | 1.21174400 |
| H | 3.07087300 | 7.19154500 | 2.33458300 |
| H | 2.28572900 | 3.52402000 | -3.22350400 |
| H | 4.10814700 | 2.90345500 | 1.36574500 |
| H | 6.01729100 | 1.26803400 | -1.33785800 |
| H | 5.99871200 | 1.66644200 | 0.38298100 |
| C | 1.80157300 | 0.23903600 | 1.00619700 |
| C | 1.07305300 | 0.57431000 | -0.12110400 |
| C | -0.04370500 | -0.20231500 | -0.48893900 |
| C | -0.40651400 | -1.29141500 | 0.32480000 |
| C | 0.35628700 | -1.61344900 | 1.43629400 |
| N | 1.43342900 | -0.84321400 | 1.73979700 |
| C | -0.84798900 | 0.14002700 | -1.68548400 |
| C | -1.56492600 | -0.83938300 | -2.40144900 |
| C | -2.39631600 | -0.45489200 | -3.44706300 |
| N | -2.50435300 | 0.85607400 | -3.77455600 |
| C | -1.79340200 | 1.81714700 | -3.13905400 |
| C | -0.94612800 | 1.48239000 | -2.09861900 |

| | | | |
|---|---|---|---|
| H | 2.67142200 | 0.78866900 | 1.33002900 |
| H | 1.40432000 | 1.41823600 | -0.71003600 |
| H | -1.28053800 | -1.88962000 | 0.11147000 |
| H | 0.14230900 | -2.46265400 | 2.07451100 |
| H | -1.51678000 | -1.89415800 | -2.15452100 |
| H | -2.97316000 | -1.18927500 | -3.99897700 |
| H | -1.97647400 | 2.83440600 | -3.44607500 |
| H | -0.43867900 | 2.27477500 | -1.56688800 |
| C | -2.29743900 | 5.08207900 | 4.84739000 |
| H | -1.65312900 | 5.73442400 | 4.23891600 |
| H | -3.31250900 | 5.50382300 | 4.83940700 |
| O | -1.78746600 | 4.95887700 | 6.19368300 |
| C | -1.84008600 | 6.18890200 | 6.96717300 |
| H | -1.43865000 | 5.94345300 | 7.95028200 |
| H | -1.22849400 | 6.97577700 | 6.50535000 |
| H | -2.87251800 | 6.54793700 | 7.07054600 |
| C | 6.34933600 | 1.00303300 | 4.22943300 |
| H | 5.67576100 | 1.87368900 | 4.22483200 |
| H | 7.28453000 | 1.29028500 | 3.72841400 |
| O | 6.59035600 | 0.53263900 | 5.57202800 |
| C | 7.33871000 | 1.45990100 | 6.40489300 |
| H | 6.79785700 | 2.40785400 | 6.52917100 |

| | | | |
|---|---|---|---|
| H | 7.44834500 | 0.97212200 | 7.37317200 |
| H | 8.33074900 | 1.66190400 | 5.98001900 |
| C | -0.26521400 | -5.23011400 | 4.87615200 |
| H | 0.73825800 | -5.06153200 | 4.45336200 |
| H | -0.59539400 | -6.23550400 | 4.57865200 |
| O | -0.26086200 | -5.07617300 | 6.30992900 |
| C | 0.47226900 | -6.10962600 | 7.02326700 |
| H | 1.53419800 | -6.11032100 | 6.74204000 |
| H | 0.37497500 | -5.86931800 | 8.08161000 |
| H | 0.04571500 | -7.10267500 | 6.83034900 |
| C | 5.00074300 | 1.27245200 | -5.25434700 |
| H | 4.97927800 | 0.20432700 | -4.98986200 |
| H | 6.02429700 | 1.64218300 | -5.10197700 |
| O | 4.55980800 | 1.48374800 | -6.61369500 |
| C | 5.47276700 | 0.96950500 | -7.62120200 |
| H | 5.59132700 | -0.11905900 | -7.53282000 |
| H | 5.02518100 | 1.21128600 | -8.58512200 |
| H | 6.45826800 | 1.44710100 | -7.54375000 |
| C | -2.64852700 | -4.54907800 | -4.26866000 |
| H | -3.09352000 | -5.37640400 | -3.70147100 |
| H | -2.62839400 | -4.82504900 | -5.33314400 |
| C | -5.11806600 | 3.24086800 | -4.52731900 |

| | | | |
|---|---|---|---|
| H | -4.48417500 | 4.13414700 | -4.47195100 |
| H | -6.00054600 | 3.47122100 | -5.13667300 |
| O | -3.44705700 | -3.34004500 | -4.08954800 |
| O | -4.36945800 | 2.15677100 | -5.18631000 |
| C | -4.25582100 | 2.32198900 | -6.63950600 |
| H | -3.73969500 | 3.25638200 | -6.88521700 |
| H | -3.67932700 | 1.47203100 | -7.00594600 |
| H | -5.25049700 | 2.31598700 | -7.09599100 |
| C | -4.84622200 | -3.53756200 | -4.45646500 |
| H | -5.30471200 | -4.32225400 | -3.84280800 |
| H | -5.35262300 | -2.58896600 | -4.27225800 |
| H | -4.94230700 | -3.80483900 | -5.51628100 |
| H | 2.03122100 | -1.11776700 | 2.51282600 |
| H | -3.24647400 | 1.21276300 | -4.43130900 |

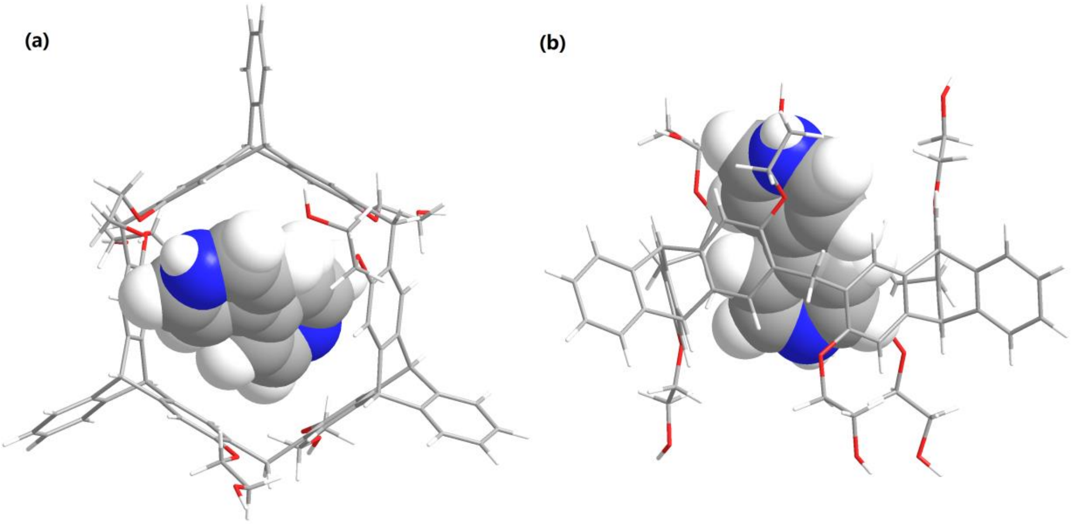


**Figure S94:** Calculated structure of the complex **H5·G3** at the B3LYP/6-31G level. (a) Top view and (b) side view of the complex.

The atomic coordinates of **H5·G3**:

| | | | |
|---|---|---|---|
| O | 5.08143200 | 0.83413100 | 2.59092600 |
| C | 5.42356400 | 0.37487000 | 3.93821100 |
| O | -0.63183700 | -4.27307500 | -2.41226900 |
| C | -0.50180700 | -4.34731700 | -3.86774100 |
| O | -0.82720600 | -4.25298000 | 2.81812500 |
| C | -1.02533100 | -4.27791100 | 4.27582200 |
| O | -4.46811600 | 2.24563800 | -2.55049200 |
| C | -5.62571400 | 2.31345900 | -3.45291700 |
| O | -3.20572100 | 3.30850800 | 2.73793000 |
| C | -2.93790600 | 3.40182300 | 4.17331700 |
| O | 4.35157200 | 2.20926200 | -2.66292000 |
| C | 4.09398400 | 2.48895900 | -4.07271100 |
| C | 4.96628500 | 0.39254300 | 0.24881400 |
| C | 4.90986900 | -0.10550300 | 1.56803300 |
| C | 4.63071800 | -1.46294200 | 1.83312400 |
| C | 4.39023500 | -2.32972900 | 0.76459400 |
| C | 4.44973300 | -1.85174600 | -0.55875800 |
| C | 4.73643100 | -0.51131100 | -0.80745000 |
| C | 4.05759100 | -3.82978100 | 0.84510400 |
| C | 4.19473900 | -2.94828800 | -1.60272100 |
| C | 5.21286000 | -4.06277500 | -1.31021400 |

| | | | |
|---|---|---|---|
| C | 5.13944700 | -4.53922700 | 0.01352600 |
| C | 5.99391300 | -5.54919000 | 0.44915300 |
| C | 6.92998500 | -6.08800900 | -0.44939800 |
| C | 7.00279300 | -5.61548800 | -1.76359700 |
| C | 6.14037900 | -4.59638300 | -2.20154000 |
| C | 2.74084600 | -4.01085600 | 0.06821700 |
| C | 1.55698100 | -4.59012100 | 0.52754100 |
| C | 0.42281700 | -4.69419500 | -0.30590200 |
| C | 0.53229200 | -4.20466300 | -1.62166400 |
| C | 1.72227000 | -3.63326400 | -2.10465200 |
| C | 2.82163600 | -3.53657000 | -1.25580400 |
| C | -0.87528500 | -5.32297400 | 0.19712500 |
| C | -1.92096300 | -4.32276900 | 0.68480600 |
| C | -2.94910800 | -3.86331700 | -0.16765400 |
| C | -3.88298000 | -2.92607400 | 0.27386400 |
| C | -3.80320900 | -2.41348500 | 1.58396600 |
| C | -2.81640400 | -2.86683300 | 2.45466700 |
| C | -1.88900000 | -3.82140600 | 2.00102500 |
| C | -4.87720400 | -1.36105400 | 1.88724000 |
| C | -5.06217200 | -2.34154300 | -0.52095400 |
| C | -6.31962200 | -2.59634900 | 0.32655900 |
| C | -6.22194500 | -2.05948500 | 1.62583200 |

| | | | |
|---|---|---|---|
| C | -7.27543600 | -2.19662200 | 2.52663400 |
| C | -8.43734800 | -2.87587800 | 2.12364400 |
| C | -8.53437900 | -3.40881000 | 0.83429200 |
| C | -7.47128500 | -3.26973600 | -0.07367600 |
| C | -4.72880900 | -0.29035000 | 0.79375000 |
| C | -4.49630800 | 1.06848800 | 0.97586700 |
| C | -4.40446400 | 1.95213900 | -0.12637000 |
| C | -4.58113800 | 1.40453800 | -1.40801000 |
| C | -4.80092800 | 0.02752600 | -1.60471900 |
| C | -4.85897700 | -0.82061600 | -0.50693700 |
| C | -4.15168000 | 3.44349500 | 0.09609000 |
| C | -2.71723100 | 3.81737000 | 0.46659800 |
| C | -2.26432000 | 3.74515200 | 1.80200100 |
| C | -0.94481800 | 4.09210100 | 2.14677700 |
| C | -0.07129700 | 4.53110000 | 1.15493300 |
| C | -0.50755400 | 4.64621800 | -0.17883700 |
| C | -1.81870900 | 4.29485200 | -0.50771200 |
| C | 0.57387400 | 5.22232300 | -1.10855000 |
| C | 1.38122200 | 4.99026900 | 1.35668000 |
| C | 1.42831900 | 6.44119100 | 0.84589800 |
| C | 0.99273600 | 6.56677400 | -0.48823700 |
| C | 0.97362400 | 7.81232300 | -1.11050300 |

| | | | |
|---|---|---|---|
| C | 1.39200400 | 8.94384200 | -0.38996200 |
| C | 1.82276300 | 8.81964100 | 0.93455600 |
| C | 1.84286100 | 7.56189900 | 1.56091700 |
| C | 1.79712400 | 4.30335000 | -0.96140800 |
| C | 2.48513200 | 3.66165000 | -1.99243700 |
| C | 3.61303800 | 2.87497400 | -1.68523400 |
| C | 4.05861500 | 2.71821100 | -0.35406700 |
| C | 3.35327700 | 3.39302500 | 0.66311700 |
| C | 2.23132400 | 4.17297800 | 0.37217100 |
| C | 5.28000000 | 1.86117200 | -0.03202900 |
| H | 6.23099700 | -0.36261800 | 3.89366500 |
| H | 4.55111600 | -0.08003200 | 4.42381700 |
| H | 0.24146400 | -5.10837500 | -4.13334000 |
| H | -0.18077100 | -3.38041700 | -4.27395200 |
| H | -2.04388700 | -4.60141800 | 4.50805600 |
| H | -0.86158200 | -3.28080000 | 4.69957500 |
| H | -6.34581200 | 3.04291500 | -3.06243800 |
| H | -6.11554600 | 1.33763800 | -3.51998200 |
| H | -3.66928900 | 2.73720300 | 4.63342100 |
| H | -1.93609600 | 3.03360200 | 4.41578800 |
| H | 4.11270200 | 3.56797400 | -4.25765200 |
| H | 3.11536600 | 2.09158900 | -4.36816600 |

| | | | |
|---|---|---|---|
| H | 4.63744300 | -1.84060000 | 2.85072800 |
| H | 4.79225100 | -0.13621000 | -1.82411300 |
| H | 4.00207600 | -4.20252200 | 1.87016200 |
| H | 4.25417000 | -2.57881000 | -2.62841000 |
| H | 5.94090000 | -5.91794400 | 1.46909400 |
| H | 7.60001900 | -6.87468600 | -0.11973500 |
| H | 7.72966700 | -6.03660600 | -2.44982900 |
| H | 6.20086700 | -4.23065800 | -3.22225500 |
| H | 1.48978900 | -4.97901100 | 1.53872000 |
| H | 1.79408400 | -3.27767800 | -3.12660200 |
| H | -1.31585400 | -5.91483600 | -0.60995000 |
| H | -0.63165400 | -6.00592800 | 1.01546500 |
| H | -2.98963700 | -4.24029600 | -1.18409300 |
| H | -2.76487200 | -2.48453300 | 3.46816500 |
| H | -4.80081300 | -0.95119800 | 2.89587600 |
| H | -5.13733700 | -2.75381300 | -1.52950400 |
| H | -7.20309200 | -1.78500500 | 3.52877100 |
| H | -9.26279800 | -2.98756300 | 2.81840100 |
| H | -9.43447000 | -3.93362200 | 0.53251500 |
| H | -7.54975200 | -3.68606600 | -1.07375600 |
| H | -4.38362700 | 1.48345500 | 1.97068700 |
| H | -4.92597200 | -0.36294600 | -2.60990500 |

| | | | |
|---|---|---|---|
| H | -4.42360000 | 3.98352000 | -0.81413400 |
| H | -4.81569800 | 3.78702500 | 0.89604600 |
| H | -0.60928300 | 4.04930900 | 3.17618600 |
| H | -2.18015000 | 4.41849000 | -1.52615300 |
| H | 0.23994400 | 5.32414800 | -2.14390500 |
| H | 1.71762700 | 4.89576400 | 2.39104600 |
| H | 0.64029800 | 7.91248200 | -2.13920400 |
| H | 1.38062600 | 9.91855200 | -0.86577400 |
| H | 2.14491100 | 9.69830200 | 1.48295300 |
| H | 2.17955600 | 7.46878700 | 2.58902400 |
| H | 2.16824500 | 3.79557900 | -3.02022800 |
| H | 3.71669000 | 3.31210300 | 1.68457500 |
| H | 5.97207100 | 1.91102400 | -0.87713400 |
| H | 5.78468100 | 2.28394700 | 0.83904600 |
| C | 1.71899500 | 0.42296100 | 1.09970900 |
| C | 1.03457700 | 0.69803800 | -0.07061200 |
| C | 0.02941400 | -0.18308000 | -0.51692000 |
| C | -0.27006900 | -1.31577600 | 0.26305500 |
| C | 0.44616200 | -1.57155600 | 1.42182000 |
| N | 1.41605700 | -0.69958100 | 1.80113100 |
| C | -0.72972000 | 0.09163500 | -1.75980600 |
| C | -1.30664600 | -0.94562100 | -2.51923600 |

| | | | |
|---|---|---|---|
| C | -2.10569000 | -0.63372800 | -3.61307100 |
| N | -2.31579200 | 0.66289300 | -3.94637600 |
| C | -1.74040500 | 1.68274800 | -3.26670300 |
| C | -0.93118200 | 1.42194500 | -2.17578700 |
| H | 2.50649900 | 1.05276600 | 1.48244400 |
| H | 1.31634000 | 1.57955800 | -0.62975800 |
| H | -1.05933800 | -1.99946100 | -0.01372200 |
| H | 0.27977400 | -2.44528800 | 2.04011100 |
| H | -1.17534100 | -1.99295000 | -2.27146600 |
| H | -2.57838000 | -1.41496400 | -4.19828500 |
| H | -1.99658300 | 2.68089100 | -3.58438400 |
| H | -0.53542600 | 2.25471900 | -1.61184500 |
| C | -3.11282300 | 4.82397700 | 4.69551900 |
| H | -2.49956400 | 5.53029400 | 4.11972100 |
| H | -4.16325000 | 5.12762500 | 4.60537400 |
| O | -2.67825500 | 4.74705400 | 6.07750700 |
| C | 5.85270300 | 1.59755100 | 4.73086800 |
| H | 5.09189400 | 2.38513700 | 4.64075400 |
| H | 6.80189900 | 1.98668300 | 4.34213600 |
| O | 5.96991000 | 1.11589600 | 6.09215500 |
| C | -0.00224100 | -5.24251400 | 4.85249100 |
| H | 0.99516900 | -5.01318400 | 4.45048100 |

| | | | |
|---|---|---|---|
| H | -0.25500600 | -6.27388400 | 4.57727400 |
| O | -0.06435500 | -5.02002900 | 6.28209500 |
| C | 5.18351100 | 1.79364400 | -4.87076200 |
| H | 5.25435500 | 0.73992100 | -4.56860300 |
| H | 6.15270500 | 2.27220900 | -4.68544000 |
| O | 4.75938600 | 1.92412900 | -6.25205000 |
| C | -1.85144800 | -4.72235800 | -4.43281900 |
| H | -2.23167300 | -5.61159500 | -3.91890500 |
| H | -1.74286700 | -4.94618200 | -5.50177400 |
| C | -5.14309500 | 2.76012800 | -4.81348200 |
| H | -4.56295900 | 3.68503900 | -4.73426700 |
| H | -6.00682200 | 2.93289800 | -5.46341000 |
| O | -2.76805700 | -3.59244700 | -4.24490600 |
| O | -4.29256200 | 1.70772700 | -5.41536600 |
| H | 0.35746100 | -5.74720200 | 6.77861400 |
| H | -2.92453900 | 5.55078200 | 6.57446600 |
| H | 6.42432400 | 1.76420700 | 6.66345900 |
| H | -3.68351400 | -3.87414400 | -4.43873600 |
| H | 5.49538100 | 1.74356300 | -6.86757200 |
| H | -4.33011000 | 1.73833900 | -6.38954300 |
| H | 1.98760800 | -0.92161600 | 2.61028000 |
| H | -3.04934000 | 0.94086600 | -4.64634000 |
